# Morpholine-based chalcones as dual-acting monoamine oxidase-B and acetylcholinesterase inhibitors: synthesis and biochemical investigations

**DOI:** 10.1080/14756366.2020.1842390

**Published:** 2021-01-12

**Authors:** Rani Sasidharan, Bo Hyun Eom, Jeong Hyun Heo, Jong Eun Park, Mohamed A. Abdelgawad, Arafa Musa, Nicola Gambacorta, Orazio Nicolotti, Sreedharannair Leelabaiamma Manju, Bijo Mathew, Hoon Kim

**Affiliations:** a College of Pharmaceutical Science, Government T.D. Medical College, Alappuzha, India; b Organic Chemistry Division, SAS, VIT University, Vellore, India; c Department of Pharmacy, and Research Institute of Life Pharmaceutical Sciences, Sunchon National University, Suncheon, Republic of Korea; d Pharmaceutical Chemistry Department, College of Pharmacy, Jouf University, Sakaka, Saudi Arabia; e Pharmaceutical Organic Chemistry Department, Faculty of Pharmacy, Beni-Suef University, Beni Suef, Egypt; f Department of Pharmacogonosy, College of Pharmacy, Jouf University, Sakaka, Saudi Arabia; g Department of Pharmacogonosy, Al-Azhar University, Cairo, Egypt; h Dipartimento di Farmacia—Scienze del Farmaco, Università degli Studi di Bari “Aldo Moro”, Bari, Italy; i Division of Drug Design and Medicinal Chemistry Research Lab, Department of Pharmaceutical Chemistry, Ahalia School of Pharmacy, Palakkad, India

**Keywords:** Morpholine-containing chalcone, monoamine oxidase, acetylcholinesterase, dual-acting inhibitor, Docking analysis

## Abstract

Nine compounds (**MO1–MO9**) containing the morpholine moiety were assessed for their inhibitory activities against monoamine oxidases (MAOs) and acetylcholinesterase (AChE). Most of the compounds potently inhibited MAO-B; **MO1** most potently inhibited with an IC_50_ value of 0.030 µM, followed by **MO7** (0.25 µM). **MO5** most potently inhibited AChE (IC_50_ = 6.1 µM), followed by **MO9** (IC_50_ = 12.01 µM) and **MO7** most potently inhibited MAO-A (IC_50_ = 7.1 µM). **MO1** was a reversible mixed-type inhibitor of MAO-B (*K_i_* = 0.018 µM); **MO5** reversibly competitively inhibited AChE (*K_i_* = 2.52 µM); and **MO9** reversibly noncompetitively inhibited AChE (*K_i_* = 7.04 µM). **MO1**, **MO5** and **MO9** crossed the blood–brain barrier, and were non-toxic to normal VERO cells. These results show that **MO1** is a selective inhibitor of MAO-B and that **MO5** is a dual-acting inhibitor of AChE and MAO-B, and that both should be considered candidates for the treatment of Alzheimer’s disease.

## 1. Introduction

Due to the extreme complexities of brain systems and their diverse dysfunctions, research focus is being directed towards the design of multi-target directed ligands (MTDLs)^1^. In fact, it has been suggested that functional equilibrium of brain after a neurologic disorder is unlikely to be achieved by focussing on a single molecular target^2^. The major challenge presented by the development of MTDLs is to preserve balance between the effects drugs have by acting at their individual molecular targets^3^. On the other hand, some ligands can target two or more specific entities within numerous biological networks. The likelihood of successful MTDL design can be enhanced by considering the design of molecular scaffolds via suitable molecular hybridisation and by understanding the pathophysiologies of multifaceted diseases. The design process can be accelerated by selecting pharmacophores based on pre-clinical studies, emphasising structure–activity relationships (SARs) and by performing *in silico*-based virtual screening^4^.

Monoamine oxidases (MAOs) play prominent roles in the inactivations of various biogenic amines in central and peripheral tissues. The isoenzymes of MAO-A and MAO-B are considered major therapeutic targets in various neuropsychiatric illnesses and neurodegenerative disorders like Alzheimer’s disease (AD) and Parkinson’s disease (PD)^5^. During the degradations of various biogenic neurotransmitters catalysed by MAO-A and MAO-B, hydrogen peroxide and reactive oxygen species (ROS) are produced as major by-products, which might cause oxidative damage in brain tissues. MAO inhibitors are considered potential neuroprotective agents and up-regulating agents for neurotransmitter amines^6^. Numerous studies have documented that selective and reversible/irreversible MAO-B inhibitors are likely to play pivotal roles in AD-related therapeutic strategies. Acetylcholinesterase (AChE) and butyrylcholinesterase (BChE) inhibitors also play significant roles in the maintenance of cholinergic functions and are used to provide symptomatic relief in AD. Over the past two decades, many researchers have tried to develop multi-acting MAO-B and AChE/BChE inhibitors^7–10^.

**Figure 1.**
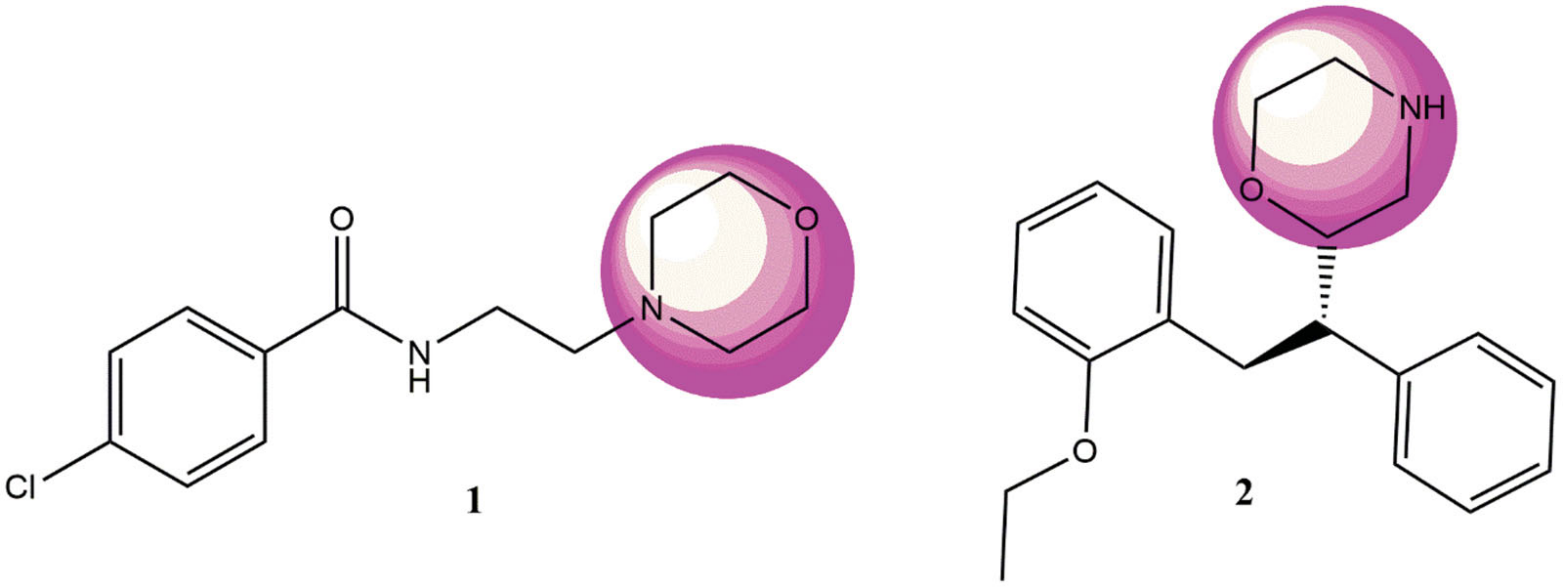
Morpholine-containing, CNS active, FDA approved drugs. 1, moclobemide; 2, reboxetine.

Morpholine is a tetrahydro-1,4-oxazine with a saturated heterocyclic ring and provides a promising developmental starting point due to its biological profile with metabolic stability. The presence of a heteroatom like oxygen or nitrogen facilitates hydrogen bonding, and thus, interactions with enzymes, and the presence of electron-deficient atoms may also impart hydrophobic interactions with morpholine. From the synthetic perspective, various molecular scaffolds have been added to morpholine by replacing its secondary nitrogen^11^. Moclobemide (1) and reboxetine (2) (both antidepressants) provide examples of FDA-approved drugs containing the morpholine moiety (Figure 1). These drugs reversibly inhibit MAO-A and selectively inhibit norepinephrine reuptake in the central nervous system (CNS), and thus, block the human a4b2 nicotinic acetylcholine receptor. In addition, more than 20 drugs containing the morpholine moiety have been FDA approved; they include mycophenolate mofetil (an immunosuppressant), linezolid and finafloxacin (antibiotics), geftinib (an antineoplastic and epidermal growth factor inhibitor), rivaroxaban (an anticoagulant and factor Xa inhibitor), and eteplirsen, which is used to treat Duchenne muscular dystrophy^12^. Considering the importance of morpholine nucleus, it is worthwhile to design morpholine derived compounds of medicinal chemistry interest.

**Scheme 1.**
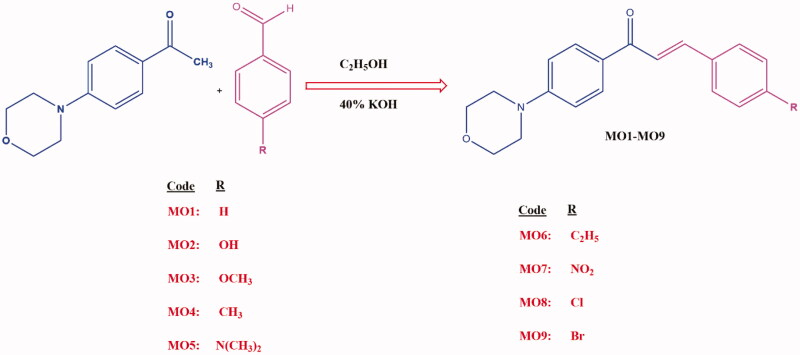
The synthetic route used to produce the target compounds (**MO1–MO9**).

**Figure 2.**
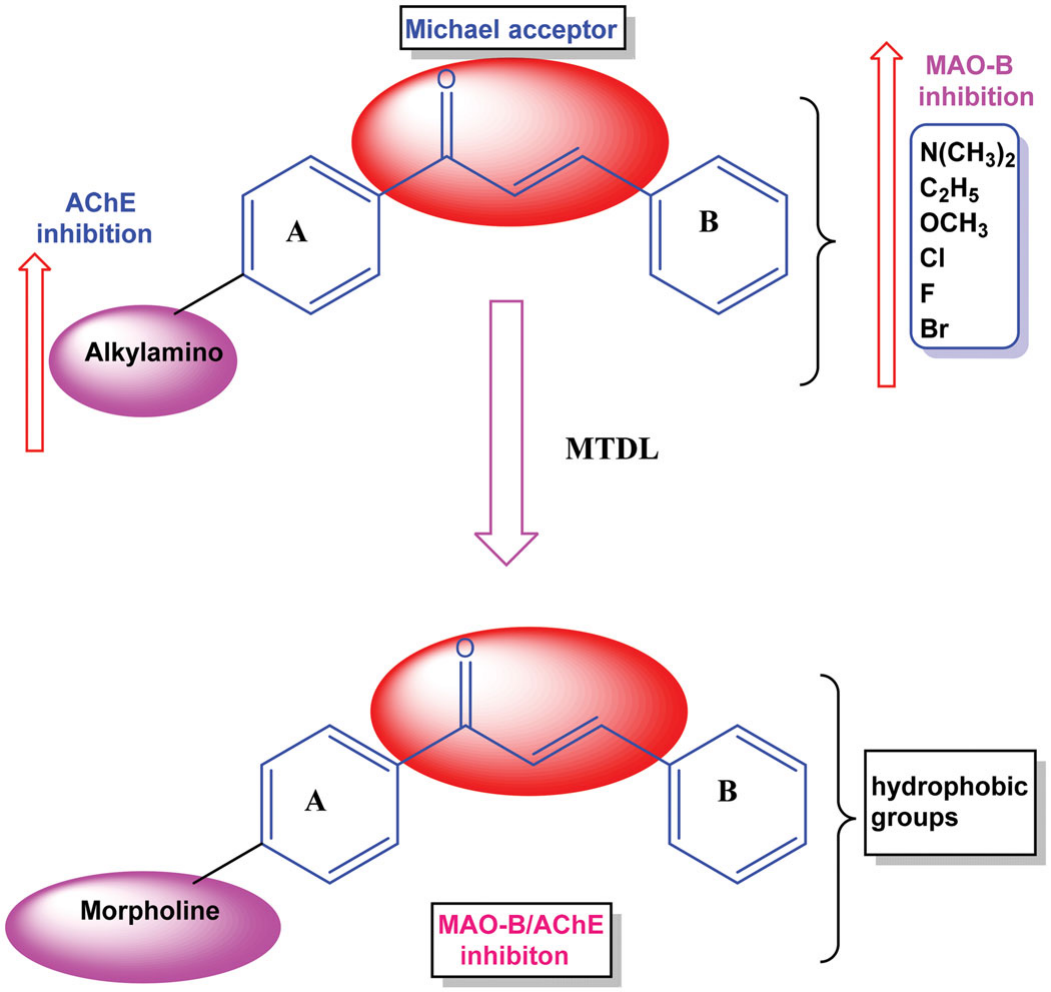
MTDL design of MAO-B and AChE inhibitors.

The chalcones framework is considered an excellent starting point for the design for MAO-B and AChE enzyme inhibitors^13–15^. The presence of an α-β unsaturated ketone and three rotatable bonds in chalcones can provide different bonding orientations^16^. The literature amply demonstrates that most chalcones are potent, reversible and selective MAO-B inhibitors^17^. In addition, the presence and orientation of various electron-donating and withdrawing groups on the phenyl/heteroaryl **A** and **B** rings of chalcones can impart electrophilic character to the Michael acceptor present. The introductions of electron-donating/lipophilic halogens onto the phenyl **B** ring of chalcones have resulted in the syntheses of highly selective MAO-B inhibitors^18–27^. Many studies have reported that the presence of various alkylamino groups on the **A** ring provides AChE inhibitory activity^28–31^. The FDA-approved drugs like flurbiprofen and rivastigmine linked with chalcone moiety were developed as selective ChE/MAO-B inhibitors for the prophylactic agents for AD^32^. The design strategy of the current study is depicted in Figure 2 and the synthetic route is shown in Scheme 1. A recent study showed that the presence of a pendant morpholine ring on the chalcone **A** ring favoured hMAO-B inhibitory activity^33^^,^^34^. The difference in MAO-B inhibition profiles became more obvious when chalcone **B** ring was substituted using a fluoro or trifluoromethyl group^33^.

## 2. Results and discussion

### 2.1. Chemistry

Morpholine-containing α,β-unsaturated ketones were prepared by condensation between various aromatic/*para* substituted aldehydes and 4-morpholine acetophenone in the presence of an alcoholic basic medium^33^. The H^1^ NMR showed that the ring protons N-CH_2_ and O-CH_2_ of morpholine ring resonated at 3.32–3.31 and 3.75–3.88 ppm as triplets, respectively. The large coupling constant (*J =* 15 Hz) value of Hα and Hβ protons confirmed the *trans* configuration of the morpholine chalcones. The deshielded values between 186.88–186.07 in C^13^-NMR clearly evidenced the formation of sp^2^ carbonyl functional group of α,β-unsaturated ketones. The identities of the compounds were determined by ^1^H-NMR, ^13 ^C-NMR and Mass spectrometry (Supplementary materials).

### 2.2. Analysis of enzyme inhibitory activities

#### 2.2.1. Inhibitory activities

In this study, nine morpholine-based compounds (**MO1**–**MO9**) were analysed with respect to their abilities to inhibit MAO-A, MAO-B and AChE. Most of the compounds inhibited MAO-B by ∼ 50% at 1.0 µM, except **MO3** and **MO5** (Table 1). Compound **MO1** most potently inhibited MAO-B (IC_50_ = 0.030 µM), followed by **MO7**, **MO8**, **MO4**, **MO9**, **MO6**, **MO2**, **MO3** and **MO5** (IC_50_ = 0.25, 0.32, 0.33, 0.36, 0.64, 0.70, 1.01 and 1.31 µM, respectively). In addition, **MO5** most potently inhibited AChE (IC_50_ = 6.1 µM), followed by **MO9** and **MO8** (IC_50_ = 12.01 and 12.07 µM, respectively). **MO7** most potently inhibited MAO-A (IC_50_ = 7.1 µM), followed by **MO6** (IC_50_ = 8.7 µM). **MO5** most potently inhibited BChE (IC_50_ = 18.09 µM), with weaker inhibitory activity compared to AChE, followed by **MO7** (IC_50_ = 24.83 µM) (Table 1).

**Table 1.** Inhibitions of MAO-A, MAO-B, and AChE by **MO1–MO9**^a^

Compounds	Residual activities (%)				IC_50_ (µM)				SI^b^
	MAO-A (10 µM)	MAO-B (1.0 µM)	AChE (10 µM)	BChE (10 µM)	MAO-A	MAO-B	AChE	BChE	
**MO1**	94.2 ± 0.62	38.1 ± 9.50	53.4 ± 0.99	62.85 ± 2.97	>40	0.030 ± 0.062	16.1 ± 2.24	>40	>1333.3
**MO2**	93.8 ± 1.34	54.5 ± 5.28	72.3 ± 1.34	63.55 ± 3.30	>40	0.70 ± 0.23	30.2 ± 3.24	>40	> 57.1
**MO3**	91.0 ± 3.17	79.9 ± 5.28	79.3 ± 2.82	56.78 ± 2.97	>40	1.01 ± 0.08	>40	>40	>39.6
**MO4**	70.2 ± 1.24	49.0 ± 1.45	66.0 ± 8.79	80.30 ± 6.43	25.8 ± 1.58	0.33 ± 0.03	28.42 ± 0.02	>40	78.2
**MO5**	88.1 ± 3.43	58.6 ± 3.39	34.5 ± 9.51	60.86 ± 1.07	>40	1.31 ± 0.26	6.1 ± 0.0048	18.09 ± 0.38	>30.5
**MO6**	12.3 ± 1.58	64.1 ± 1.37	76.9 ± 3.54	70.96 ± 0.36	8.7 ± 1.32	0.64 ± 0.04	>40	>40	13.6
**MO7**	−16.5 ± 4.53	26.1 ± 5.44	65.3 ± 1.55	70.21 ± 4.76	7.1 ± 0.41	0.25 ± 0.05	20.48 ± 1.10	24.83 ± 0.34	28.4
**MO8**	83.7 ± 4.02	34.6 ± 5.44	50.6 ± 3.61	74.61 ± 0.73	>40	0.32 ± 0.16	12.07 ± 1.18	>40	>125.0
**MO9**	79.8 ± 1.54	51.3 ± 2.42	50.4 ± 2.42	70.21 ± 5.50	>40	0.36 ± 0.16	12.01 ± 2.13	>40	>111.1
Toloxatone					1.08 ± 0.025	—			
Lazabemide					—	0.063 ± 0.015			
Clorgyline					0.007 ± 0.00070	—			
Pargyline					—	0.028 ± 0.0043			
Tacrine							0.27 ± 0.019	0.060 ± 0.0022	

^a^ Values above are the means ± SEs of duplicate or triplicate experiments.

^b^ SI = IC_50_ of MAO-A/IC_50_ of MAO-B.

AChE values ​were determined after pre-incubation compounds and enzymes for 15 min.

**Figure 3.**
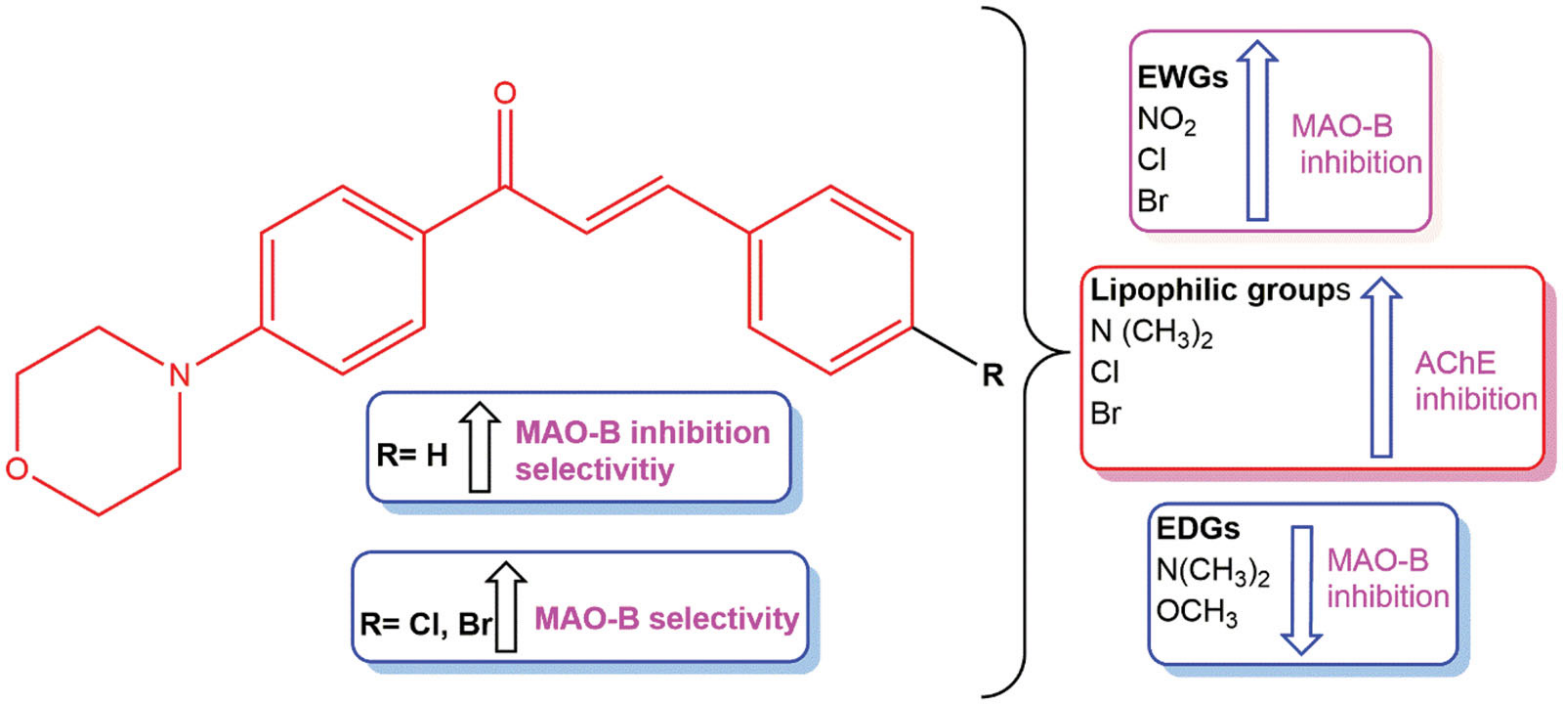
SAR analysis of MAO-B and AChE inhibitors.

Morpholine-containing compounds in this study shared a 1–(4-morpholinophenyl) prop-2-en-1-one structure. Introduction of various electron-donating and withdrawing groups onto the *para* position of the phenyl **B** ring of the basic chalcone scaffold afforded different derivatives. Surprisingly, unsubstituted **MO1** inhibited MAO-B more potently than the other eight derivatives. All nine derivatives exhibited MAO-B selectivity. In particular, **MO1** (IC_50_ = 0.030 µM) was ∼2 times more potent than the reversible MAO-B inhibitor lazabemide (IC_50_ = 0.063 µM), and had a high selectivity index (SI = >1333.3), which meant that it was the best inhibitor in the series. Regarding MAO-B inhibition, **MO1** was slightly less potent than the reference irreversible MAO-B inhibitor pargyline (IC_50_ = 0.028 µM). The presence of dimethylamino, chloro and bromo substituents at the *para* position of the chalcone **B** ring conferred moderate AChE inhibition. Recently, it was reported that shifting of morpholine ring to the phenyl **B** ring of chalcones showed moderate AChE inhibition^35^. Results of SAR analysis of MAO-B/AChE inhibitions by the nine compounds are provided in Figure 3.

#### 2.2.2. Kinetics of MAO-B inhibition

**Figure 4.**
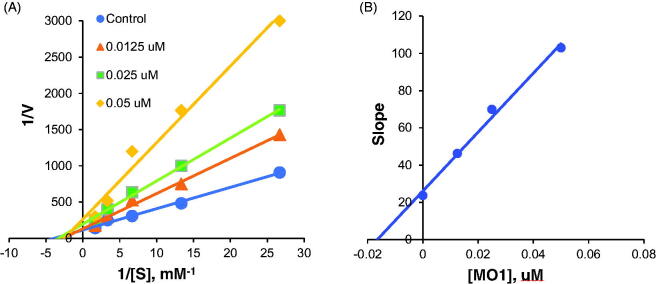
Lineweaver–Burk plots of MAO-B inhibition by **MO1** (A), and respective secondary plots (B) of slopes vs. inhibitor concentrations.

Lineweaver–Burk plots and secondary plots of MAO-B inhibition by **MO1** showed that **MO1** is a mixed inhibitor of MAO-B (*K_i_* = 0.018 ± 0.002) (Figure 4(A,B)). These results suggested that **MO1** binds to an allosteric site other than the substrate-binding site of MAO-B.

#### 2.2.3. Kinetics of AChE inhibitions

**Figure 5.**
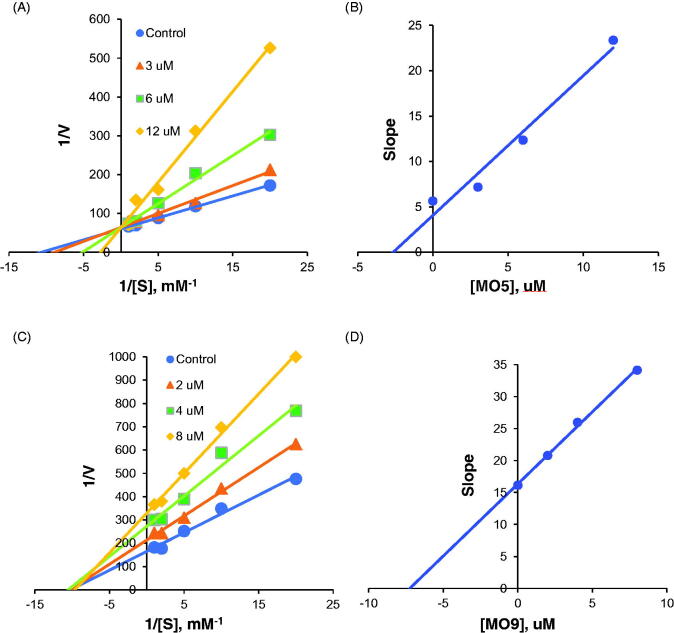
Lineweaver–Burk plots of AChE inhibition by **MO5** (A) and **MO9** (C), and respective secondary plots (B and D) of slopes vs. inhibitor concentrations.

Lineweaver–Burk plots and secondary plots showed that **MO5** and **MO9** competitively and non-competitively, respectively, inhibited AChE (Figure 5(A,C)) with *K_i_* values of 2.52 ± 0.17 and 7.04 ± 0.32 µM, respectively (Figure 5(B,D)), which suggest that **MO5** is a potent, selective and competitive inhibitor of AChE that binds to the active site of AChE, and that **MO9** binds to a site other than the active site of AChE and changes the 3D structure of the enzyme, thus inhibiting enzyme activity.

#### 2.2.4. Analysis of the reversibilities of MAO-B and AChE inhibitions

**Figure 6.**
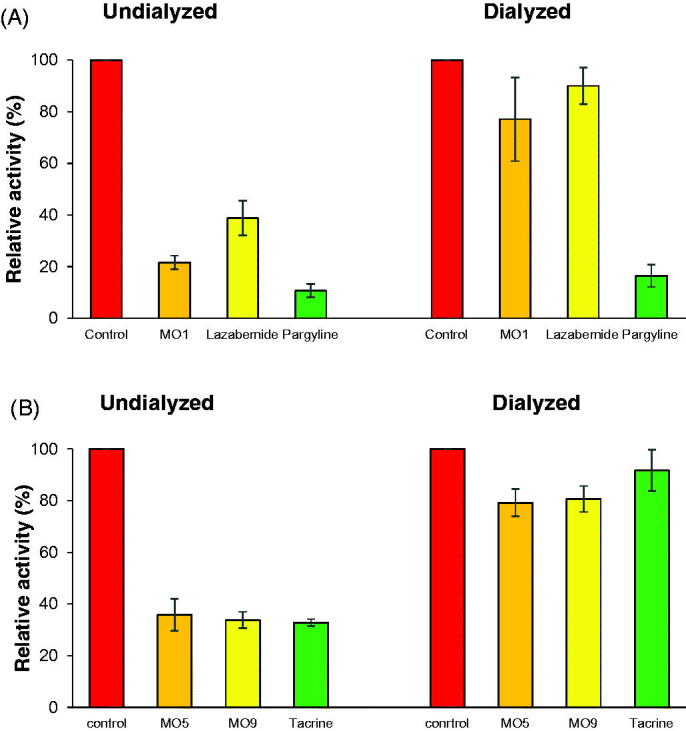
Dialysis recoveries of MAO-B inhibition by **MO1** (A) and AChE inhibition by **MO5** and **MO9** (B). The concentrations of the inhibitors used were approximately 2 × IC_50_ values: **MO1**, 0.06 µM; **MO5**, 12.0 µM; **MO9**, 24.0 µM; lazabemide, 0.12 µM; pargyline, 0.06 µM; and tacrine, 0.54 µM (tacrine was used as a reference reversible inhibitor). For recovery experiments, preincubated enzyme mixtures were dialysed as described in the text.

Inhibition assays were carried out after preincubating MAO-B or AChE with the inhibitors for 15 min. The reversibilities of MAO-B inhibition by **MO1** and of AChE inhibitions by **MO5** and **MO9** were investigated by dialysis^34^. Dialysis recovered the inhibition of MAO-B by **MO1** from 21.6% (*A*_U_) to 77.1% (*A*_D_), which was similar to that shown by lazabemide (from 38.8 to 90.0%), a reversible MAO-B inhibitor (Figure 6(A)). On the other hand, inhibition of MAO-B by pargyline (an irreversible inhibitor) was recovered from 10.7 to 16.5%. In addition, inhibitions of AChE by **MO5** and **MO9** were recovered by dialysis from 35.8% (A_U_) to 79.2% (A_D_) and from 33.8 to 80.7%, respectively, which were similar to that observed for tacrine (from 32.8 to 91.7%), a reversible AChE inhibitor (Figure 6(B)). These results indicate that **MO1** is a reversible inhibitor of MAO-B, and that **MO5** and **MO9** are reversible inhibitors of AChE.

### 2.3. Blood–brain barrier (BBB) permeation

The synthesised derivatives were screened for their abilities to cross the BBB using the parallel artificial membrane permeability assay (PAMPA) because this ability is a critical developmental requirement for any drug targeting neurodegenerative disorders or depression. The PAMPA used was validated by comparing the experimentally determined permeabilities of eight commercial drugs with reported values (Table 2). According to reported BBB permeation limits, compounds were classified as follows:^36^ CNS+ (high): Pe (10^−6 ^cm s^−1^) > 4.00, CNS- (low): Pe (10^−6 ^cm s^−1^) <2.00 and CNS± (uncertain): Pe (10^−6 ^cm s^−1^) from 4.00 to 2.00. Our results suggest that all nine synthesised derivatives could cross the BBB, and that **MO1**, **MO5** and **MO9** would be the most effective.

**Table 2.** Experimental membrane permeabilities of the synthetic and reference inhibitors.

Compounds	Bibliography Pe (×10^−6^ cm s^−1^)	Experimental Pe (×10^−6^ cm s^−1^)	Prediction
Testosterone	17.0	16.86 ± 0.67	CNS+
Verapamil	16.0	15.69 ± 0.44	CNS+
β-estradiol	12.0	11.88 ± 0.53	CNS+
Progesterone	9.3	9.10 ± 0.11	CNS+
Piroxicam	2.5	2.35 ± 0.30	CNS+/-
Hydrocortisone	1.8	1.71 ± 0.05	CNS-
Lomefloxacin	1.1	1.26 ± 0.01	CNS-
Dopamine	0.2	0.21 ± 0.01	CNS-
**MO1**	—	16.34 ± 0.16	CNS+
**MO5**	—	14.44 ± 0.81	CNS+
**MO9**	—	14.61 ± 0.08	CNS+

### 2.4. Cytotoxic studies

The biocompatibilities of **MO1, MO5** and **MO9** were investigated using an MTT (3–(4,5-dimethylthiazol-2-yl)-2,5-diphenyltetrazolium bromide) assay and normal VERO cells^37^,^38^. Results showed that **MO1, MO5** and **MO9** were non-toxic to normal VERO cells with IC_50_ values of 195.14, 185.44 and 188.34 µg/mL, respectively, indicating that the therapeutic potentials of these compounds would be suitable.

### 2.5. Total ROSs assay

**Figure 7.**
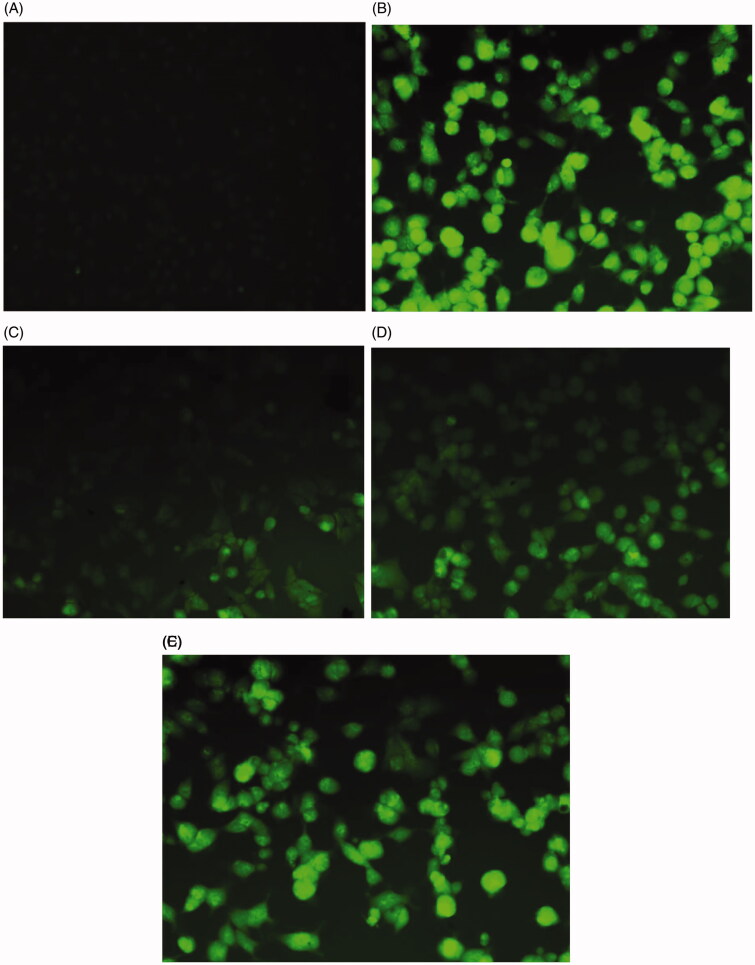
Inhibition of H_2_O_2_-induced ROS generation in human cervical cancer cells. (A) Treatment-naïve control cells; (B) Cells treated with 100 μg/mL of H_2_O_2_; (C) Cells treated with 40 μg/mL of **MO1**; (D) Cells treated with 40 μg/mL of **MO5**; (E) Cells- treated with 40 μg/mL of **MO9**.

ROS are considered to be the radicals primarily responsible for the neuronal death observed in various neurodegenerative disorders^39^. The lead compounds **MO1, MO5** and **MO9** were subjected to ROS assays using Hela cells. ROS levels significantly increased in H_2_O_2_-treated Hela cells as determined by fluorescent image analyses (Figure 7(B)). **MO1** and **MO5** (Figure 7(C,D)) at 40 µg/mL resulted in significantly lower ROS levels than **MO9** (Figure 7(E)). Interestingly, the colony formation induced by H_2_O_2_ was markedly reduced by the lead MAO-B inhibitor **MO1**, which suggested that the molecule can be highly recommended for the arrest of free radicals in the neurodegenerative disorders.

### 2.6. Computational studies

Initially, **MO1** and **MO5** were used as bases for profiling the pharmacological spectra of putative protein targets using the free web platform MuSSel^40^^,^^41^. Interestingly, MAO-B and AChE were predicted to be 13th and 14th targets for **MO1** and 7th and 15th targets for **MO5**, respectively. Details are provided in Supporting Information.

**Figure 8.**
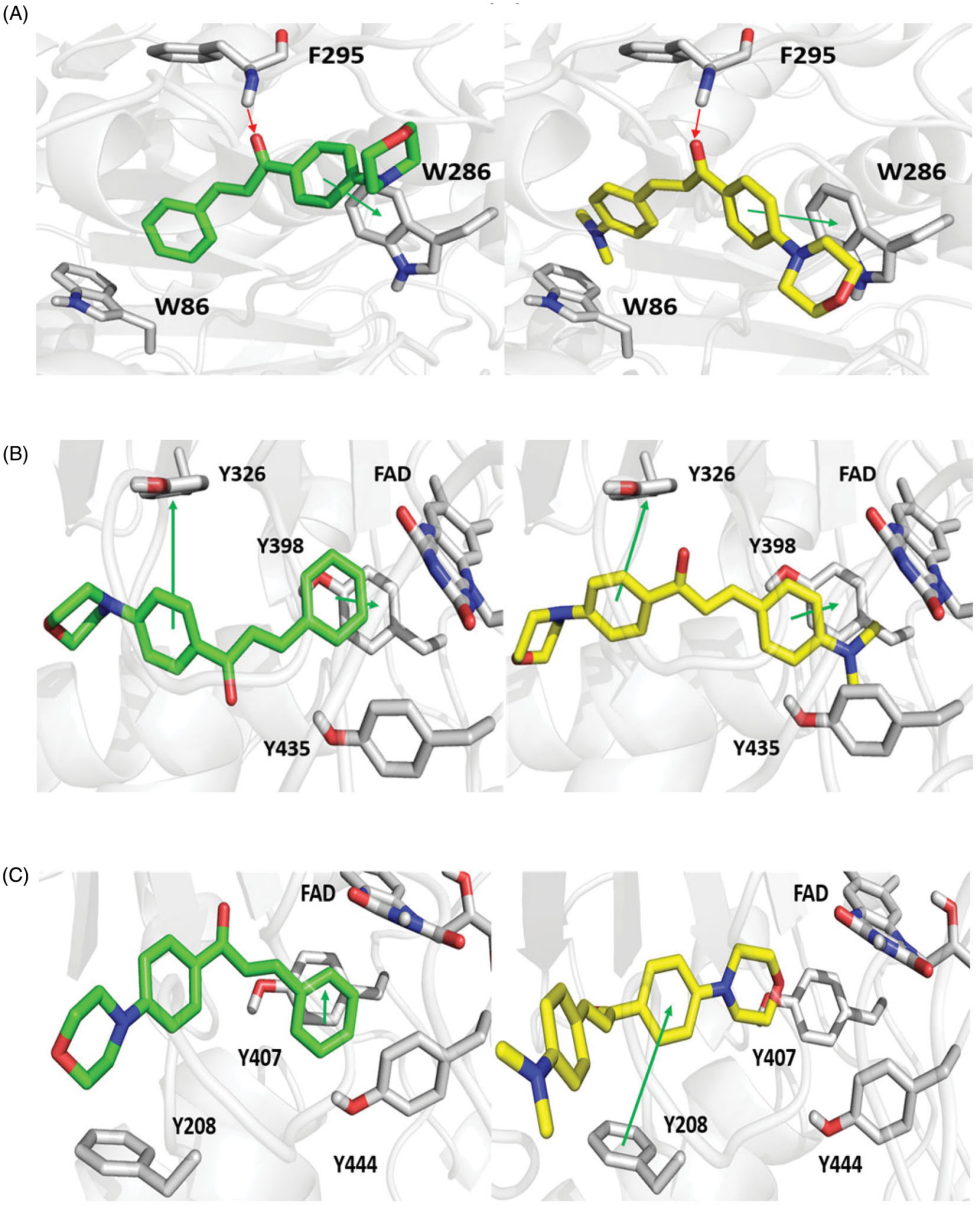
Top scored poses of **MO1** and **MO5** for binding with AChE (A), MAO-B (B) and MAO-A (C) are shown on left and right sides, respectively. **MO1**, **MO5** and key enzyme residues are displayed as green, yellow and grey sticks, respectively. Green and red arrows indicate π–π interactions and hydrogen bonds, respectively.

Docking studies were performed on **MO1** and **MO5** using the X-ray resolved structures of MAO-A, MAO-B and AChE in the Protein Data Bank (PDB) as entries 2Z5X, 2VZ5 and 4EY7^42–45^. Docking protocols were performed as we described in a previous study^13^. Molecular docking simulations were performed to investigate interactions between **MO1** and **MO5** and MAO-A, MAO-B and AChE. The docking scores resulting from *in silico* simulations are reported in Table 3. Results showed that the carbonyl and the 4-morpholin-phenyl groups of the chalcone scaffolds of **MO1** and **MO5** hydrogen-bonded with F295 and formed π − π interactions with the side chain of W286 at the peripheral anionic subsite (PAS) of AChE (Figure 8(A)). Also, the dimethylamino group of **MO5** established a weak hydrophobic interaction with W86 at the catalytic anionic subsite (CAS). **MO1** and **MO5** formed π − π interactions with the selective MAO-B residue Y326 (Figure 8(B)). In addition, the styryl and dimethylaminophenyl groups of **MO1** and **MO5**, respectively, established π–π interactions with Y398 and were sandwiched within an aromatic cage formed by Y328, Y435 and FAD. **MO1** engaged in a π − π interaction with Y407 and was entangled in a hydrophobic cage consisting of Y407, Y444 and FAD of MAO-A (Figure 8(C)), whereas **MO5** established π–π interactions with a selective MAO-A residue F208 using its 4-morpholine-phenyl group. In order to point out the selectivity of **MO1** and **MO5**, molecular docking analyses were performed on BChE crystal structure. Docking score values of **MO1** and **MO5** for BChE were worse than those for AChE (Table 3). Interestingly, **MO1** and **MO5** can predominantly establish hydrophobic interactions in the BChE binding pocket; in particular **MO1** engaged a π–π interaction with F329, but both compounds cannot make crucial interactions with the catalytic W82 residue (Figure 8(D)).

**Table 3.** Docking score values for bindings between **MO1** and **MO5** and MAO-B or AChE.

Compounds	Docking score (kcal/mol)		
	AChE	MAO-B	BChE
**MO1**	−10.31	−8.67	−7.42
**MO5**	−10.12	−9.34	−6.66

Overall, computational analyses provided a sound explanation of the experimental data. Docking scores and the binding modes obtained demonstrated that **MO1** and **MO5** can meaningfully interact with MAO-B as indicated by IC_50_ experimental values. Interestingly, even if **MO1** had adopted a similar binding mode in MAO-A and MAO-B binding pockets, the two docking score values would have been quite different, due to a lack of π–π interaction with the MAO-A selective residue F208 and lesser hydrophobic interactions. On the other hand, **MO5** assumed a switched pose within the MAO-A binding pocket, with its morpholine ring facing FAD, presumably because the dimethylamino substituent on the phenyl ring did not allow adequate access to the binding site. As regards AChE, molecular docking awarded a better score for **MO5** than **MO1**, which also agreed with experimental data, despite the resemblance between their binding poses.

## 3. Conclusion

We describe the synthesis of morpholine-containing α,β-unsaturated ketones, and the results of an investigation of their MAOs and AChE inhibition profiles. Most of the nine compounds synthesised exhibited potent MAO-B inhibition with moderate AChE inhibition. Interestingly, **MO1** (the lead compound) inhibited MAO-B inhibition in the low nanomolar range and was more potent than lazabemide (the reference compound). The low cytotoxicity and the ability of **MO1** to transit the BBB support our drug design strategy. In addition, the ROS scavenging efficacy of **MO1** suggests improved neuroprotective effects.

## 4. Experimental

### 4.1. Enzyme assays

MAO-A activity was measured continuously for 20 min at 316 nm using 0.06 mM kynuramine as substrate, as described previously^46^^,^^47^, whereas MAO-B activity was measured for 30 min at 250 nm using 0.3 mM benzylamine as substrate. MAO activity assays were performed using recombinant human MAO-A or MAO-B. A slightly modified version of the method developed by Ellman^48^ was used to measure AChE and BChE activities, using 0.5 mM acetylthiocholine iodide (ATCI) and *S*-butyrylthiocholine iodide (BTCI), respectively, as substrates for 10 min at 412 nm^49^. Colour development was performed using 0.5 mM 5,5′-dithiobis (2-nitrobenzoic acid) (DTNB), which reacts with thiocholine (a product of ATCI by AChE or of BTCI by BChE) to produce 5-thio 2-nitrobenzoic acid. Preincubation was treated for 15 min before adding ATCI and DTNB.

### 4.2. Analysis of enzyme inhibitions and kinetics

MAO-A, MAO-B, AChE and BChE activities were measured after exposure to inhibitors at a concentration of 10 µM. Inhibitions of MAO-B at 10 µM of these compounds tested were too excessive, and thus, a concentration of 1.0 µM was used. IC_50_ values were determined by measuring the residual enzyme activities. Toloxatone, lazabemide and tacrine, were used as reference reversible inhibitors of MAO-A, MAO-B and AChE, respectively, and clorgyline and pargyline as reference irreversible inhibitors of MAO-A and MAO-B, respectively. *K_i_* values and inhibitor types were determined by kinetic testing, as previously described^50^. Kinetic tests were conducted at 5 different substrate concentrations, and the inhibitor concentrations used were 0, ∼1/2 × IC_50_, IC_50_, and 2 × IC_50_ values. Lineweaver–Burk plots and their secondary plots were used to determine *K_i_* values and inhibitor types.

### 4.3. Analysis of inhibitor reversibilities

Inhibition types (reversible or irreversible) were determined by dialysis, as previously described^51,52^, but by using 0.06 mM kynuramine and 0.3 mM benzylamine. For the experiment of AChE, 0.5 mM ATCI was used. Dialysis experiments were performed by preincubating enzymes and inhibitors (or reference inhibitors) at ∼2 × IC_50_ in 0.1 M sodium phosphate buffer (pH 7.2) for 30 min, and then dialysing solutions with stirring for 6 h with a buffer change at 3 h. Residual activities were calculated using the measured activities of undialyzed (A_U_) and dialysed (A_D_) solutions and the activities of untreated controls (i.e. without inhibitor).

## Supplementary Material

Supplemental Material
