# Supplementary material for: Morpholine-based chalcones as dual-acting monoamine oxidase-B and acetylcholinesterase inhibitors: synthesis and biochemical investigations
Source: J Enzyme Inhib Med Chem. 2021 Jan 12;36(1):188–97. doi: 10.1080/14756366.2020.1842390 (PMC7808749; doi:10.1080/14756366.2020.1842390)
Supplement: Supplemental Material [file IENZ_A_1842390_SM5440.pdf]

Supporting Information

Dual-acting monoamine oxidase-B and acetylcholinesterase inhibitors containing the morpholine group: Synthesis and biochemical investigations

Rani Sasidharan[a, h#], Bo Hyun Eom[b#], Jeong Hyun Heo[b#], Mohamed A Abdelgawad[c,d], Arafa Musa[e,f], Nicola Gambacorta[g], Orazio Nicolotti[g], Sreedharannair Leelabaiamma Manju[h*], Bijo Mathew[i*] and Hoon Kim[b*]

[a]College of Pharmaceutical Science, Government T.D. Medical College, Alappuzha, Kerala, India.

[b]Department of Pharmacy, and Research Institute of Life Pharmaceutical Sciences, Sunchon National University, Suncheon 57922, Republic of Korea.

[c]Pharmaceutical Chemistry Department , College of Pharmacy, Jouf University, Sakaka , Al jouf 72341, Saudi Arabia.

[d]Pharmaceutical Organic Chemistry Department, Faculty of Pharmacy, Beni-Suef university , Beni Suef 62514, Egypt.

[e]Department of Pharmacogonosy, College of Pharmacy, Jouf University, Sakaka, Al Jouf, 2014, Saudi Arabia.

[f]Department of Pharmacogonosy, Al-Azhar University, Cairo-11371, Egypt.

[g]Dipartimento di Farmacia—Scienze del Farmaco, Università degli Studi di Bari "Aldo Moro", via E. Orabona, 4, I-70125 Bari, Italy.

[h]Organic Chemistry Division, SAS, VIT University, Vellore, Tamil Nadu, India.

[i]Division of Drug Design and Medicinal Chemistry Research Lab, Department of Pharmaceutical Chemistry, Ahalia School of Pharmacy, Palakkad-678557, Kerala, India.

[#]Authors contributed equally.

[*]Corresponding Authors:

Hoon Kim (H. Kim) (hoon@sunchon.ac.kr)

Bijo Mathew (B. Mathew) (bijovilaventgu@gmail.com) (bijo.mathew@ahalia.ac.in)

S. L. Manju (Manju S.L.) (slmanju@vit.ac.in)

Table of Contents

Spectral Characterization of the synthesized compounds…………………………………………………… S1

Copies of NMR and Mass spectra …………………………………………………………………………………… S2

MuSSel prediction of the lead molecules……………………………………………………………………… S3

Spectral Characterization of the synthesized compounds………………………………………….. S1

(2*E*)-1-[4-(morpholin-4-yl)phenyl]-3-phenylprop-2-en-1-one (**MO1**): ^{1}H-NMR (500 MHz, DMSO) δ: 3.34–3.33 (4H, t, J = 5.0 Hz, morpholine -N-$(CH_2)_2$), 3.75–3.74 (4H, t, J = 5.0 Hz, morpholine O-$(CH_2)_2$), 7.04–7.02 (2H, d, H3′ & H5′), 7.45–7.43 (3H, m, H3, H4 & H5), 7.69–7.65 (1H, d, J = 15.0 Hz, -CHα), 7.88–7.87 (2H, d, H2&H6), 7.94–7.91 (1H, d, J = 15.0 Hz, -CHβ), 8.09–8.07 (2H, d, H2′&H6′). ^{13}C-NMR (500 MHz, DMSO) δ: 186.46, 153.99, 142.14, 134.90, 130.46, 130.10, 128.76, 128.53, 127.39, 122.10, 112.97, 65.75, 46.62. ESI-MS (*m/z*): Calculated- 293.3596, Observed-293.3593.

(2*E*)-3-(4-hydroxyphenyl)-1-[4-(morpholin-4-yl)phenyl]prop-2-en-1-one (**MO2**): ^{1}H-NMR (500 MHz, DMSO) δ: 3.32–3.31 (4H, t, J = 5.0 Hz, morpholine -N-$(CH_2)_2$), 3.74–3.73 (4H, t, J = 5.0 Hz, morpholine O-$(CH_2)_2$), 6.43 (1H, s, Ar-OH),7.03–7.01 (2H, d, H3′ & H5′), 7.46–7.44 (2H, m, H3 & H5), 7.66–7.63 (1H, d, J = 15.0 Hz, -CHα), 7.87–7.86 (2H, d, H2&H6), 7.96–7.93 (1H, d, J = 15.0 Hz, -CHβ), 8.10–8.08 (2H, d, H2′&H6′). ^{13}C-NMR (500 MHz, DMSO) δ: 186.88, 153.32, 142.44, 134.65, 130.22, 130.11, 128.86, 128.63, 127.59, 122.50, 112.47, 65.65, 46.82. ESI-MS (*m/z*): Calculated- 309.3590, Observed-309.3592.

(2*E*)-3-(4-methoxyphenyl)-1-[4-(morpholin-4-yl)phenyl]prop-2-en-1-one (**MO3**): ^{1}H NMR (500 MHz, DMSO) δ: 3.33–3.32 (4H, t, J = 5.0 Hz, morpholine -N-$(CH_2)_2$), 3.75–3.73 (4H, t, J = 5.0 Hz, morpholine O-$(CH_2)_2$), 3.82 (3H, s, OCH_3), 7.03–7.02 (2H, d, H3′ & H5′), 7.01–7.00 (2H, m, H3, & H5), 7.66–7.63 (1H, d, J = 15.0 Hz, -CHα), 7.81–7.78 (1H, d, J = 15.0 Hz, -CHβ), 7.84–7.82 (2H, d, H2&H6), 8.07–8.05 (2H, d, H2′&H6′).^{13}C-NMR (500 MHz, DMSO) δ: 186.38, 160.89,

153.82, 142.07, 130.34, 130.25, 127.65, 127.49, 119.54, 114.20, 112.94, 65.72, 55.20, 46.63. ESI-MS (*m/z*): Calculated- 323.3856, Observed-323.3853.

(2*E*)-3-(4-methylphenyl)-1-[4-(morpholin-4-yl)phenyl]prop-2-en-1-one (**MO4**): ^{1}H NMR (500 MHz, DMSO) δ: 2.35 (3H, s, OCH_3),3.33–3.32 (4H, t, J = 5.0 Hz, morpholine -N-$(CH_2)_2$), 3.75–3.74 (4H, t, J = 5.0 Hz, morpholine O-$(CH_2)_2$), 7.04–7.02 (2H, d, H3′ & H5′), 7.27–7.25 (2H, m, H3 & H5), 7.65–7.62 (1H, d, J = 15.0 Hz, -CHα), 7.77–7.75 (2H, d, H2&H6), 7.88–7.85 (1H, d, J = 15.0 Hz, -CHβ),8.08–8.06 (2H, d, H2′&H6′). ^{13}C-NMR (500 MHz, DMSO) δ: 186.46, 153.93, 142.20, 140.06, 132.17, 130.38, 129.38, 128.58, 127.50, 121.03, 112.97, 65.75, 46.64, 20.96. ESI-MS (*m/z*): Calculated- 307.3862, Observed-323.3860.

(2*E*)-3-[4-(dimethylamino)phenyl]-1-[4-(morpholin-4-yl)phenyl]prop-2-en-1-one (**MO5**): ^{1}H NMR (500 MHz, DMSO) δ: 2.99 (6H, s, $(NCH_3)_2$),3.32–3.31 (4H, t, J = 5.0 Hz, morpholine -N-$(CH_2)_2$), 3.76–3.75 (4H, t, J = 5.0 Hz, morpholine O-$(CH_2)_2$), 6.75–6.73 (2H, d, H3′ & H5′), 7.02–7.00 (2H, m, H3 & H5), 7.19–7.16 (1H, d, J = 15.0 Hz, -CHα), 7.62–7.61 (2H, d, H2&H6), 7.68–7.65 (1H, d, J = 15.0 Hz, -CHβ), 8.04–8.02 (2H, d, H2′&H6′). ^{13}C-NMR (500 MHz, DMSO) δ: 186.26, 153.65, 151.60, 143.26, 130.30, 130.01, 128.19, 122.25, 116.24, 113.02, 111.66, 65.7, 46.76, 41.16. ESI-MS (*m/z*): Calculated- 336.4274, Observed-336.4272.

(2*E*)-3-(4-ethylphenyl)-1-[4-(morpholin-4-yl)phenyl]prop-2-en-1-one (**MO6**): ^{1}H NMR (500 MHz, DMSO) δ: 1.12-1.18 (3H, t, J = 10.0 Hz, CH_3), 2.67-2.65 (2H, q, J = 10.0 Hz, CH_2), 3.34–3.33 (4H, t, J = 5.0 Hz, morpholine -N-$(CH_2)_2$), 3.75–3.74 (4H, t, J = 5.0 Hz, morpholine O-$(CH_2)_2$), 7.04–7.02 (2H, d, H3′ & H5′), 7.30–7.28 (2H, m, H3 & H5), 7.67–7.64 (1H, d, J = 15.0 Hz, -CHα), 7.79–7.77 (2H, d, H2&H6), 7.89–7.86 (1H, d, J = 15.0 Hz, -CHβ), 8.08–8.06 (2H, d,

H2′&H6′). ^{13}C-NMR (500 MHz, DMSO) δ: 186.48, 153.94, 146.41, 142.23, 132.45, 130.39, 128.68, 128.21, 127.15, 121.11, 112.98, 65.76, 46.65, 28.02, 15.27. ESI-MS (*m/z*): Calculated-321.4128, Observed-321.4125.

(2*E*)-1-[4-(morpholin-4-yl)phenyl]-3-(4-nitrophenyl)prop-2-en-1-one (**MO7**): ^{1}H NMR (500 MHz, DMSO) δ: 3.35–3.34 (4H, t, J = 5.0 Hz, morpholine -N-$(CH_2)_2$), 3.76–3.75 (4H, t, J = 5.0 Hz, morpholine O-$(CH_2)_2$), 7.05–7.03 (2H, d, H3′ & H5′), 7.75–7.72 (1H, d, J = 15.0 Hz, -CHα), 8.11–8.09 (2H, d, H2′&H6′), 8.14–8.11 (1H, d, J = 15.0 Hz, -CHβ), 8.16–8.14 (2H, m, H3 & H5), 8.28–8.26 (2H, d, H2&H6). ^{13}C-NMR (500 MHz, DMSO) δ: 186.07, 154.18, 147.72, 141.51, 139.33, 130.72, 129.54, 126.97, 126.27, 123.79, 112.91, 65.74, 46.53. ESI-MS (*m/z*): Calculated- 338.3578, Observed-338.3570.

(2*E*)-3-(4-chlorophenyl)-1-[4-(morpholin-4-yl)phenyl]prop-2-en-1-one (**MO8**): ^{1}H NMR (500 MHz, DMSO) δ: 3.34–3.33 (4H, t, J = 5.0 Hz, morpholine -N-$(CH_2)_2$), 3.75–3.74 (4H, t, J = 5.0 Hz, morpholine O-$(CH_2)_2$), 7.04–7.02 (2H, d, H3′ & H5′), 7.52–7.50 (2H, d, H2′&H6′),7.66–7.63 (1H, d, J = 15.0 Hz, -CHα), 7.92–7.90 (2H, d, H2&H6)**,** 7.97–7.94 (1H, d, J = 15.0 Hz, -CHβ), 8.09–8.07 (2H, m, H3 & H5). ^{13}C-NMR (500 MHz, DMSO) δ: 186.29, 154.02, 140.65, 134.51, 133.89, 130.51, 130.26, 129.90, 128.77, 127.26, 122.88, 112.88, 65.74, 46.58. ESI-MS (*m/z*): Calculated- 327.8047, Observed-327.8045.

(2*E*)-3-(4-bromophenyl)-1-[4-(morpholin-4-yl)phenyl]prop-2-en-1-one (**MO9**): ^{1}H NMR (500 MHz, DMSO) δ: 3.34–3.33 (4H, t, J = 5.0 Hz, morpholine -N-$(CH_2)_2$), 3.75–3.74 (4H, t, J = 5.0 Hz, morpholine O-$(CH_2)_2$), 7.04–7.02 (2H, d, H3′ & H5′), 7.64–7.61 (1H, d, J = 15.0 Hz, -CHα), 7.66–7.64 (2H, d, H2′&H6′), 7.85–7.83 (2H, d, H2&H6)**,** 7.98–7.95 (1H, d, J = 15.0 Hz, -CHβ),

8.09–8.07 (2H, m, H3, H3 & H5). ^{13}C-NMR (500 MHz, DMSO) δ: 186.28, 154.01, 140.72, 134.20, 131.67, 130.49, 130.47, 127.23, 123.34, 122.12, 112.91, 65.72, 46.55. ESI-MS (*m/z*): Calculated- 372.2557, Observed-372.2554.

Copies of NMR and Mass spectra ……………………………………………………………………………………S2

MO-1
1H_8scan DMSO {D:\Spectra} nmr 6

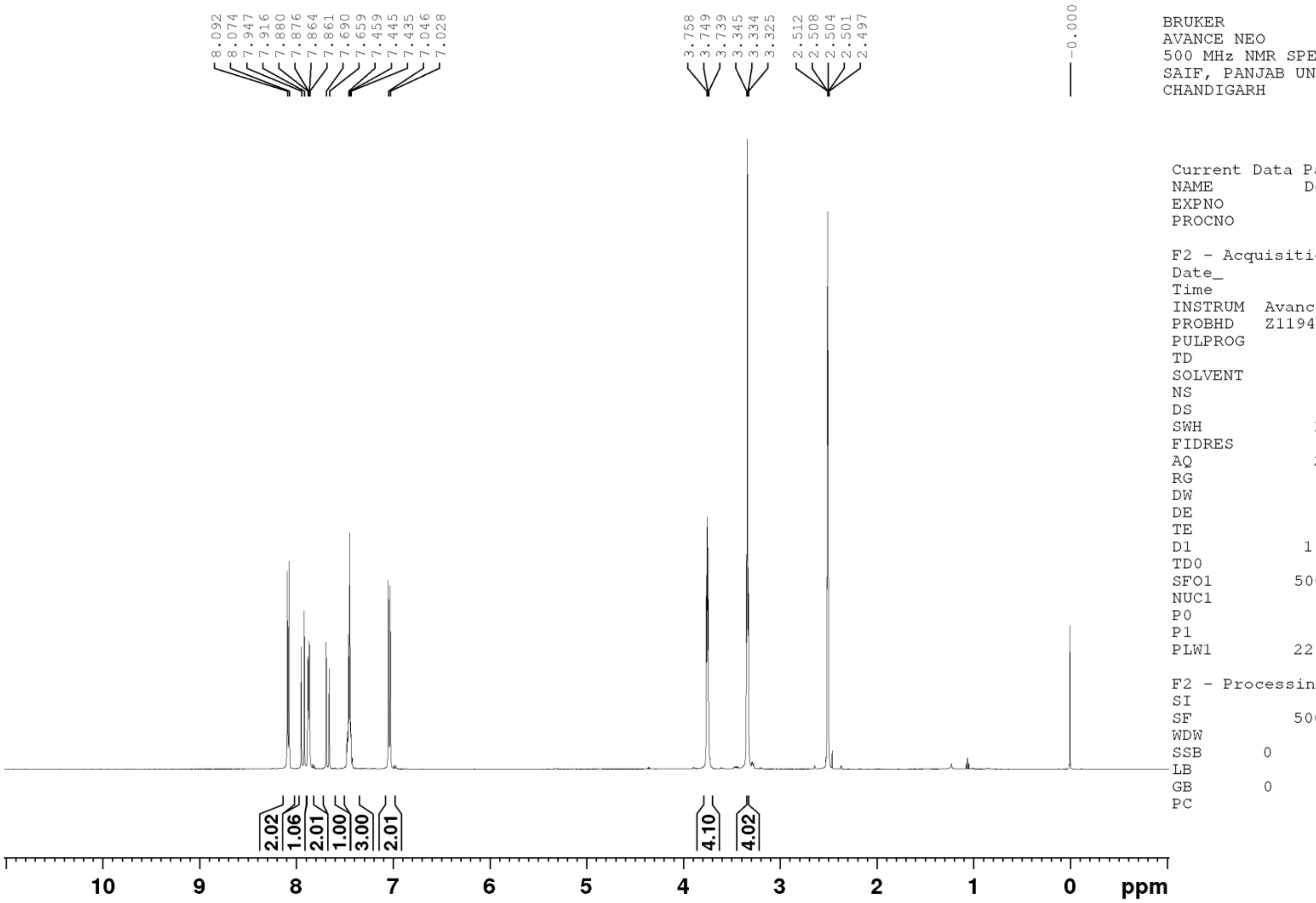

MO-1
1H_8scan DMSO {D:\Spectra} nmr 6

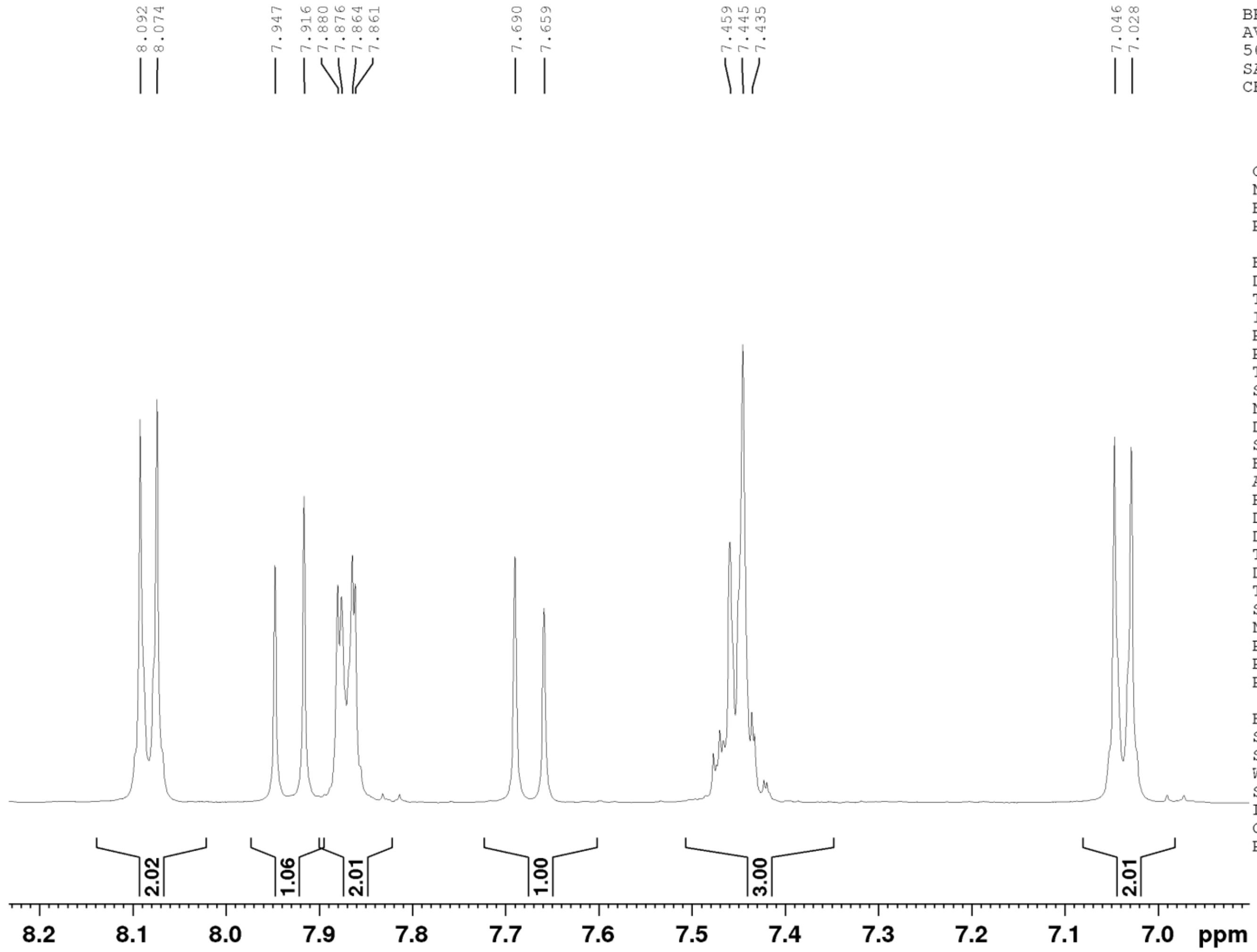

BRUKER
AVANCE NEO
500 MHz NMR SPE
SAIF, PANJAB UN
CHANDIGARH

Current Data Pa
NAME D
EXPNO
PROCNO

F2 - Acquisitio
Date_
Time
INSTRUM Avanc
PROBHD Z1194
PULPROG
TD
SOLVENT
NS
DS
SWH
FIDRES
AQ
RG
DW
DE
TE
D1 1
TD0
SFO1 50
NUC1
P0
P1
PLW1 22

F2 - Processin
SI
SF 50
WDW
SSB 0
LB
GB 0
PC

MO-1
1H_8scan DMSO {D:\Spectra} nmr 6

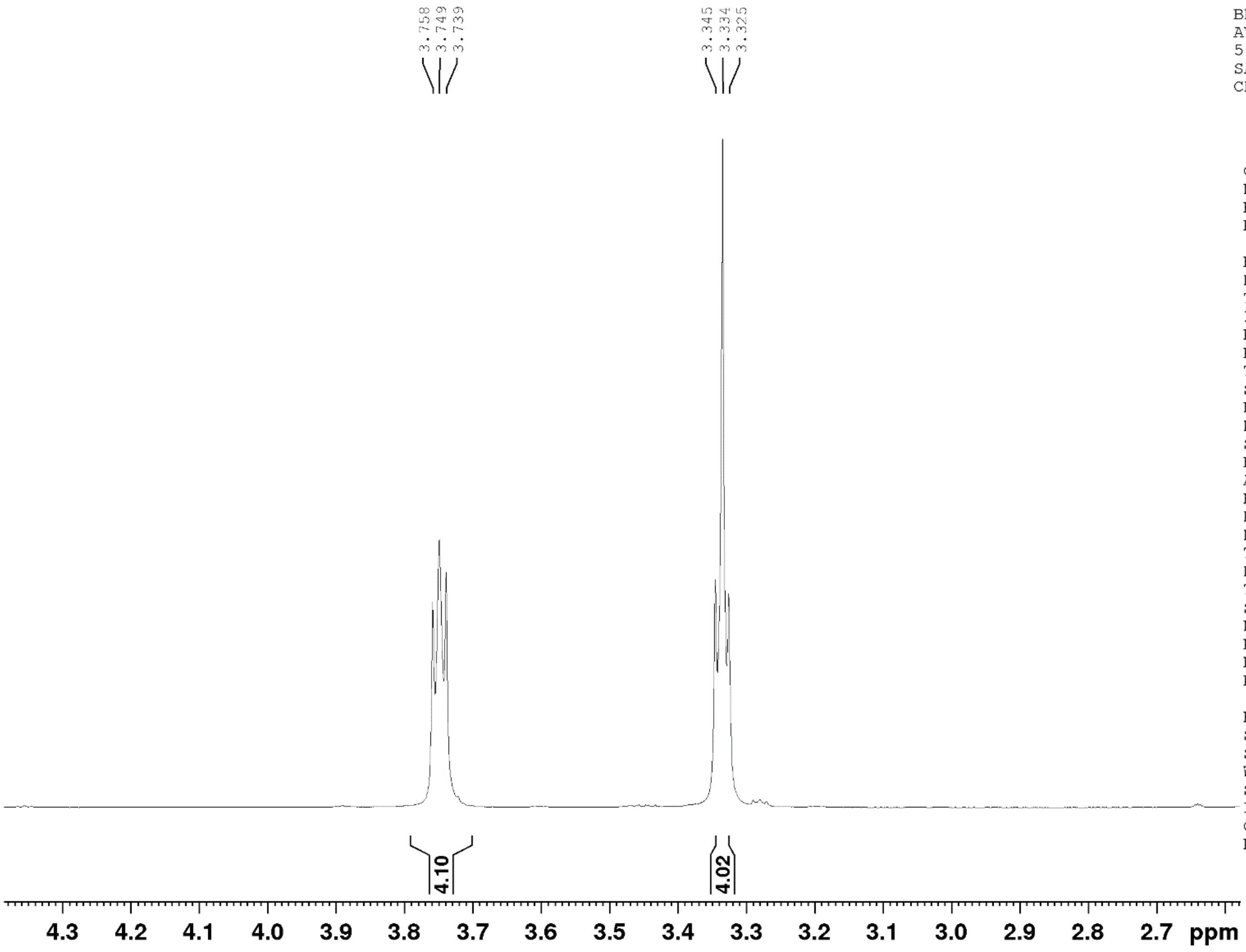

BRUKER
AVANCE NEO
500 MHz NMR SPE
SAIF, PANJAB UN
CHANDIGARH

Current Data P
NAME D
EXPNO
PROCNO

F2 - Acquisiti
Date_
Time
INSTRUM Avanc
PROBHD Z1194
PULPROG
TD
SOLVENT
NS
DS
SWH
FIDRES
AQ
RG
DW
DE
TE
D1 1
TD0
SFO1 50
NUC1
P0
P1
PLW1 22

F2 - Processin
SI
SF 50
WDW
SSB 0
LB
GB 0
PC

MO-1
C13CPD DMSO {D:\Spectra} nmr 6

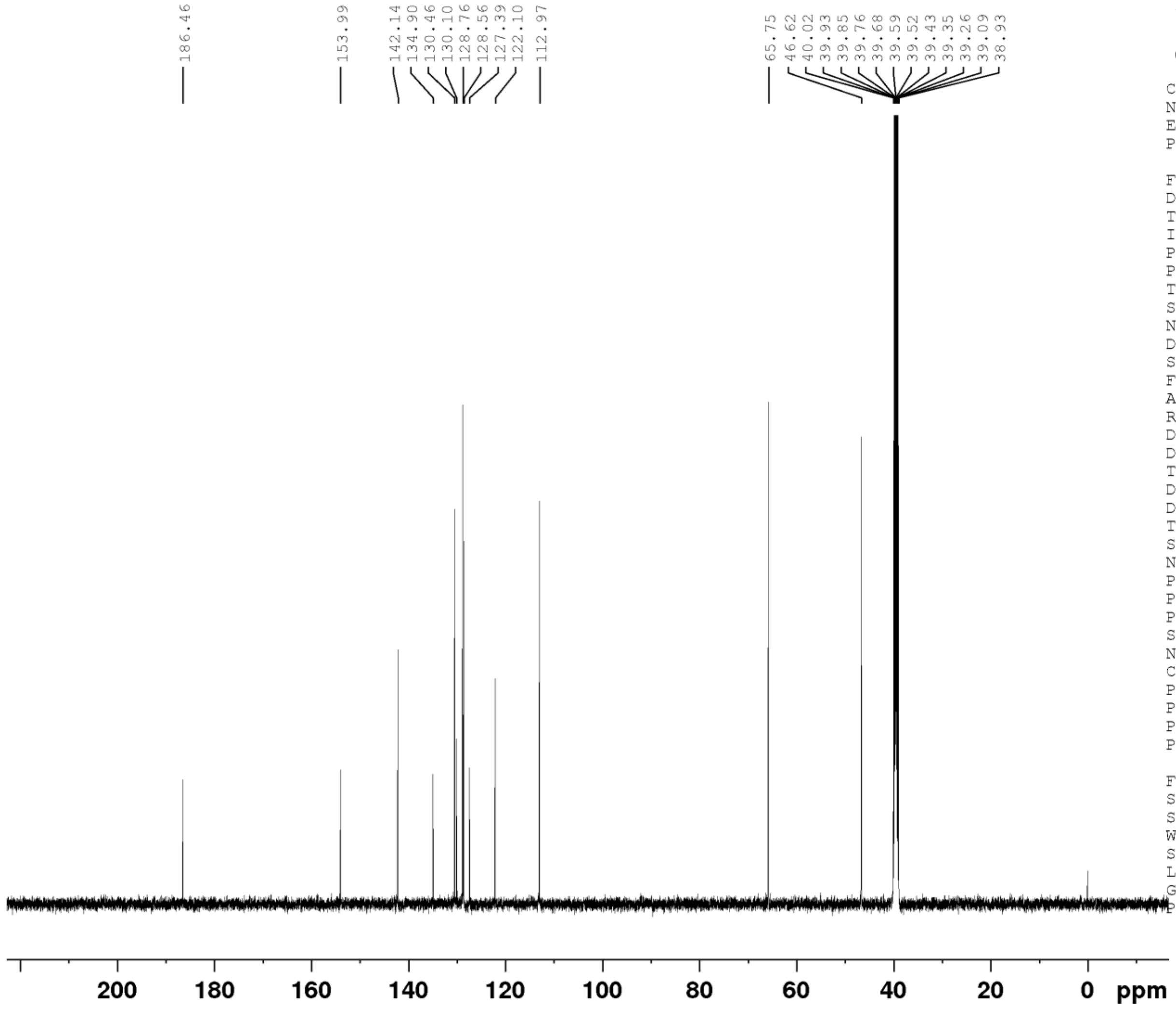

BRUKER
AVANCE NEO
500 MHz NMR SPECT
SAIF, PANJAB UNIV
CHANDIGARH

Current Data Parameters
NAME Dec24-2019
EXPNO 61
PROCNO 1

F2 - Acquisition Parameters
Date_ 20191224
Time 14.42 h
INSTRUM Avance Neo 500
PROBHD Z119470_0333 (
PULPROG zgpg30
TD 65536
SOLVENT DMSO
NS 512
DS 4
SWH 37037.035 Hz
FIDRES 1.130281 Hz
AQ 0.8847360 se
RG 101
DW 13.500 us
DE 6.50 us
TE 298.3 K
D1 2.00000000 se
D11 0.03000000 se
TD0 1
SFO1 125.7804233 MH
NUC1 13C
P0 3.33 us
P1 10.00 us
PLW1 79.56099701 W
SFO2 500.1720007 MH
NUC2 1H
CPDPRG[2 waltz65
PCPD2 80.00 us
PLW2 22.02300072 W
PLW12 0.34411001 W
PLW13 0.17308000 W

F2 - Processing parameters
SI 32768
SF 125.7679190 MH
WDW EM
SSB 0
LB 1.00 Hz
GB 0
PC 1.40

MO-1
C13CPD DMSO {D:\Spectra} nmr 6

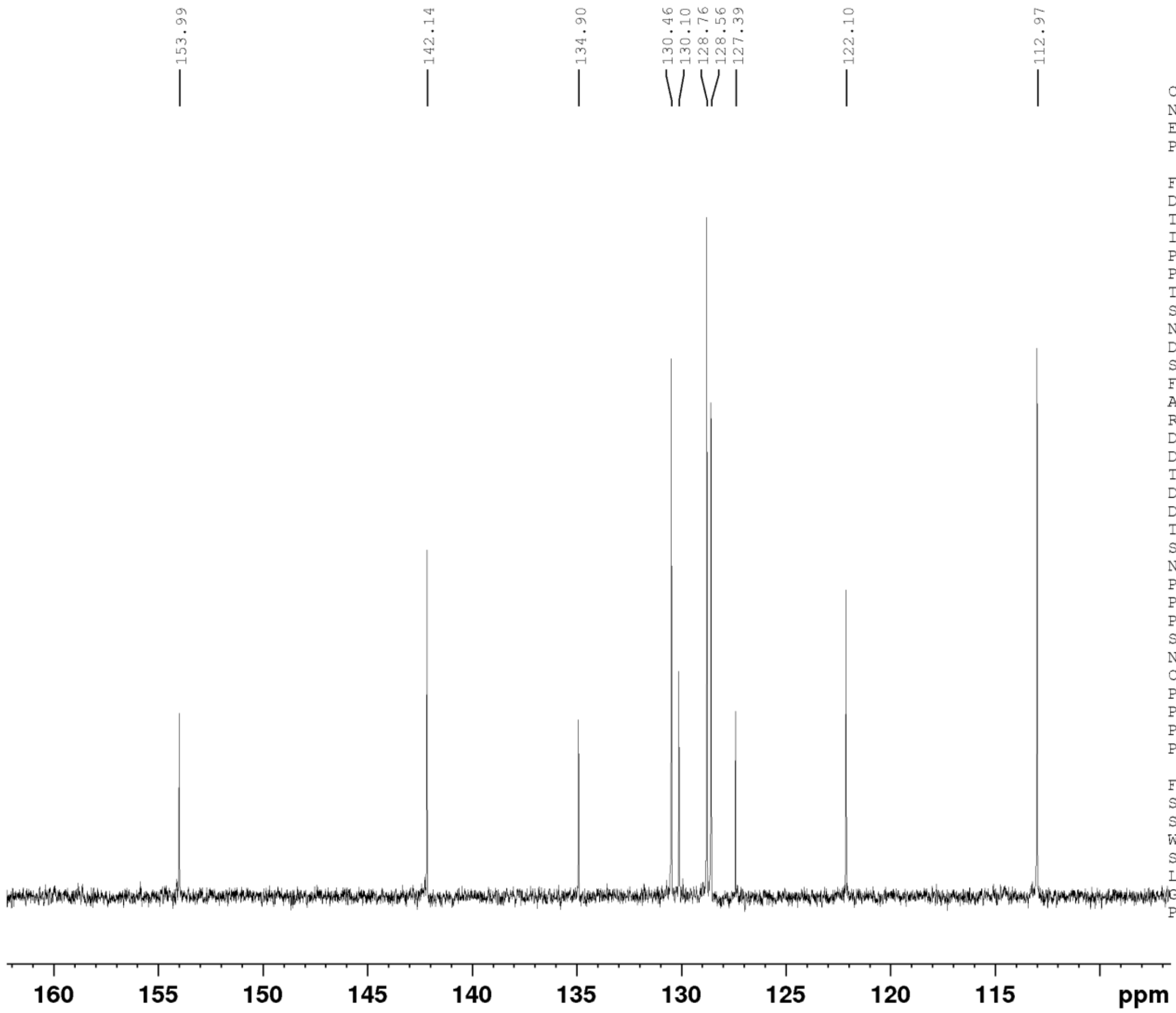

BRUKER
AVANCE NEO
500 MHz NMR SPECT
SAIF, PANJAB UNIV
CHANDIGARH

Current Data Parameters
NAME Dec24-2019
EXPNO 61
PROCNO 1

F2 - Acquisition Paramete
Date_ 20191224
Time 14.42 h
INSTRUM Avance Neo 500
PROBHD Z119470_0333 (
PULPROG zgpg30
TD 65536
SOLVENT DMSO
NS 512
DS 4
SWH 37037.035 Hz
FIDRES 1.130281 Hz
AQ 0.8847360 se
RG 101
DW 13.500 us
DE 6.50 us
TE 298.3 K
D1 2.00000000 se
D11 0.03000000 se
TD0 1
SFO1 125.7804233 MH
NUC1 13C
P0 3.33 us
P1 10.00 us
PLW1 79.56099701 W
SFO2 500.1720007 MH
NUC2 1H
CPDPRG[2 waltz65
PCPD2 80.00 us
PLW2 22.02300072 W
PLW12 0.34411001 W
PLW13 0.17308000 W

F2 - Processing parameters
SI 32768
SF 125.7679190 MH
WDW EM
SSB 0
LB 1.00 Hz
GB 0
PC 1.40

MO-1
C13CPD DMSO {D:\Spectra} nmr 6

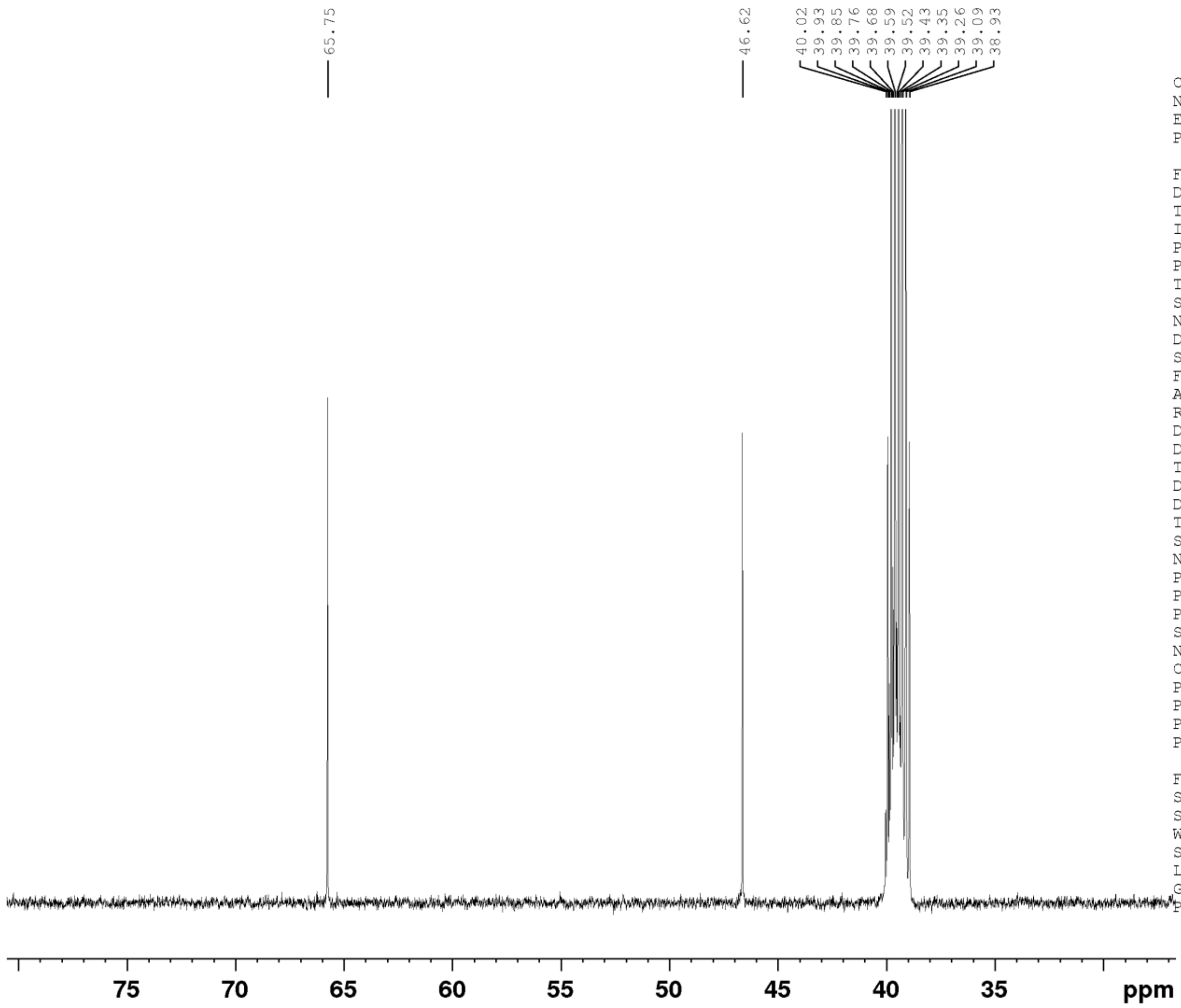

BRUKER
AVANCE NEO
500 MHz NMR SPECT
SAIF, PANJAB UNIV
CHANDIGARH

Current Data Parameters
NAME Dec24-2019
EXPNO 61
PROCNO 1

F2 - Acquisition Parameter

Date_	20191224	
Time	14.42	h
INSTRUM	Avance Neo 500	
PROBHD	Z119470_0333 (	
PULPROG	zgpg30	
TD	65536	
SOLVENT	DMSO	
NS	512	
DS	4	
SWH	37037.035	Hz
FIDRES	1.130281	Hz
AQ	0.8847360	se
RG	101	
DW	13.500	us
DE	6.50	us
TE	298.3	K
D1	2.00000000	se
D11	0.03000000	se
TD0	1	
SFO1	125.7804233	MH
NUC1	13C	
P0	3.33	us
P1	10.00	us
PLW1	79.56099701	W
SFO2	500.1720007	MH
NUC2	1H	
CPDPRG[2	waltz65	
PCPD2	80.00	us
PLW2	22.02300072	W
PLW12	0.34411001	W
PLW13	0.17308000	W

F2 - Processing parameters

SI	32768	
SF	125.7679190	MH
WDW	EM	
SSB	0	
LB	1.00	Hz
GB	0	
PC	1.40	

MO-3
1H_8scan DMSO {D:\Spectra} nmr 7

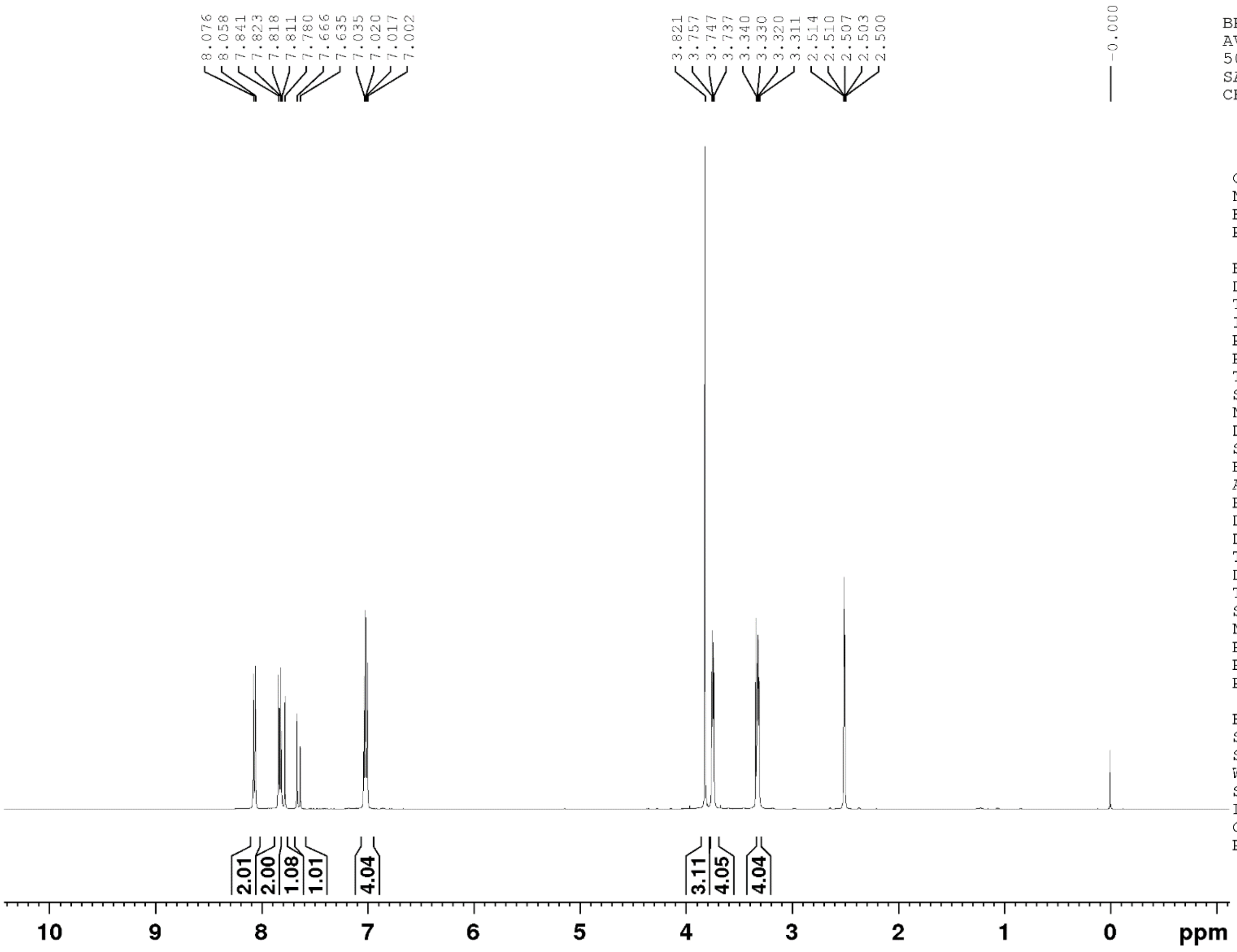

BRUKER
AVANCE NEO
500 MHz NMR SPE
SAIF, PANJAB UN
CHANDIGARH

Current Data P
NAME D
EXPNO
PROCNO

F2 - Acquisiti
Date_
Time
INSTRUM Avanc
PROBHD Z1194
PULPROG
TD
SOLVENT
NS
DS
SWH
FIDRES
AQ
RG
DW
DE
TE
D1 1
TD0
SFO1 50
NUC1
P0
P1
PLW1 22

F2 - Processin
SI
SF 50
WDW
SSB 0
LB
GB 0
PC

MO-3
1H_8scan DMSO {D:\Spectra} nmr 7

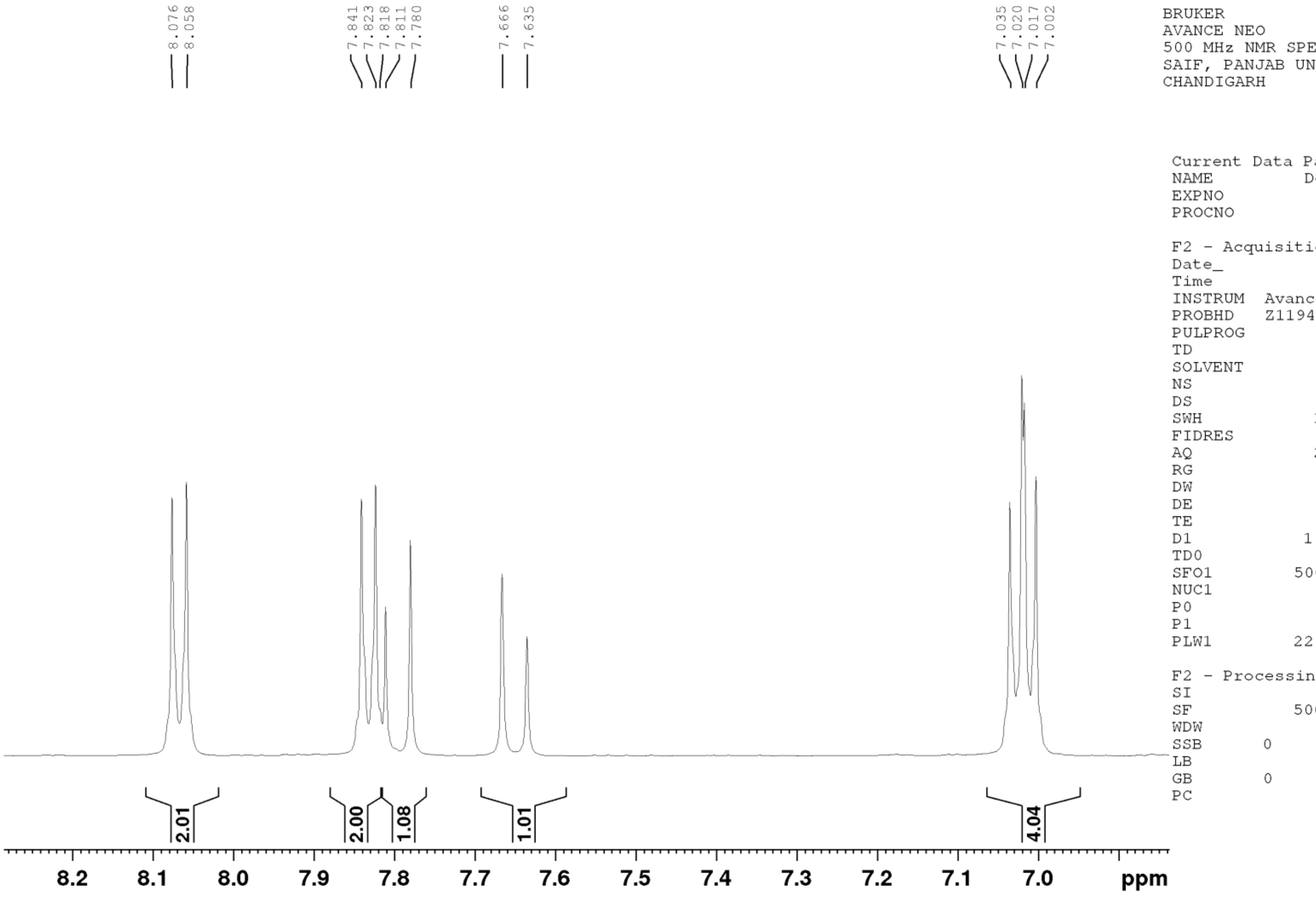

MO-3
1H_8scan DMSO {D:\Spectra} nmr 7

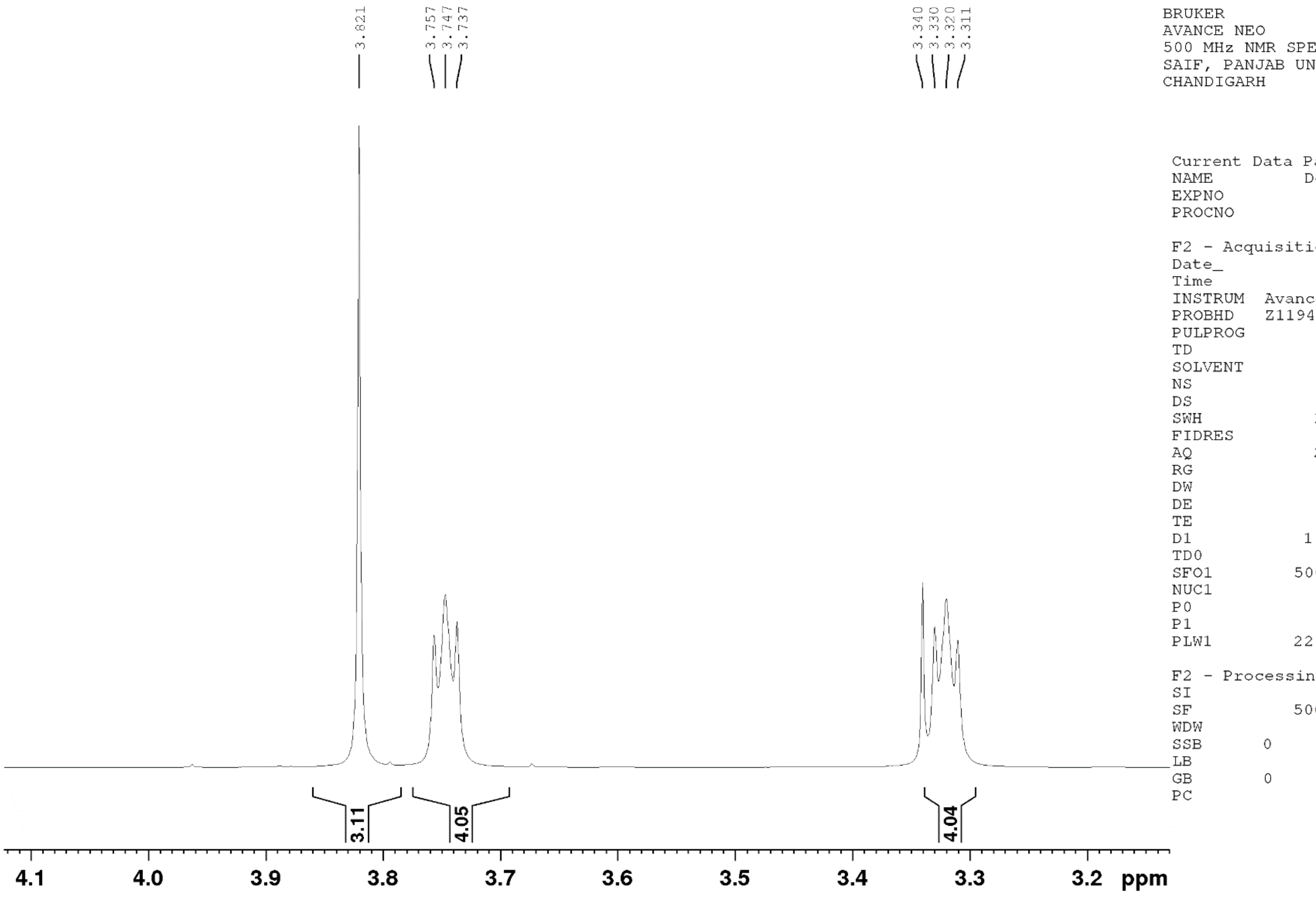

MO-3
C13CPD DMSO {D:\Spectra} nmr 7

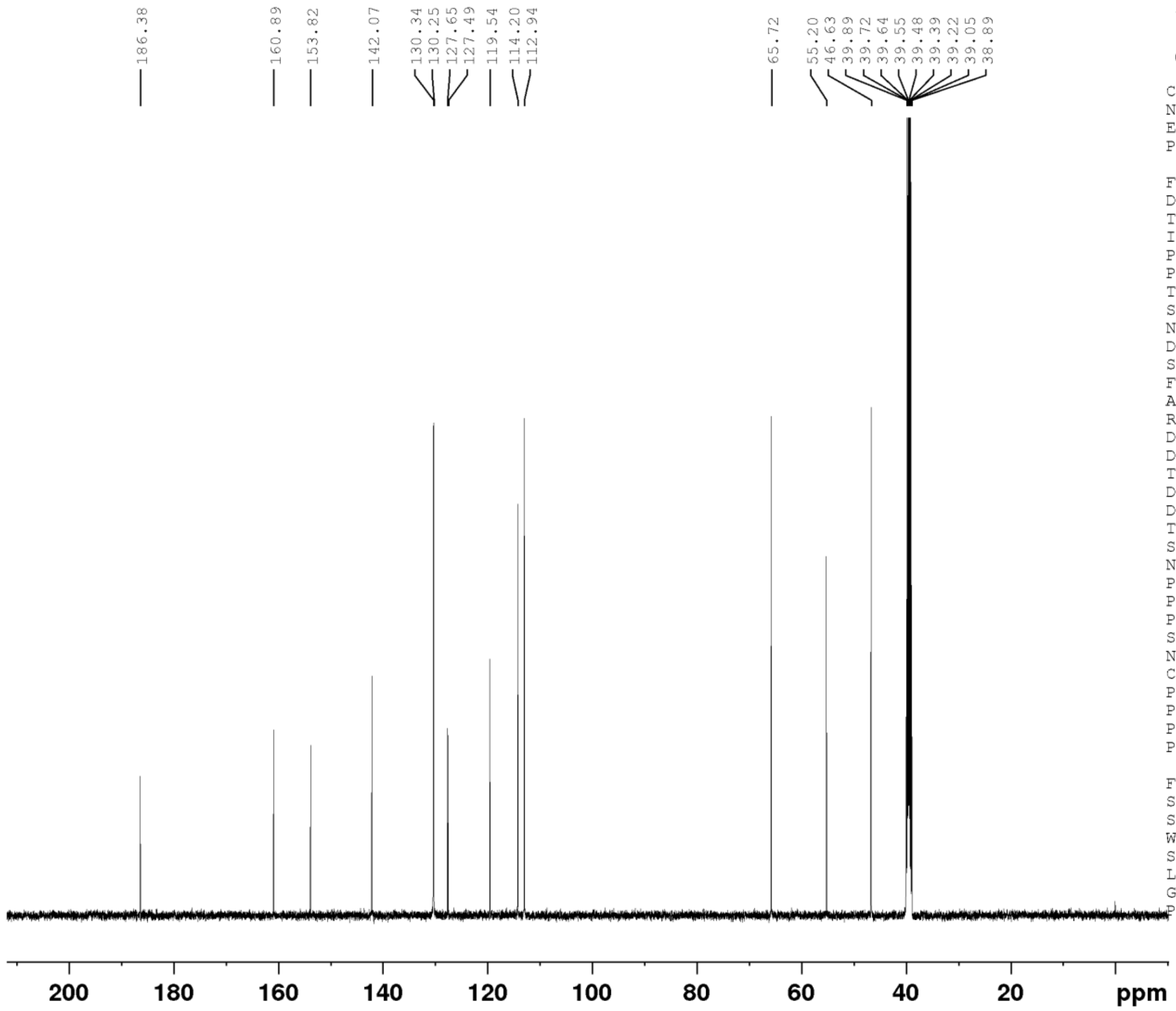

BRUKER
AVANCE NEO
500 MHz NMR SPECT
SAIF, PANJAB UNIV
CHANDIGARH

Current Data Parameters
NAME Dec24-2019
EXPNO 71
PROCNO 1

F2 - Acquisition Parameter
Date_ 20191224
Time 15.08 h
INSTRUM Avance Neo 500
PROBHD Z119470_0333 (
PULPROG zgpg30
TD 65536
SOLVENT DMSO
NS 512
DS 4
SWH 37037.035 Hz
FIDRES 1.130281 Hz
AQ 0.8847360 se
RG 101
DW 13.500 us
DE 6.50 us
TE 298.3 K
D1 2.00000000 sec
D11 0.03000000 sec
TD0 1
SFO1 125.7804233 MH
NUC1 13C
P0 3.33 us
P1 10.00 us
PLW1 79.56099701 W
SFO2 500.1720007 MH
NUC2 1H
CPDPRG[2 waltz65
PCPD2 80.00 us
PLW2 22.02300072 W
PLW12 0.34411001 W
PLW13 0.17308000 W

F2 - Processing parameters
SI 32768
SF 125.7679241 MH
WDW EM
SSB 0
LB 1.00 Hz
GB 0
PC 1.40

MO-3
C13CPD DMSO {D:\Spectra} nmr 7

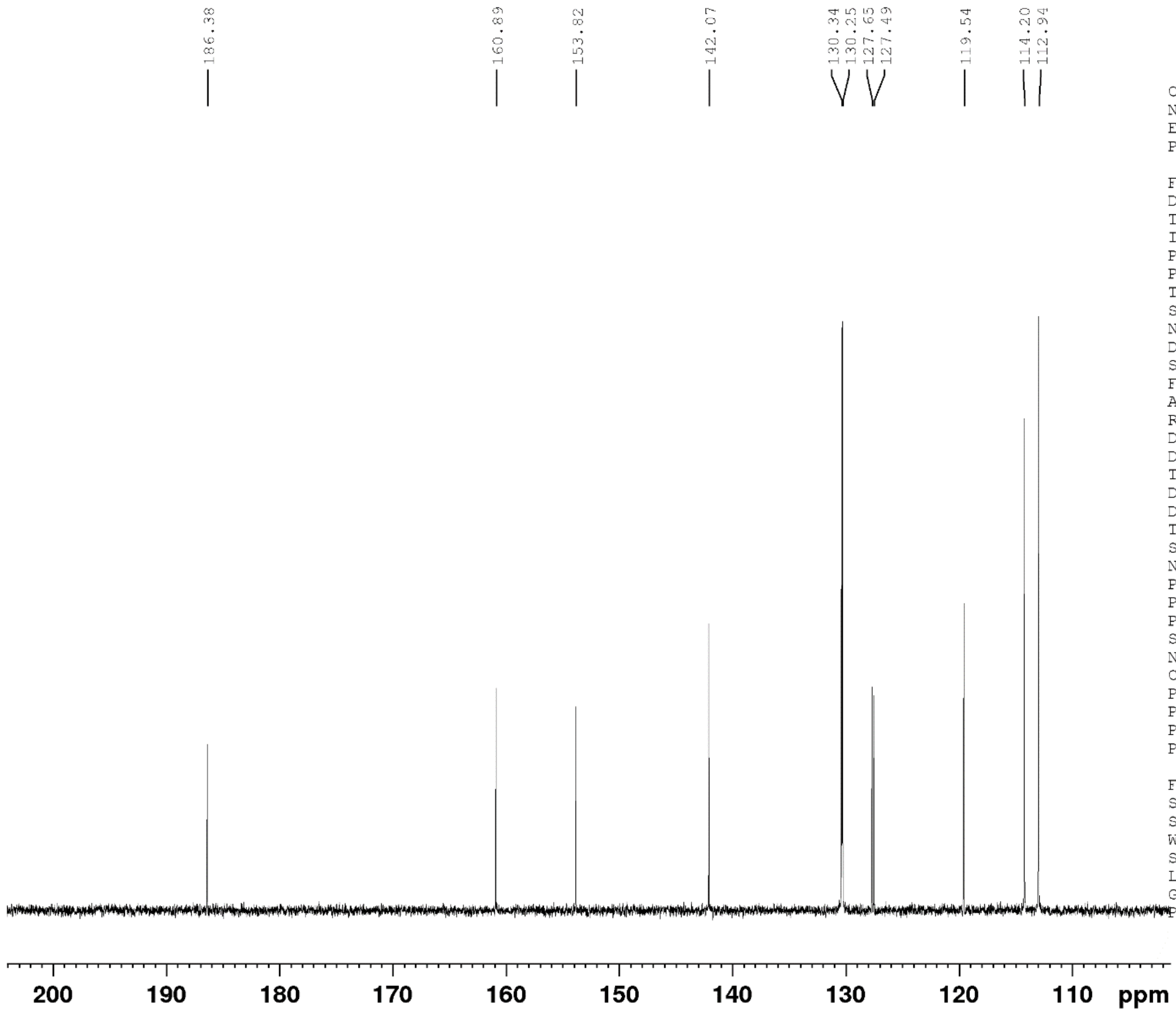

BRUKER
AVANCE NEO
500 MHz NMR SPECT
SAIF, PANJAB UNIV
CHANDIGARH

Current Data Parameters
NAME Dec24-2019
EXPNO 71
PROCNO 1

F2 - Acquisition Parameters
Date_ 20191224
Time 15.08 h
INSTRUM Avance Neo 500
PROBHD Z119470_0333 (
PULPROG zgpg30
TD 65536
SOLVENT DMSO
NS 512
DS 4
SWH 37037.035 Hz
FIDRES 1.130281 Hz
AQ 0.8847360 se
RG 101
DW 13.500 us
DE 6.50 us
TE 298.3 K
D1 2.00000000 se
D11 0.03000000 se
TD0 1
SFO1 125.7804233 MH
NUC1 13C
P0 3.33 us
P1 10.00 us
PLW1 79.56099701 W
SFO2 500.1720007 MH
NUC2 1H
CPDPRG[2 waltz65
PCPD2 80.00 us
PLW2 22.02300072 W
PLW12 0.34411001 W
PLW13 0.17308000 W

F2 - Processing parameters
SI 32768
SF 125.7679241 MH
WDW EM
SSB 0
LB 1.00 Hz
GB 0
PC 1.40

MO-3
C13CPD DMSO {D:\Spectra} nmr 7

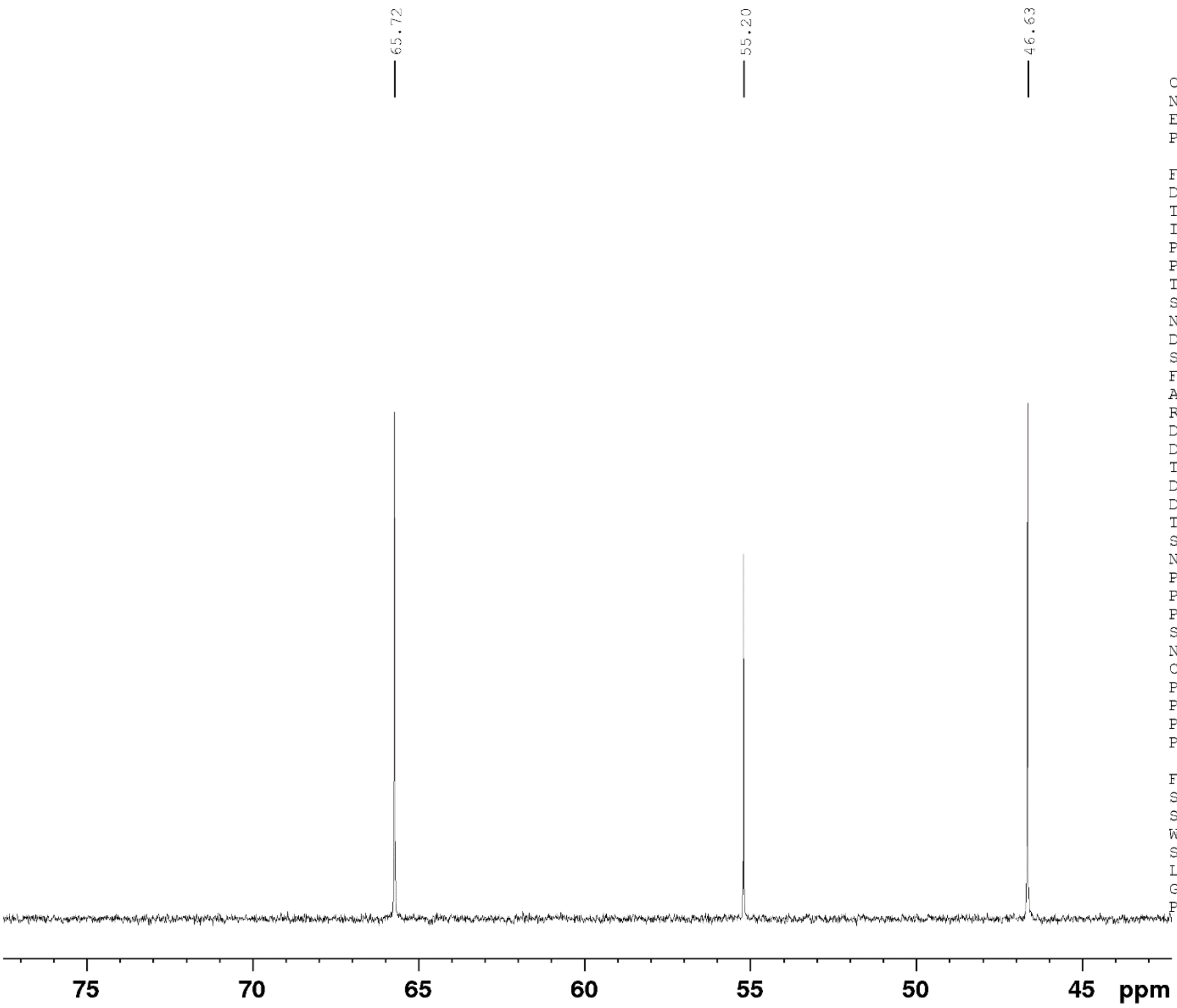

BRUKER
AVANCE NEO
500 MHz NMR SPECT
SAIF, PANJAB UNIV
CHANDIGARH

Current Data Parameters
NAME Dec24-2019
EXPNO 71
PROCNO 1

F2 - Acquisition Parameter
Date_ 20191224
Time 15.08 h
INSTRUM Avance Neo 500
PROBHD Z119470_0333 (
PULPROG zgpg30
TD 65536
SOLVENT DMSO
NS 512
DS 4
SWH 37037.035 Hz
FIDRES 1.130281 Hz
AQ 0.8847360 se
RG 101
DW 13.500 us
DE 6.50 us
TE 298.3 K
D1 2.00000000 se
D11 0.03000000 se
TD0 1
SFO1 125.7804233 MH
NUC1 13C
P0 3.33 us
P1 10.00 us
PLW1 79.56099701 W
SFO2 500.1720007 MH
NUC2 1H
CPDPRG[2 waltz65
PCPD2 80.00 us
PLW2 22.02300072 W
PLW12 0.34411001 W
PLW13 0.17308000 W

F2 - Processing parameters
SI 32768
SF 125.7679241 MH
WDW EM
SSB 0
LB 1.00 Hz
GB 0
PC 1.40

MO-4
1H_8scan DMSO {D:\Spectra} nmr 8

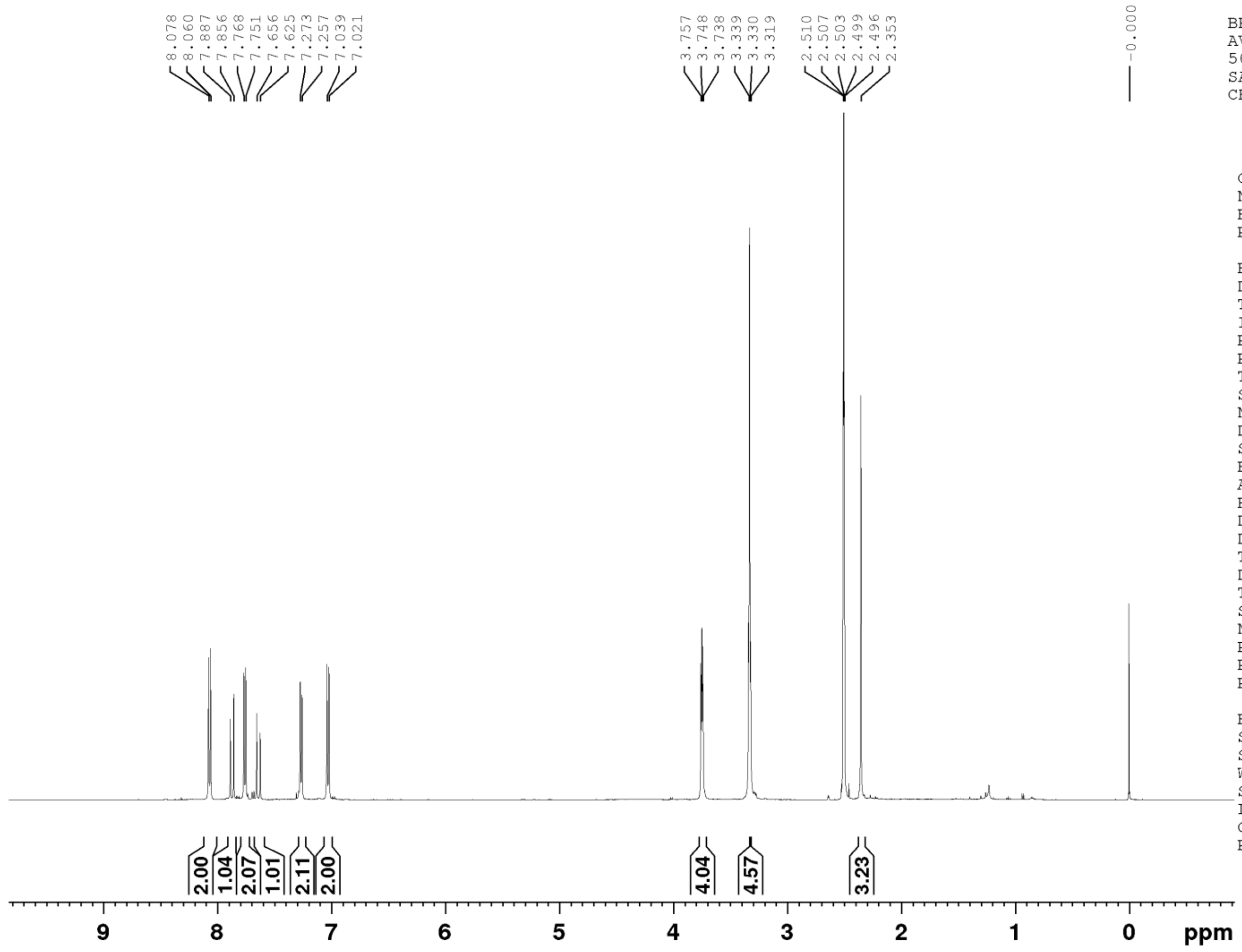

BRUKER
AVANCE NEO
500 MHz NMR SPE
SAIF, PANJAB UN
CHANDIGARH

Current Data Pa
NAME D
EXPNO
PROCNO

F2 - Acquisiti
Date_
Time
INSTRUM Avanc
PROBHD Z1194
PULPROG
TD
SOLVENT
NS
DS
SWH
FIDRES
AQ
RG
DW
DE
TE
D1 1
TD0
SFO1 50
NUC1
P0
P1
PLW1 22

F2 - Processin
SI
SF 50
WDW
SSB 0
LB
GB 0
PC

MO-4
1H_8scan DMSO {D:\Spectra} nmr 8

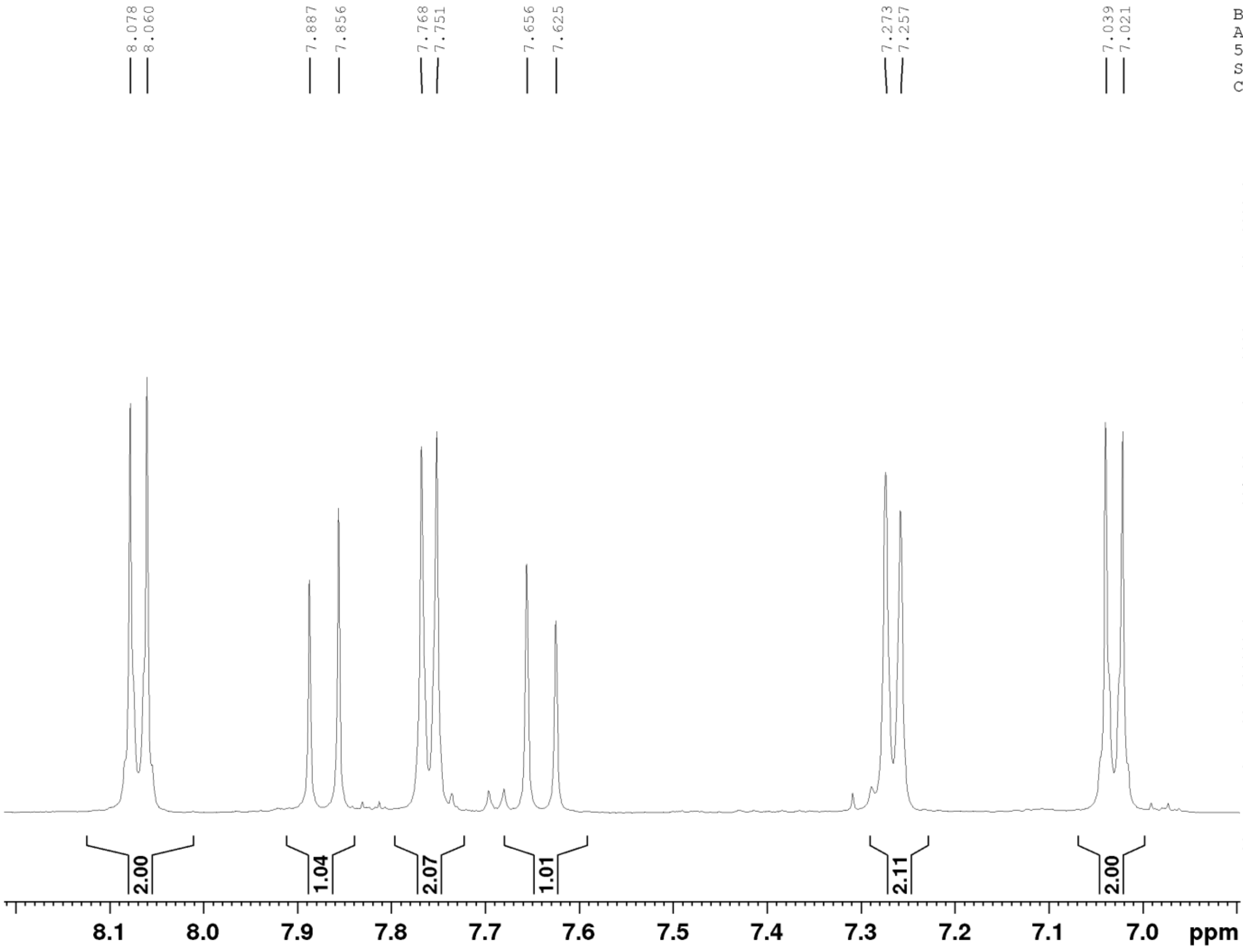

BRUKER
AVANCE NEO
500 MHz NMR SPE
SAIF, PANJAB UN
CHANDIGARH

Current Data Pa
NAME D
EXPNO
PROCNO

F2 - Acquisiti
Date_
Time
INSTRUM Avanc
PROBHD Z1194
PULPROG
TD
SOLVENT
NS
DS
SWH
FIDRES
AQ
RG
DW
DE
TE
D1 1
TD0
SFO1 50
NUC1
P0
P1
PLW1 22

F2 - Processin
SI
SF 50
WDW
SSB 0
LB
GB 0
PC

MO-4
1H_8scan DMSO {D:\Spectra} nmr 8

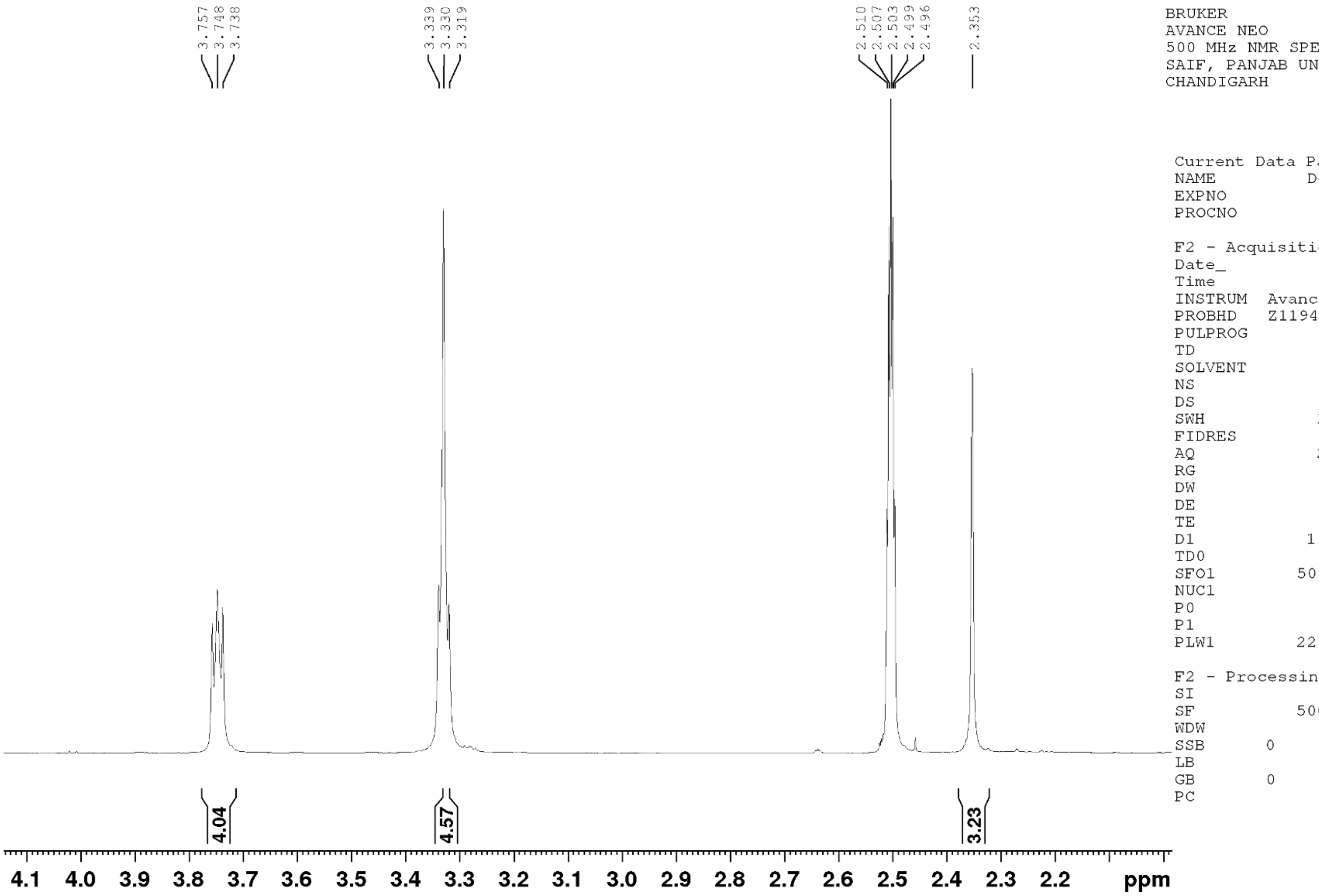

MO-4
C13CPD DMSO {D:\Spectra} nmr 8

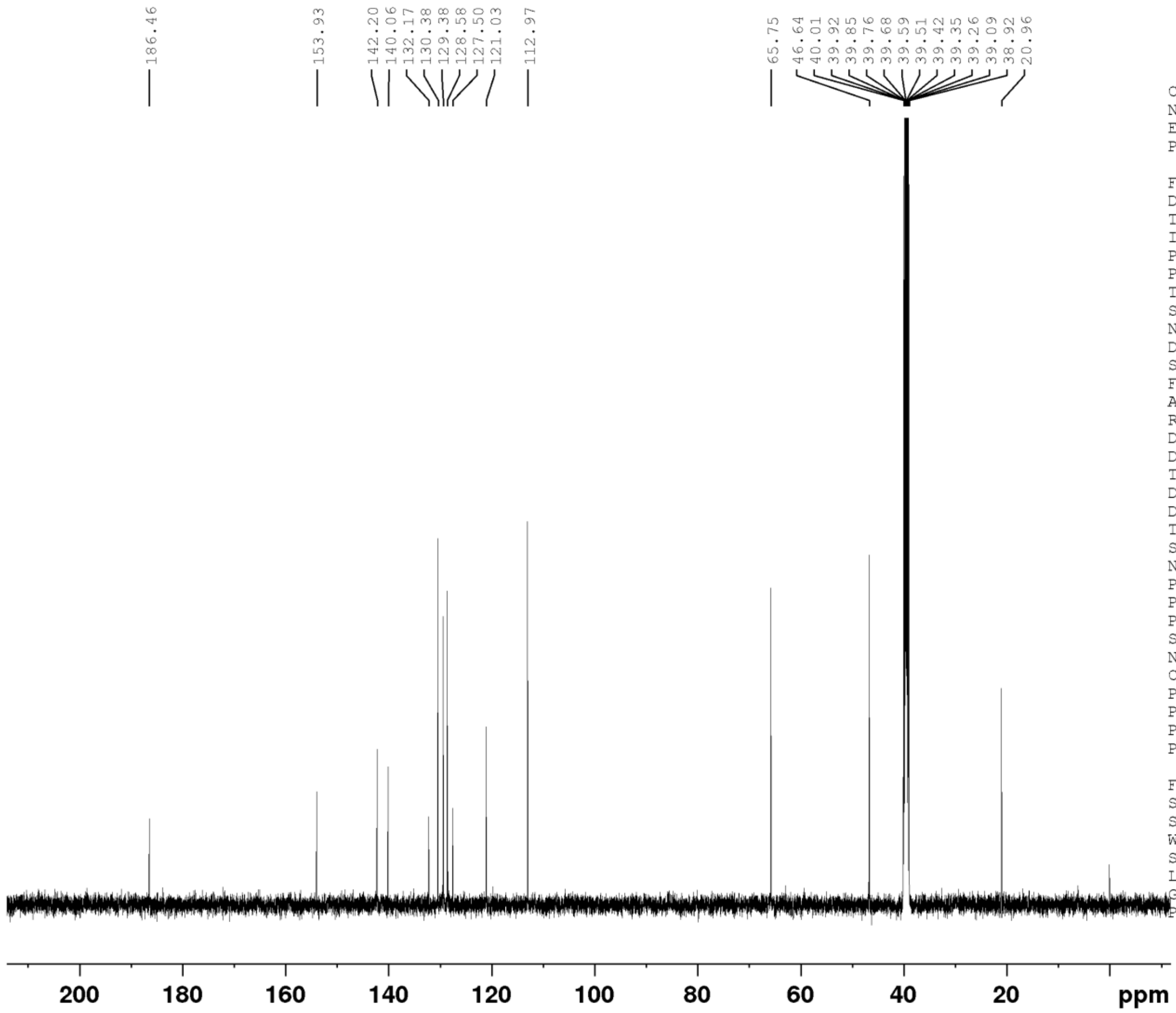

BRUKER
AVANCE NEO
500 MHz NMR SPECT
SAIF, PANJAB UNIV
CHANDIGARH

Current Data Parameters
NAME Dec24-2019
EXPNO 81
PROCNO 1

F2 - Acquisition Parameters
Date_ 20191224
Time 15.39 h
INSTRUM Avance Neo 500
PROBHD Z119470_0333 (
PULPROG zgpg30
TD 65536
SOLVENT DMSO
NS 512
DS 4
SWH 37037.035 Hz
FIDRES 1.130281 Hz
AQ 0.8847360 se
RG 101
DW 13.500 us
DE 6.50 us
TE 298.4 K
D1 2.00000000 sec
D11 0.03000000 sec
TD0 1
SFO1 125.7804233 MHz
NUC1 13C
P0 3.33 us
P1 10.00 us
PLW1 79.56099701 W
SFO2 500.1720007 MHz
NUC2 1H
CPDPRG[2 waltz65
PCPD2 80.00 us
PLW2 22.02300072 W
PLW12 0.34411001 W
PLW13 0.17308000 W

F2 - Processing parameters
SI 32768
SF 125.7679193 MHz
WDW EM
SSB 0
LB 1.00 Hz
GB 0
PC 1.40

MO-4
C13CPD DMSO {D:\Spectra} nmr 8

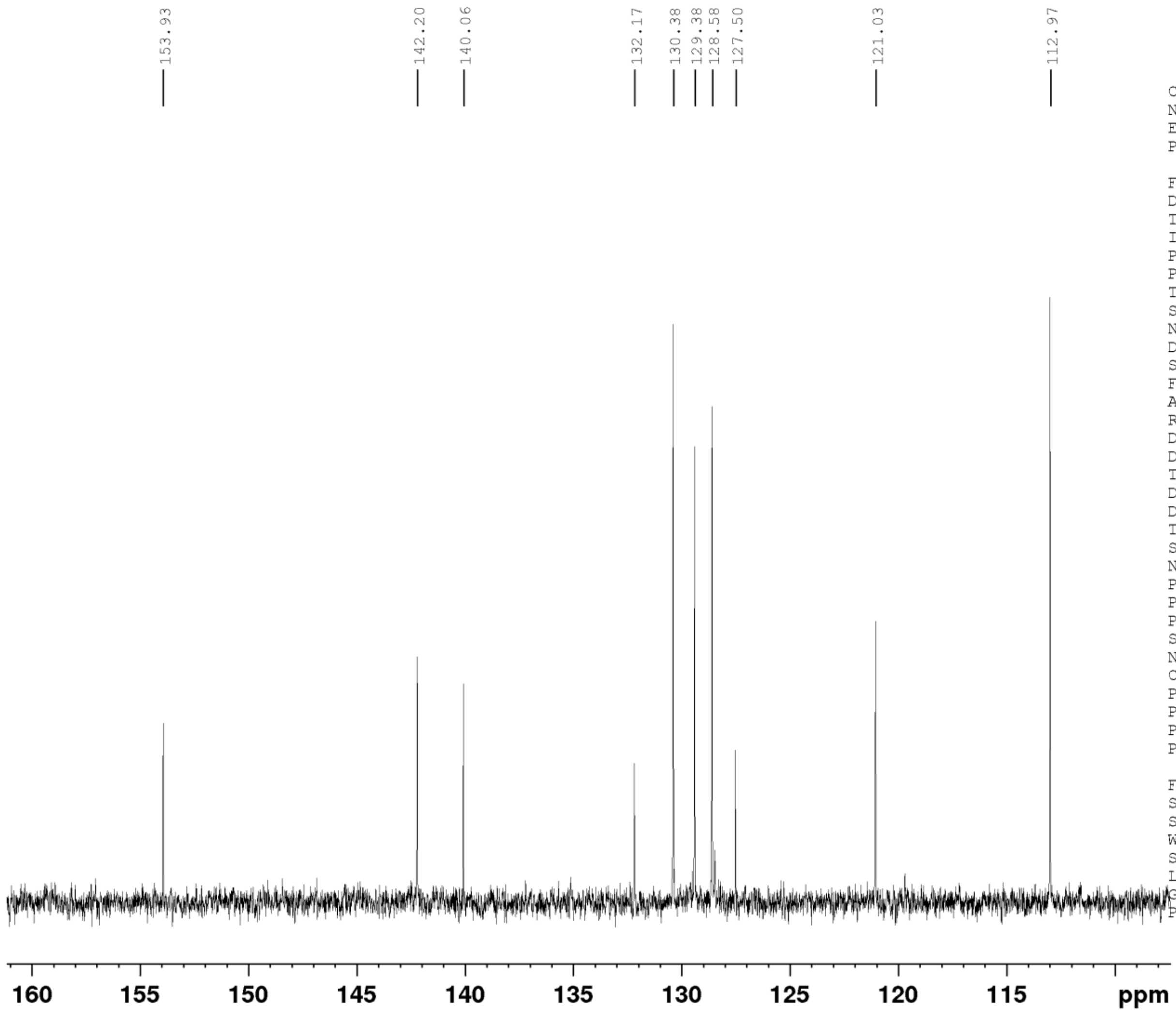

BRUKER
AVANCE NEO
500 MHz NMR SPECT
SAIF, PANJAB UNIV
CHANDIGARH

Current Data Parameters
NAME Dec24-2019
EXPNO 81
PROCNO 1

F2 - Acquisition Parameter
Date_ 20191224
Time 15.39 h
INSTRUM Avance Neo 500
PROBHD Z119470_0333 (
PULPROG zgpg30
TD 65536
SOLVENT DMSO
NS 512
DS 4
SWH 37037.035 Hz
FIDRES 1.130281 Hz
AQ 0.8847360 se
RG 101
DW 13.500 us
DE 6.50 us
TE 298.4 K
D1 2.00000000 se
D11 0.03000000 se
TD0 1
SFO1 125.7804233 MH
NUC1 13C
P0 3.33 us
P1 10.00 us
PLW1 79.56099701 W
SFO2 500.1720007 MH
NUC2 1H
CPDPRG[2 waltz65
PCPD2 80.00 us
PLW2 22.02300072 W
PLW12 0.34411001 W
PLW13 0.17308000 W

F2 - Processing parameters
SI 32768
SF 125.7679193 MH
WDW EM
SSB 0
LB 1.00 Hz
GB 0
PC 1.40

MO-4
C13CPD DMSO {D:\Spectra} nmr 8

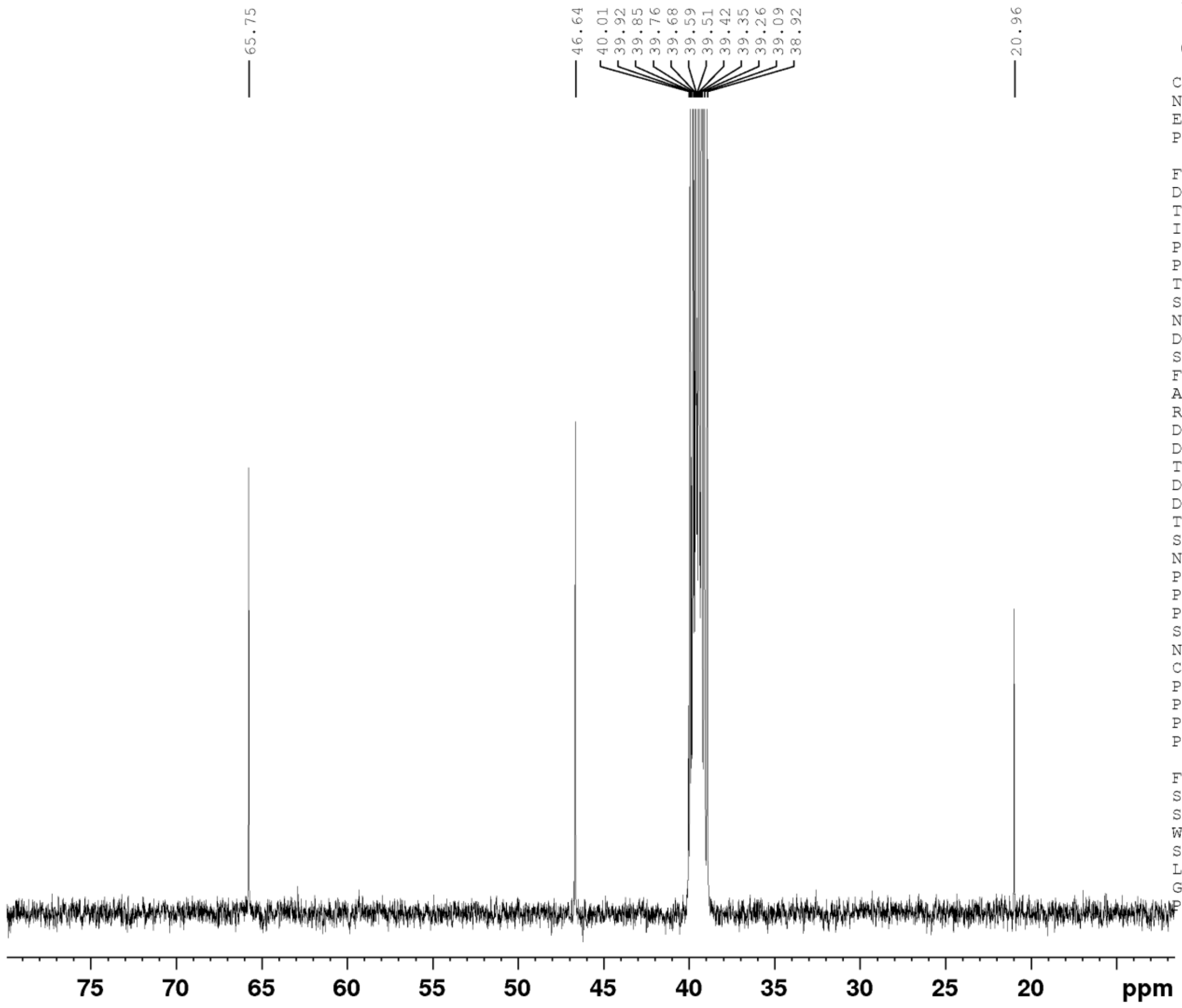

BRUKER
AVANCE NEO
500 MHz NMR SPECT
SAIF, PANJAB UNIV
CHANDIGARH

Current Data Parameters
NAME Dec24-2019
EXPNO 81
PROCNO 1

F2 - Acquisition Parameters
Date_ 20191224
Time 15.39 h
INSTRUM Avance Neo 500
PROBHD Z119470_0333 (
PULPROG zgpg30
TD 65536
SOLVENT DMSO
NS 512
DS 4
SWH 37037.035 Hz
FIDRES 1.130281 Hz
AQ 0.8847360 se
RG 101
DW 13.500 us
DE 6.50 us
TE 298.4 K
D1 2.00000000 sec
D11 0.03000000 sec
TD0 1
SFO1 125.7804233 MH
NUC1 13C
P0 3.33 us
P1 10.00 us
PLW1 79.56099701 W
SFO2 500.1720007 MH
NUC2 1H
CPDPRG[2 waltz65
PCPD2 80.00 us
PLW2 22.02300072 W
PLW12 0.34411001 W
PLW13 0.17308000 W

F2 - Processing parameters
SI 32768
SF 125.7679193 MH
WDW EM
SSB 0
LB 1.00 Hz
GB 0
PC 1.40

MO-5
1H_8scan DMSO {D:\Spectra} nmr 9

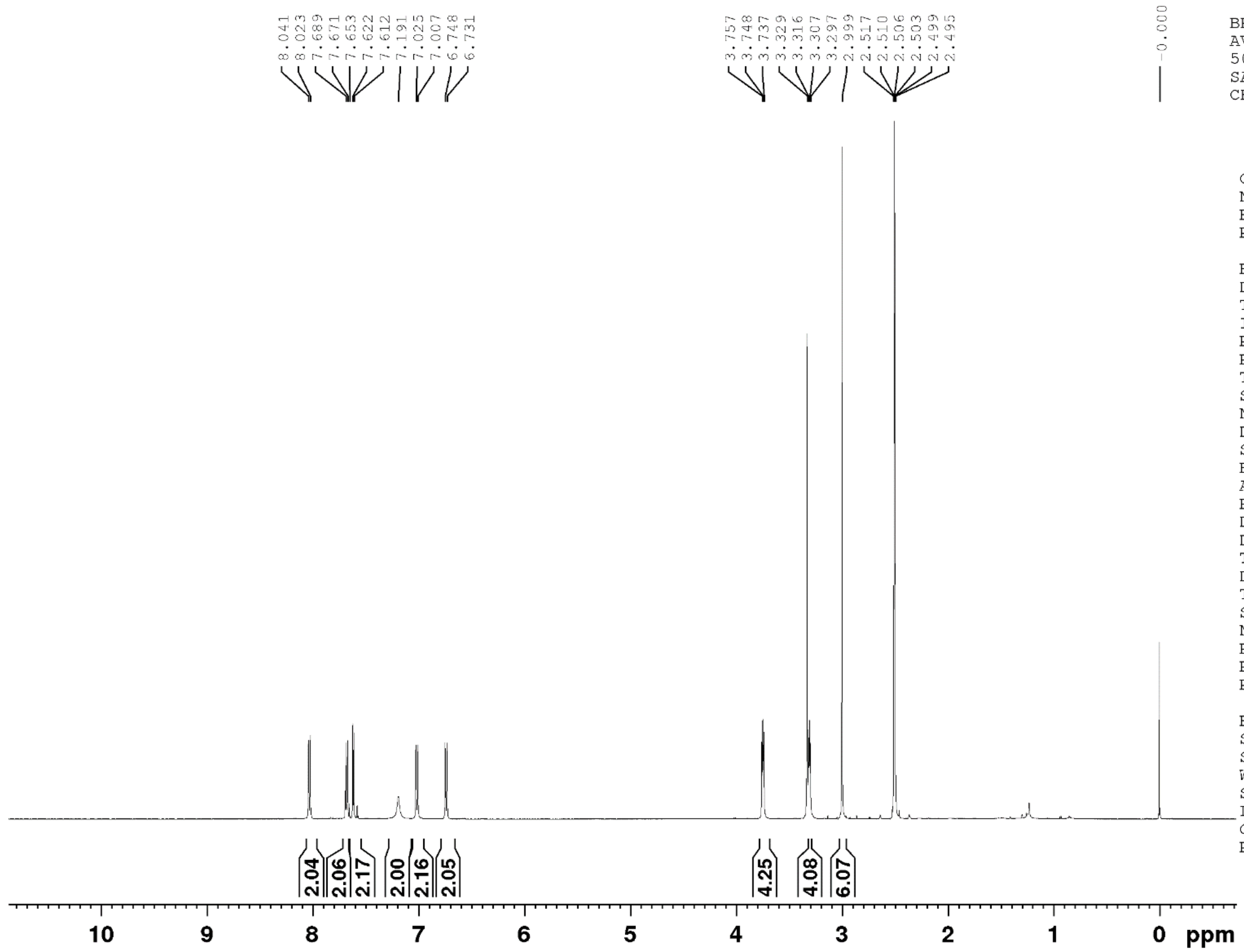

BRUKER
AVANCE NEO
500 MHz NMR SPE
SAIF, PANJAB UN
CHANDIGARH

Current Data P
NAME D
EXPNO
PROCNO

F2 - Acquisiti
Date_
Time
INSTRUM Avanc
PROBHD Z1194
PULPROG
TD
SOLVENT
NS
DS
SWH
FIDRES
AQ
RG
DW
DE
TE
D1 1
TD0
SFO1 50
NUC1
P0
P1
PLW1 22

F2 - Processin
SI
SF 50
WDW
SSB 0
LB
GB 0
PC

MO-5
1H_8scan DMSO {D:\Spectra} nmr 9

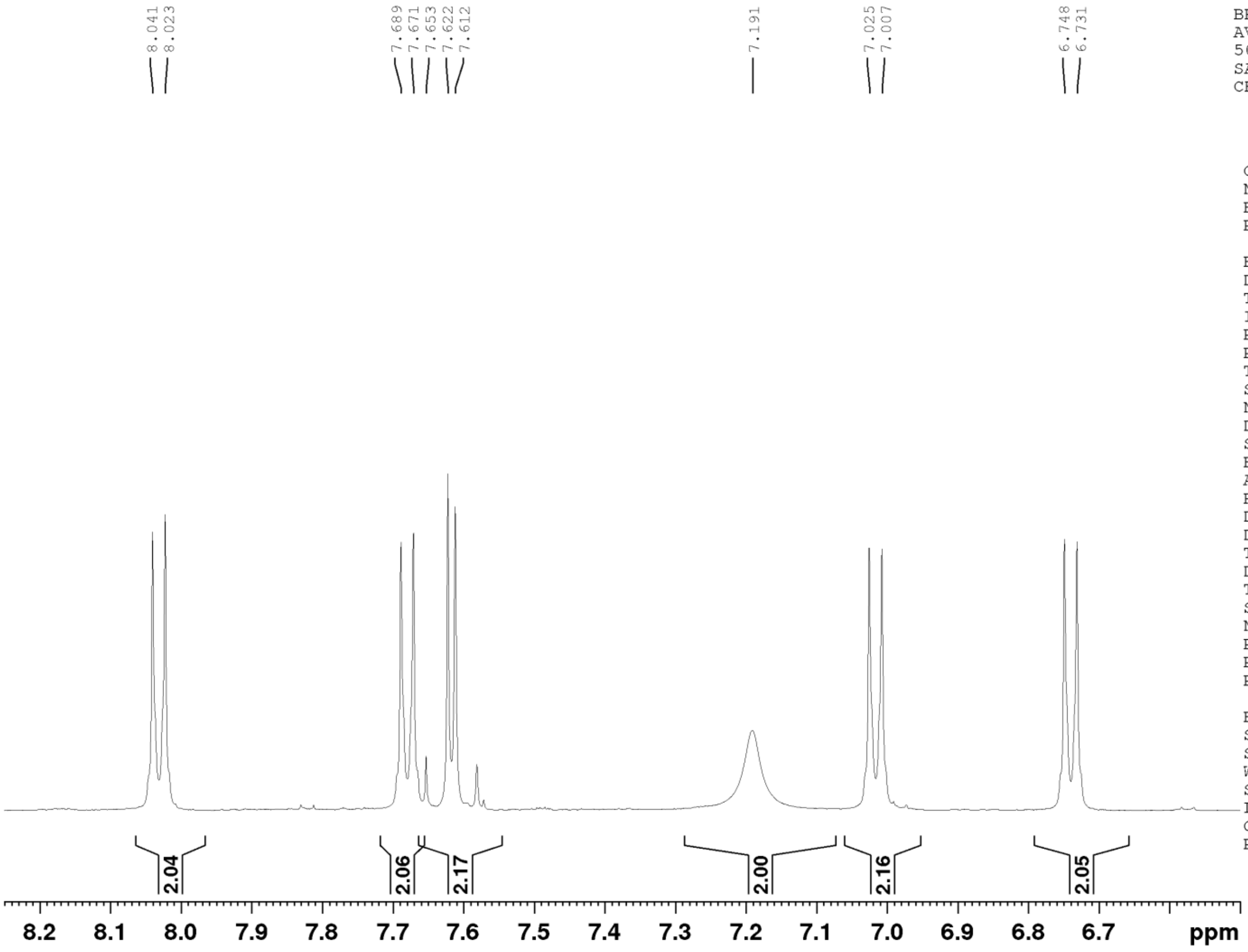

BRUKER
AVANCE NEO
500 MHz NMR SPE
SAIF, PANJAB UN
CHANDIGARH

Current Data P
NAME D
EXPNO
PROCNO

F2 - Acquisiti
Date_
Time
INSTRUM Avanc
PROBHD Z1194
PULPROG
TD
SOLVENT
NS
DS
SWH
FIDRES
AQ
RG
DW
DE
TE
D1 1
TD0
SFO1 50
NUC1
P0
P1
PLW1 22

F2 - Processin
SI
SF 50
WDW
SSB 0
LB
GB 0
PC

MO-5
1H_8scan DMSO {D:\Spectra} nmr 9

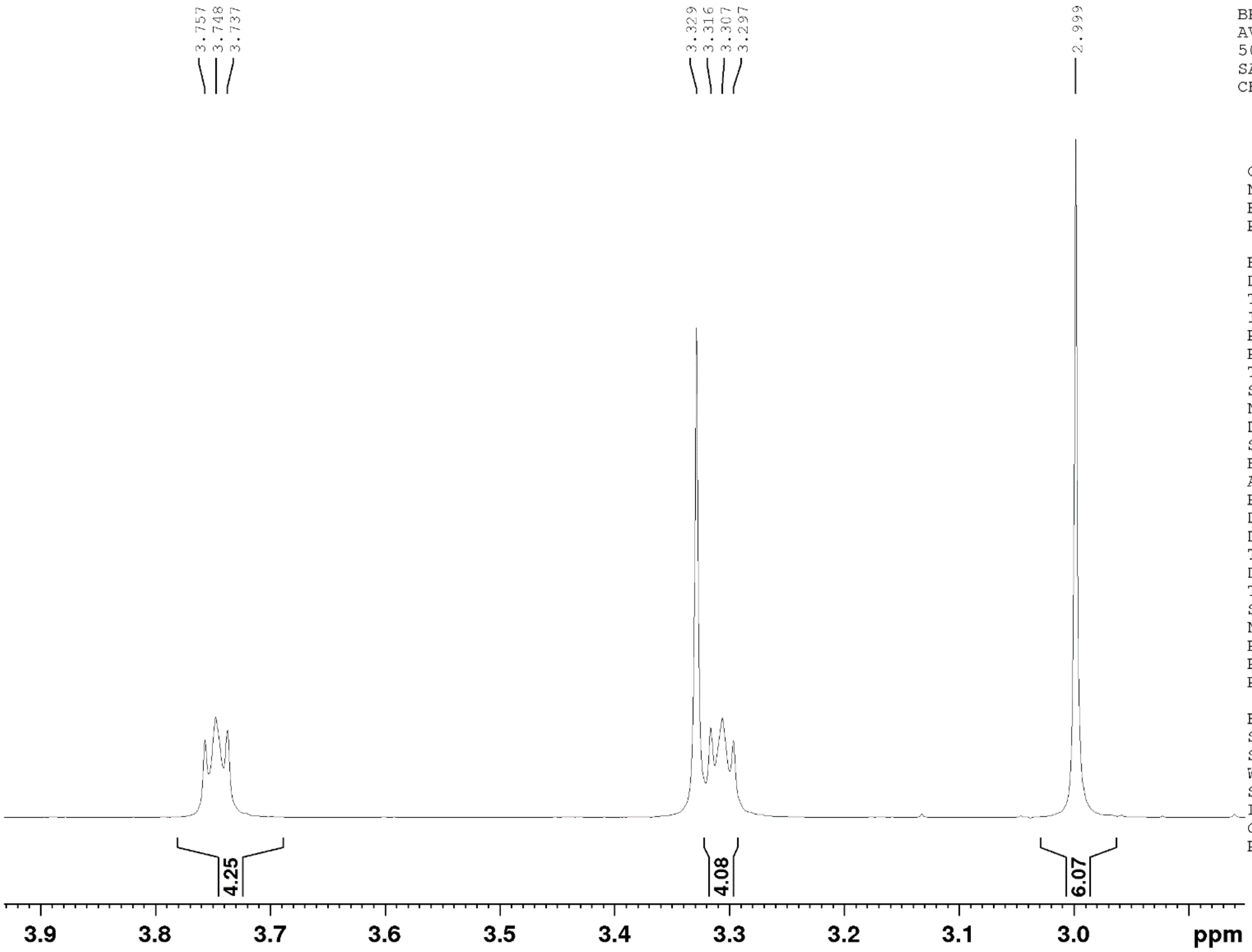

BRUKER
AVANCE NEO
500 MHz NMR SPE
SAIF, PANJAB UN
CHANDIGARH

Current Data P
NAME D
EXPNO
PROCNO

F2 - Acquisiti
Date_
Time
INSTRUM Avanc
PROBHD Z1194
PULPROG
TD
SOLVENT
NS
DS
SWH
FIDRES
AQ
RG
DW
DE
TE
D1 1
TD0
SFO1 50
NUC1
P0
P1
PLW1 22

F2 - Processin
SI
SF 50
WDW
SSB 0
LB
GB 0
PC

MO-5
C13CPD DMSO {D:\Spectra} nmr 9

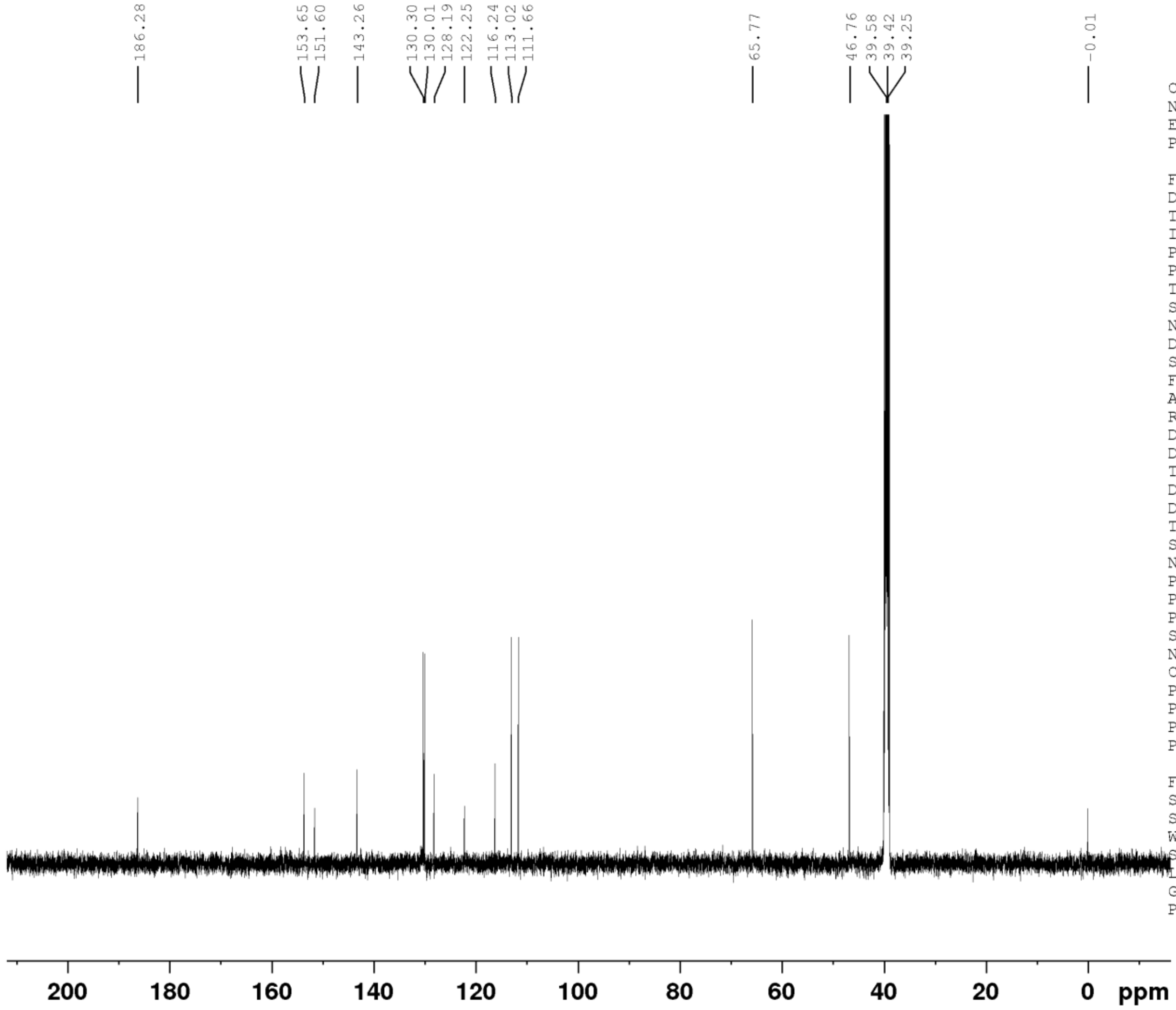

BRUKER
AVANCE NEO
500 MHz NMR SPECT
SAIF, PANJAB UNIV
CHANDIGARH

Current Data Parameters
NAME Dec24-2019
EXPNO 91
PROCNO 1

F2 - Acquisition Parameter
Date_ 20191224
Time 16.07 h
INSTRUM Avance Neo 500
PROBHD Z119470_0333 (
PULPROG zgpg30
TD 65536
SOLVENT DMSO
NS 512
DS 4
SWH 37037.035 Hz
FIDRES 1.130281 Hz
AQ 0.8847360 se
RG 101
DW 13.500 us
DE 6.50 us
TE 298.3 K
D1 2.00000000 se
D11 0.03000000 se
TD0 1
SFO1 125.7804233 MH
NUC1 13C
P0 3.33 us
P1 10.00 us
PLW1 79.56099701 W
SFO2 500.1720007 MH
NUC2 1H
CPDPRG[2 waltz65
PCPD2 80.00 us
PLW2 22.02300072 W
PLW12 0.34411001 W
PLW13 0.17308000 W

F2 - Processing parameters
SI 32768
SF 125.7679203 MH
WDW EM
SSB 0
LB 1.00 Hz
GB 0
PC 1.40

MO-5
C13CPD DMSO {D:\Spectra} nmr 9

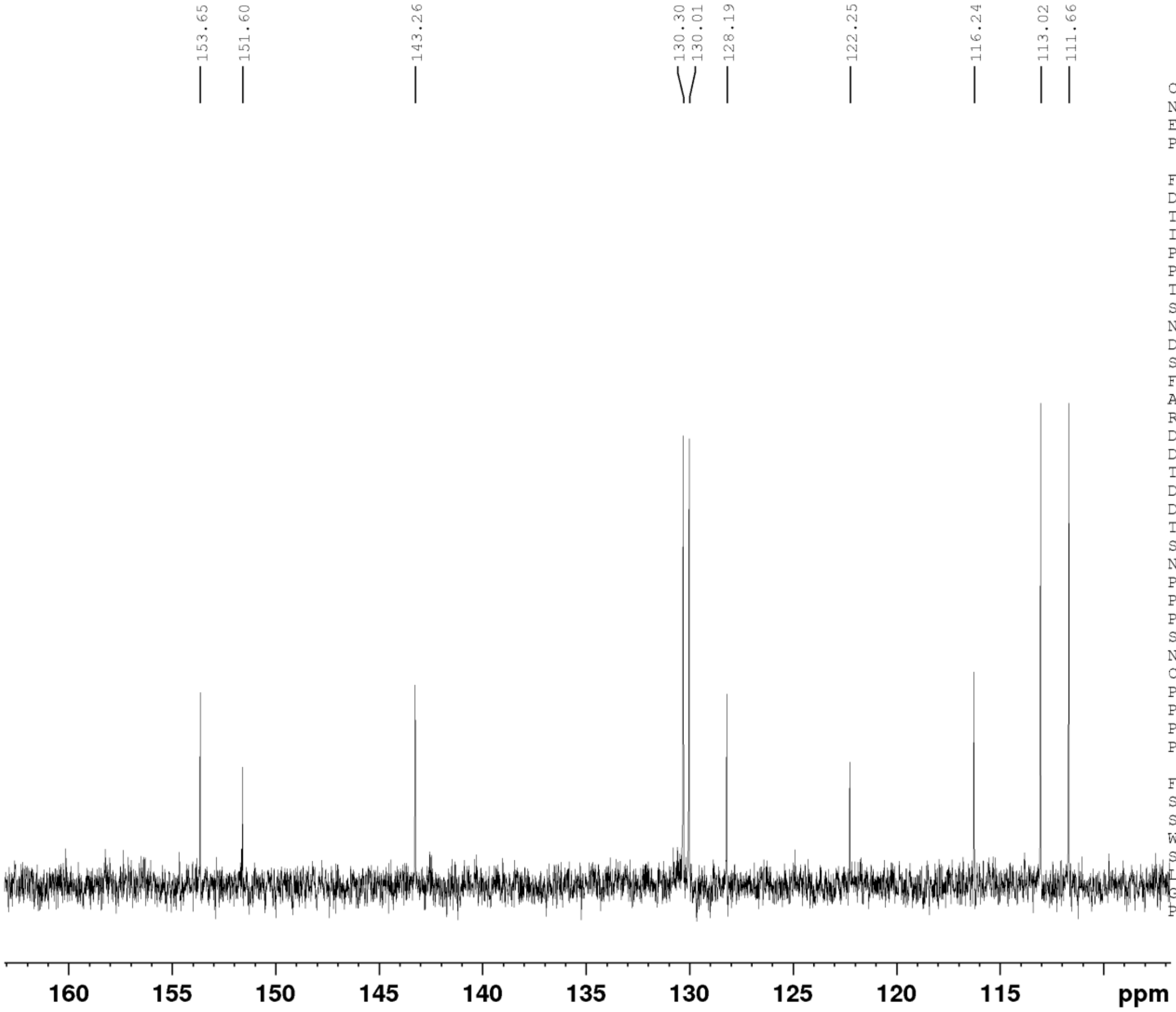

BRUKER
AVANCE NEO
500 MHz NMR SPECT
SAIF, PANJAB UNIV
CHANDIGARH

Current Data Parameters
NAME Dec24-2019
EXPNO 91
PROCNO 1

F2 - Acquisition Parameter
Date_ 20191224
Time 16.07 h
INSTRUM Avance Neo 500
PROBHD Z119470_0333 (
PULPROG zgpg30
TD 65536
SOLVENT DMSO
NS 512
DS 4
SWH 37037.035 Hz
FIDRES 1.130281 Hz
AQ 0.8847360 se
RG 101
DW 13.500 us
DE 6.50 us
TE 298.3 K
D1 2.00000000 se
D11 0.03000000 se
TD0 1
SFO1 125.7804233 MH
NUC1 13C
P0 3.33 us
P1 10.00 us
PLW1 79.56099701 W
SFO2 500.1720007 MH
NUC2 1H
CPDPRG[2 waltz65
PCPD2 80.00 us
PLW2 22.02300072 W
PLW12 0.34411001 W
PLW13 0.17308000 W

F2 - Processing parameters
SI 32768
SF 125.7679203 MH
WDW EM
SSB 0
LB 1.00 Hz
GB 0
PC 1.40

MO-5
C13CPD DMSO {D:\Spectra} nmr 9

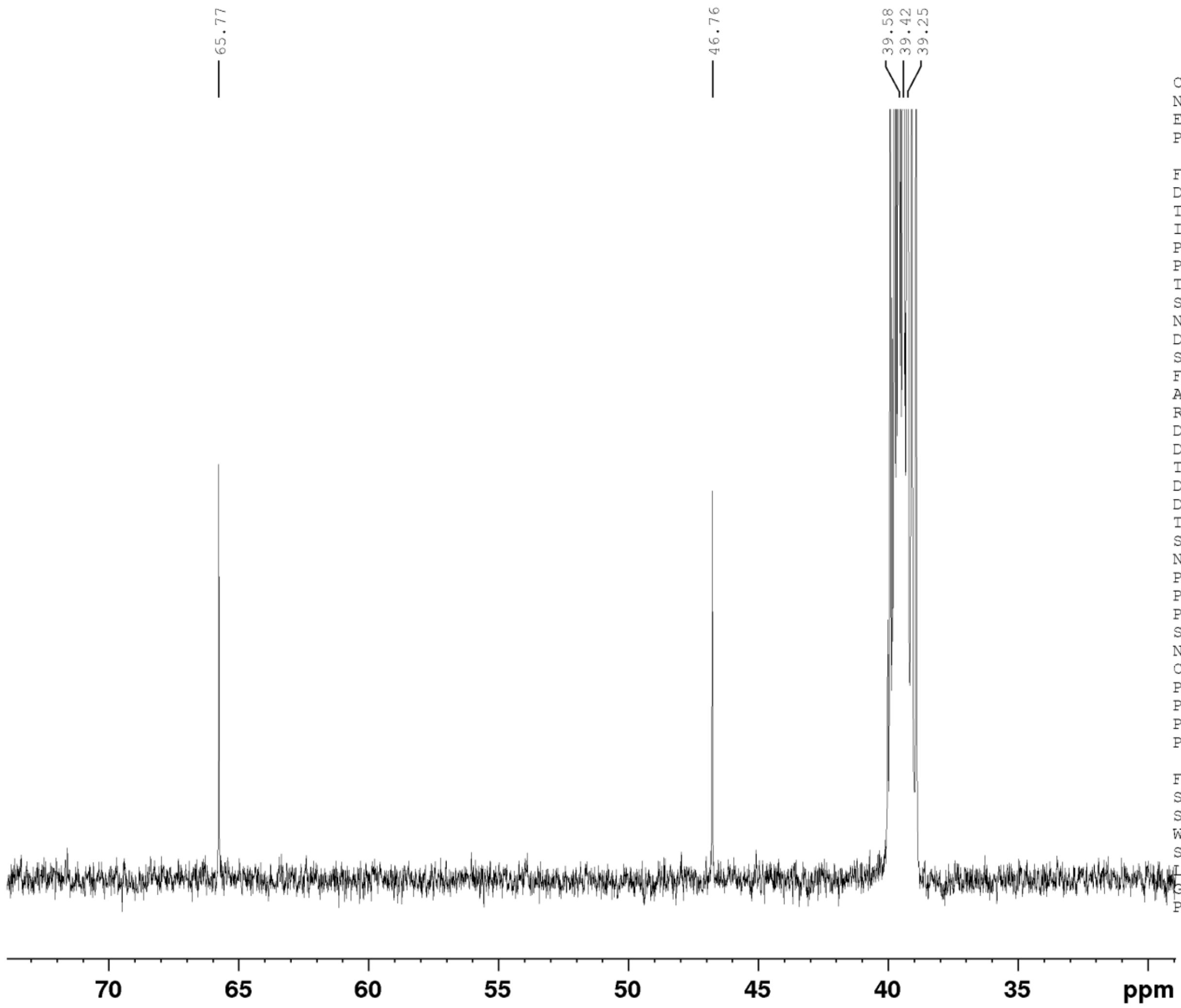

BRUKER
AVANCE NEO
500 MHz NMR SPECT
SAIF, PANJAB UNIV
CHANDIGARH

Current Data Parameters
NAME Dec24-2019
EXPNO 91
PROCNO 1

F2 - Acquisition Parameters
Date_ 20191224
Time 16.07 h
INSTRUM Avance Neo 500
PROBHD Z119470_0333 (
PULPROG zgpg30
TD 65536
SOLVENT DMSO
NS 512
DS 4
SWH 37037.035 Hz
FIDRES 1.130281 Hz
AQ 0.8847360 se
RG 101
DW 13.500 us
DE 6.50 us
TE 298.3 K
D1 2.00000000 se
D11 0.03000000 se
TD0 1
SFO1 125.7804233 MH
NUC1 13C
P0 3.33 us
P1 10.00 us
PLW1 79.56099701 W
SFO2 500.1720007 MH
NUC2 1H
CPDPRG[2 waltz65
PCPD2 80.00 us
PLW2 22.02300072 W
PLW12 0.34411001 W
PLW13 0.17308000 W

F2 - Processing parameters
SI 32768
SF 125.7679203 MH
WDW EM
SSB 0
LB 1.00 Hz
GB 0
PC 1.40

MO-6
1H_8scan DMSO {D:\Spectra} nmr 10

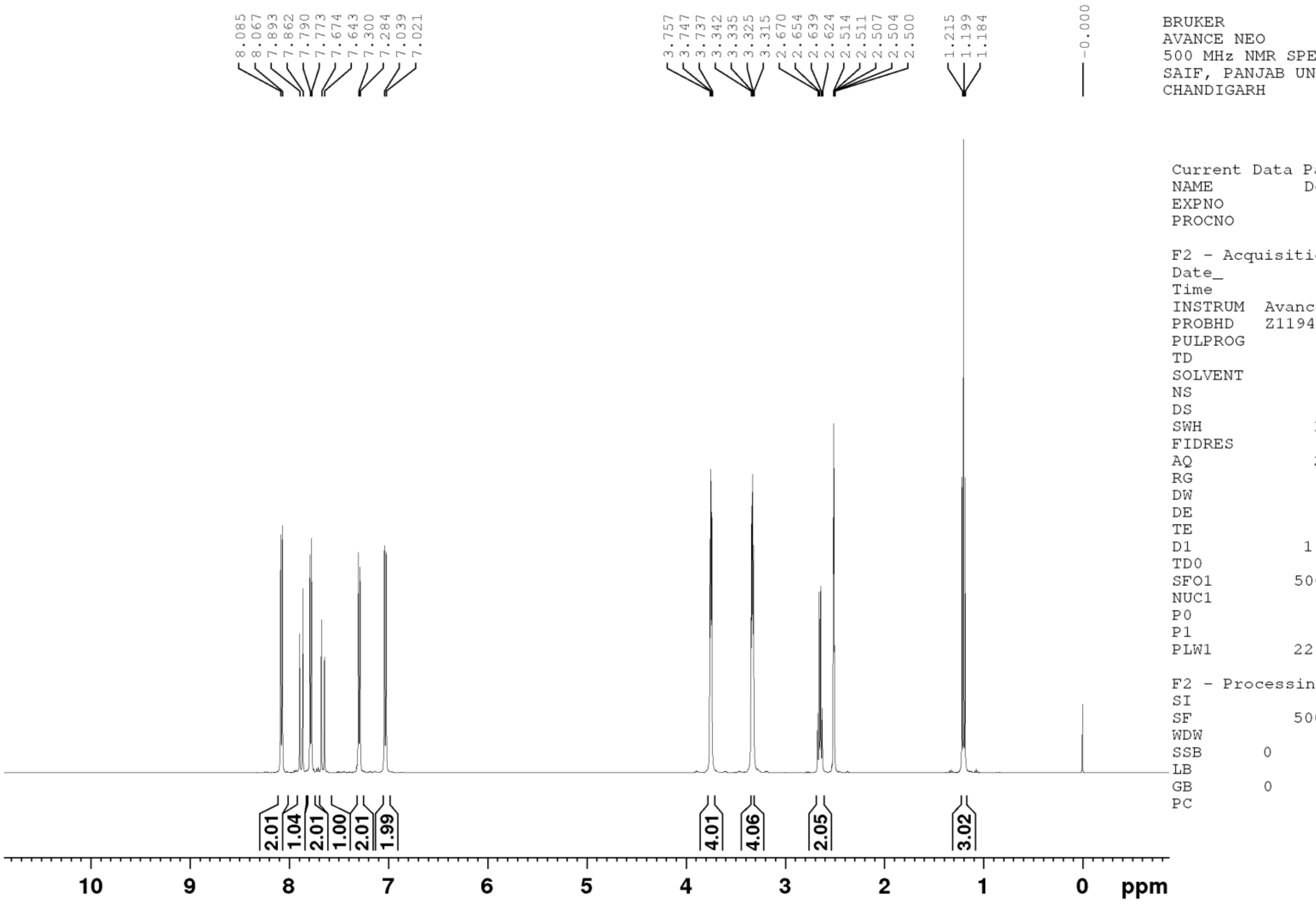

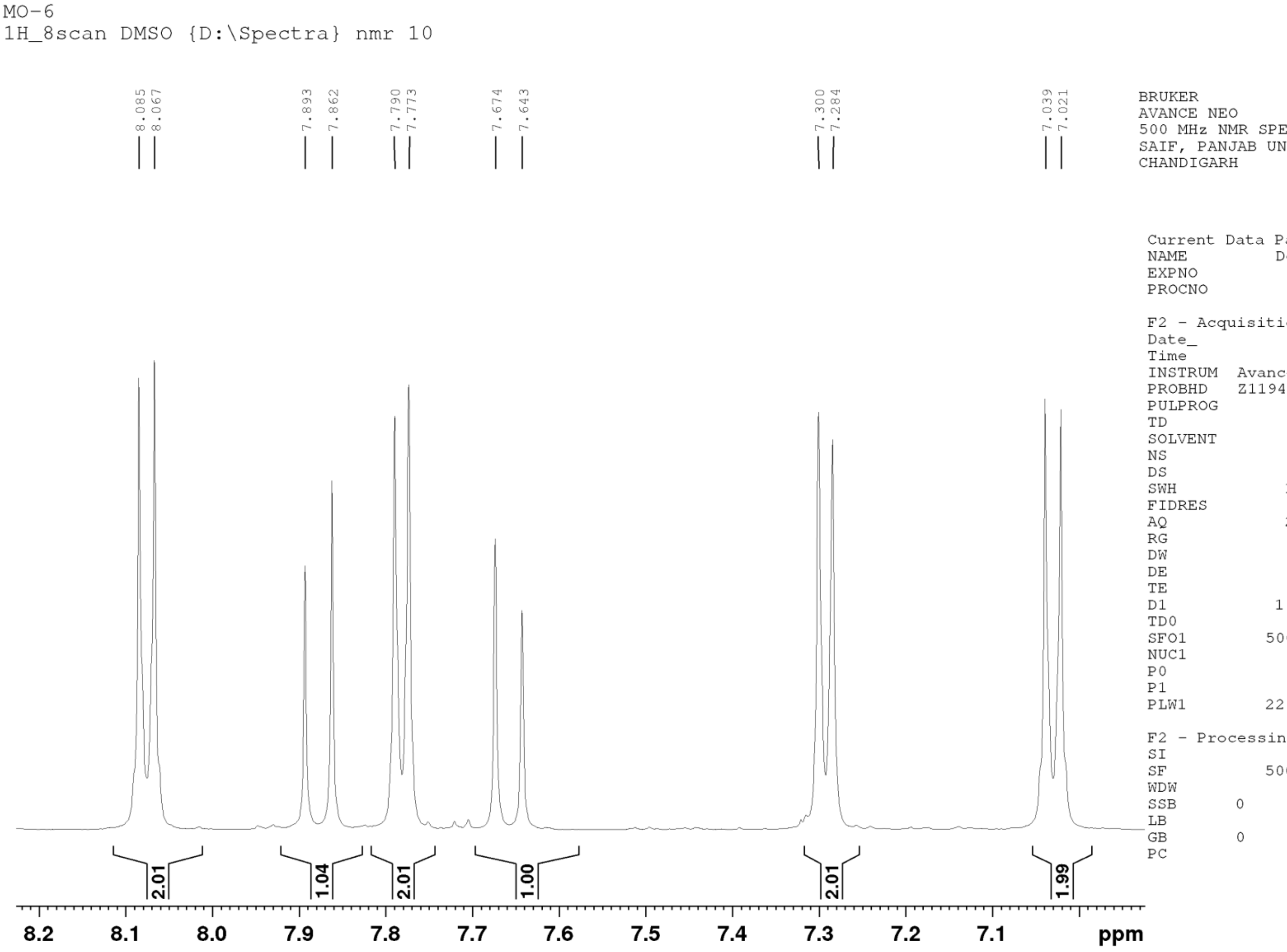
MO-6
1H_8scan DMSO {D:\Spectra} nmr 10
8.085
8.067
7.893
7.862
7.790
7.773
7.674
7.643
7.300
7.284
7.039
7.021
BRUKER
AVANCE NEO
500 MHz NMR SPE
SAIF, PANJAB UN
CHANDIGARH
Current Data P
NAME
EXPNO
PROCNO
F2 - Acquisiti
Date_
Time
INSTRUM Avanc
PROBHD Z1194
PULPROG
TD
SOLVENT
NS
DS
SWH
FIDRES
AQ
RG
DW
DE
TE
D1
TD0
SFO1
NUC1
P0
P1
PLW1
F2 - Processin
SI
SF
WDW
SSB 0
LB
GB 0
PC
2.01
1.04
2.01
1.00
2.01
1.99
8.2
8.1
8.0
7.9
7.8
7.7
7.6
7.5
7.4
7.3
7.2
7.1
ppm

MO-6
1H_8scan DMSO {D:\Spectra} nmr 10

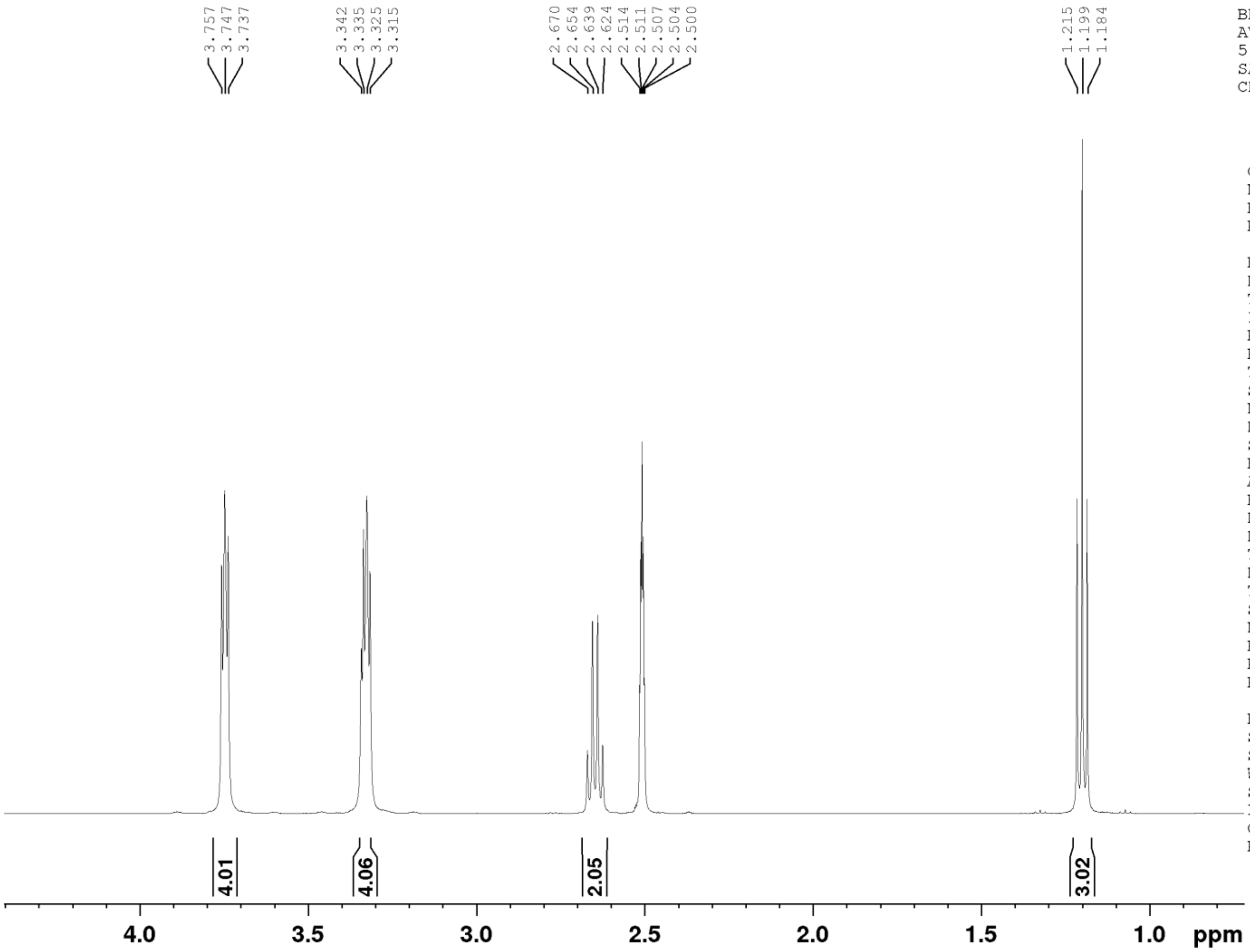

BRUKER
AVANCE NEO
500 MHz NMR SPE
SAIF, PANJAB UN
CHANDIGARH

Current Data P
NAME D
EXPNO
PROCNO

F2 - Acquisiti
Date_
Time
INSTRUM Avanc
PROBHD Z1194
PULPROG
TD
SOLVENT
NS
DS
SWH
FIDRES
AQ
RG
DW
DE
TE
D1 1
TD0
SFO1 50
NUC1
P0
P1
PLW1 22

F2 - Processin
SI
SF 50
WDW
SSB 0
LB
GB 0
PC

MO-6
C13CPD DMSO {D:\Spectra} nmr 10

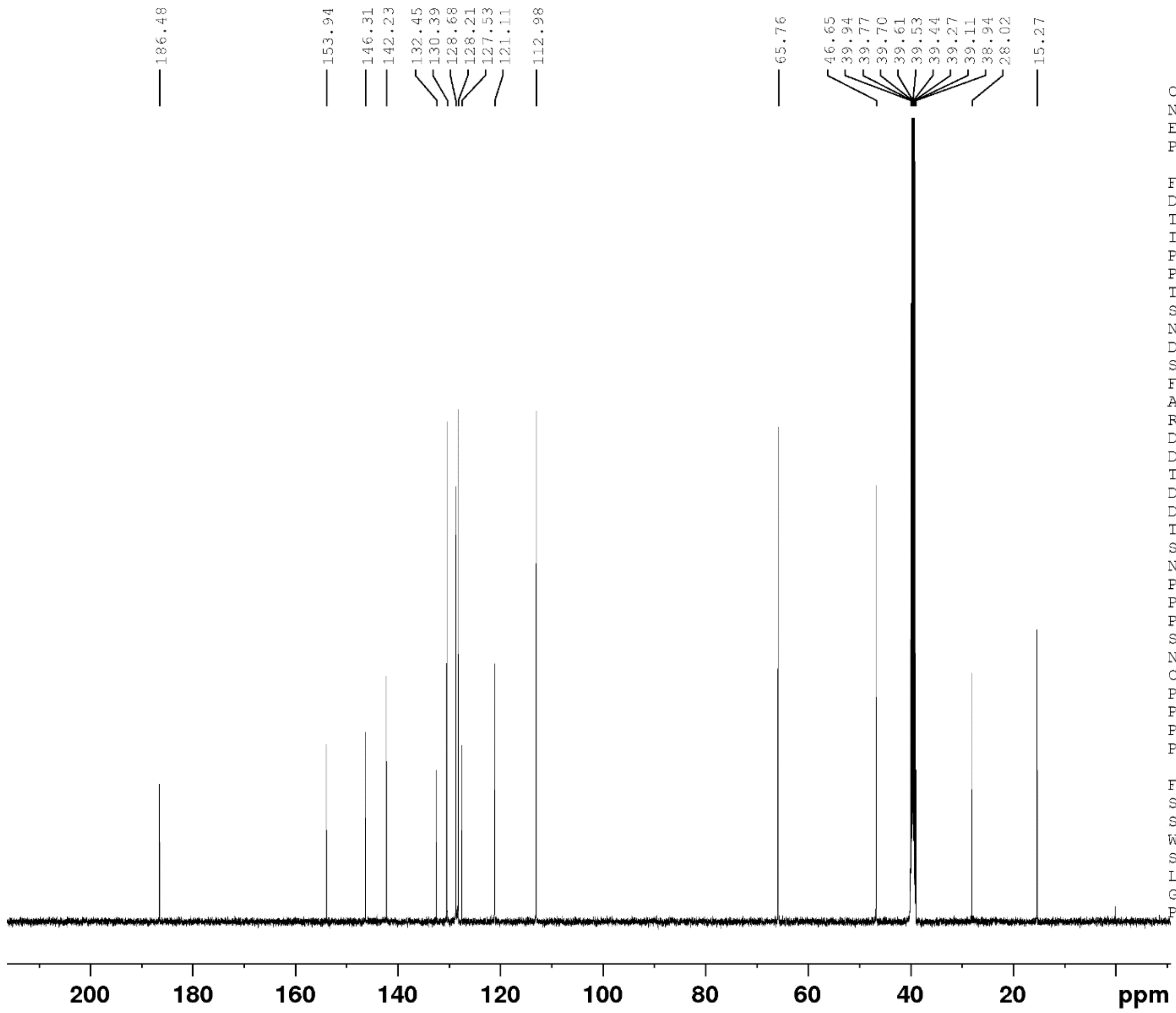

BRUKER
AVANCE NEO
500 MHz NMR SPECT
SAIF, PANJAB UNIV
CHANDIGARH

Current Data Parameters
NAME Dec24-2019
EXPNO 101
PROCNO 1

F2 - Acquisition Parameter
Date_ 20191224
Time 16.35 h
INSTRUM Avance Neo 500
PROBHD Z119470_0333 (
PULPROG zgpg30
TD 65536
SOLVENT DMSO
NS 512
DS 4
SWH 37037.035 Hz
FIDRES 1.130281 Hz
AQ 0.8847360 se
RG 101
DW 13.500 us
DE 6.50 us
TE 298.4 K
D1 2.00000000 se
D11 0.03000000 se
TD0 1
SFO1 125.7804233 MH
NUC1 13C
P0 3.33 us
P1 10.00 us
PLW1 79.56099701 W
SFO2 500.1720007 MH
NUC2 1H
CPDPRG[2 waltz65
PCPD2 80.00 us
PLW2 22.02300072 W
PLW12 0.34411001 W
PLW13 0.17308000 W

F2 - Processing parameters
SI 32768
SF 125.7679178 MH
WDW EM
SSB 0
LB 1.00 Hz
GB 0
PC 1.40

MO-6
C13CPD DMSO {D:\Spectra} nmr 10

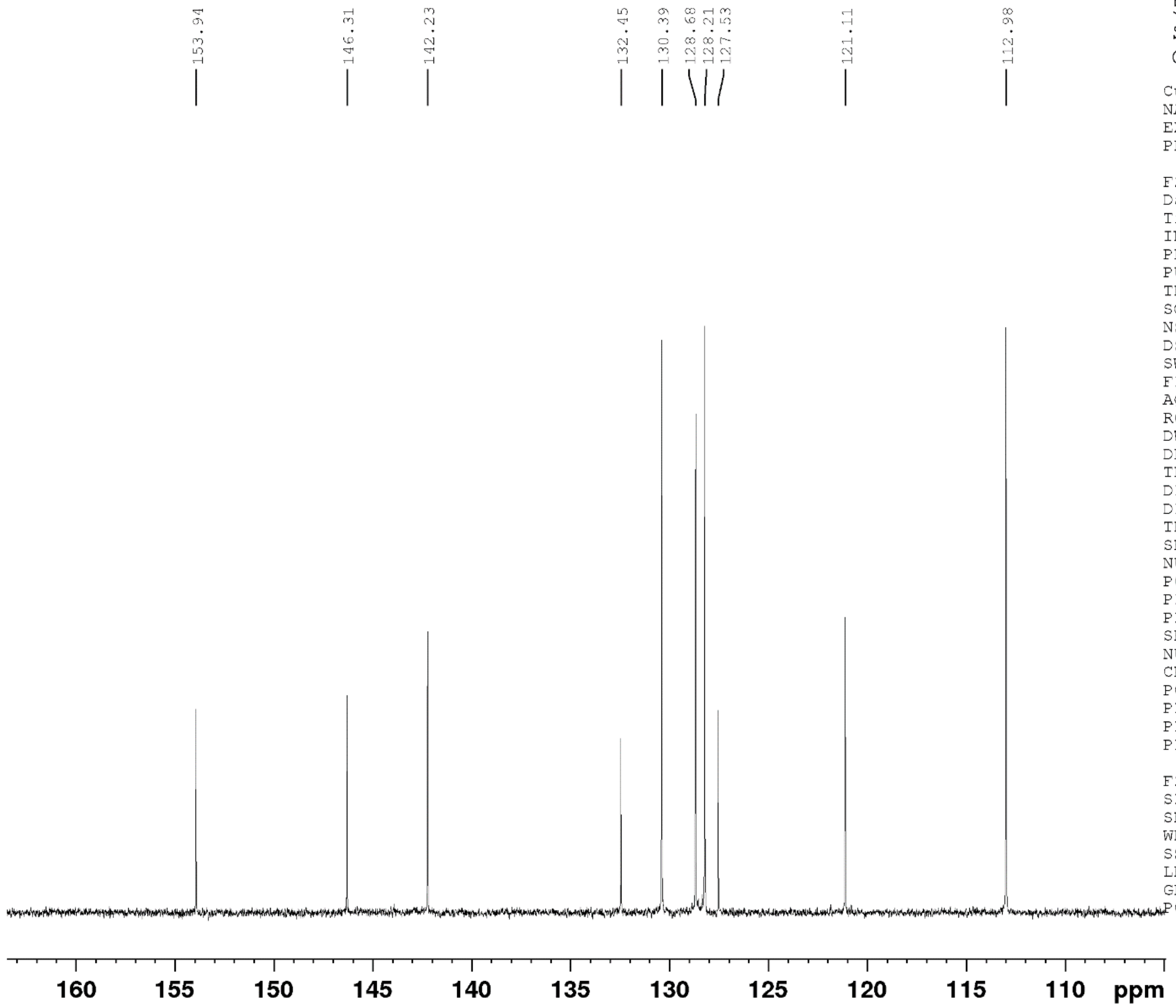

BRUKER
AVANCE NEO
500 MHz NMR SPECT
SAIF, PANJAB UNIV
CHANDIGARH

Current Data Parameters
NAME Dec24-2019
EXPNO 101
PROCNO 1

F2 - Acquisition Parameters
Date_ 20191224
Time 16.35 h
INSTRUM Avance Neo 500
PROBHD Z119470_0333 (
PULPROG zgpg30
TD 65536
SOLVENT DMSO
NS 512
DS 4
SWH 37037.035 Hz
FIDRES 1.130281 Hz
AQ 0.8847360 se
RG 101
DW 13.500 us
DE 6.50 us
TE 298.4 K
D1 2.00000000 se
D11 0.03000000 se
TD0 1
SFO1 125.7804233 MH
NUC1 13C
P0 3.33 us
P1 10.00 us
PLW1 79.56099701 W
SFO2 500.1720007 MH
NUC2 1H
CPDPRG[2 waltz65
PCPD2 80.00 us
PLW2 22.02300072 W
PLW12 0.34411001 W
PLW13 0.17308000 W

F2 - Processing parameters
SI 32768
SF 125.7679178 MH
WDW EM
SSB 0
LB 1.00 Hz
GB 0
PC 1.40

MO-6
C13CPD DMSO {D:\Spectra} nmr 10

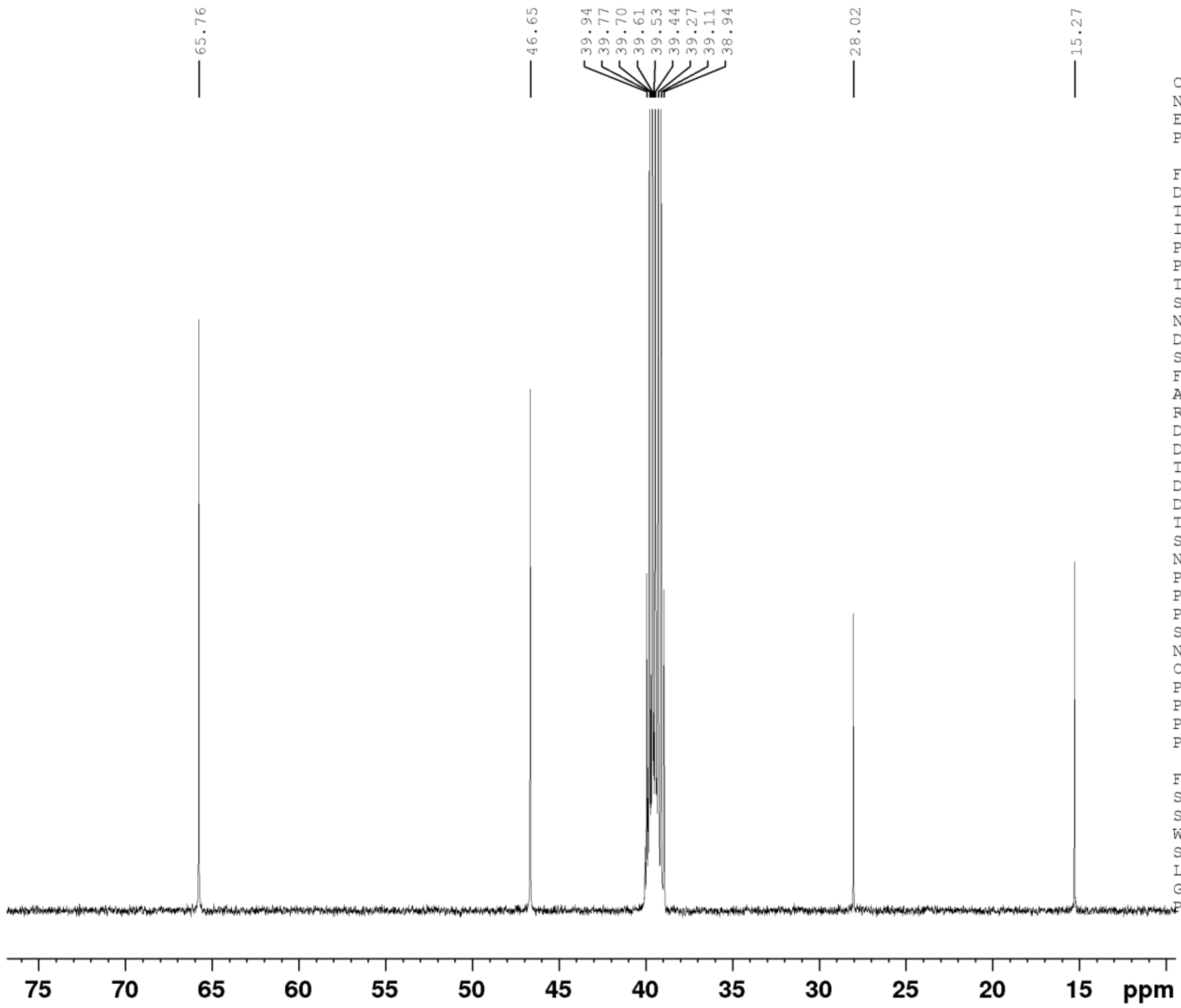

BRUKER
AVANCE NEO
500 MHz NMR SPECT
SAIF, PANJAB UNIV
CHANDIGARH

Current Data Parameters
NAME Dec24-2019
EXPNO 101
PROCNO 1

F2 - Acquisition Parameters
Date_ 20191224
Time 16.35 h
INSTRUM Avance Neo 500
PROBHD Z119470_0333 (
PULPROG zgpg30
TD 65536
SOLVENT DMSO
NS 512
DS 4
SWH 37037.035 Hz
FIDRES 1.130281 Hz
AQ 0.8847360 se
RG 101
DW 13.500 us
DE 6.50 us
TE 298.4 K
D1 2.00000000 sec
D11 0.03000000 sec
TD0 1
SFO1 125.7804233 MHz
NUC1 13C
P0 3.33 us
P1 10.00 us
PLW1 79.56099701 W
SFO2 500.1720007 MHz
NUC2 1H
CPDPRG[2 waltz65
PCPD2 80.00 us
PLW2 22.02300072 W
PLW12 0.34411001 W
PLW13 0.17308000 W

F2 - Processing parameters
SI 32768
SF 125.7679178 MHz
WDW EM
SSB 0
LB 1.00 Hz
GB 0
PC 1.40

MO-7
1H_8scan DMSO {D:\Spectra} nmr 11

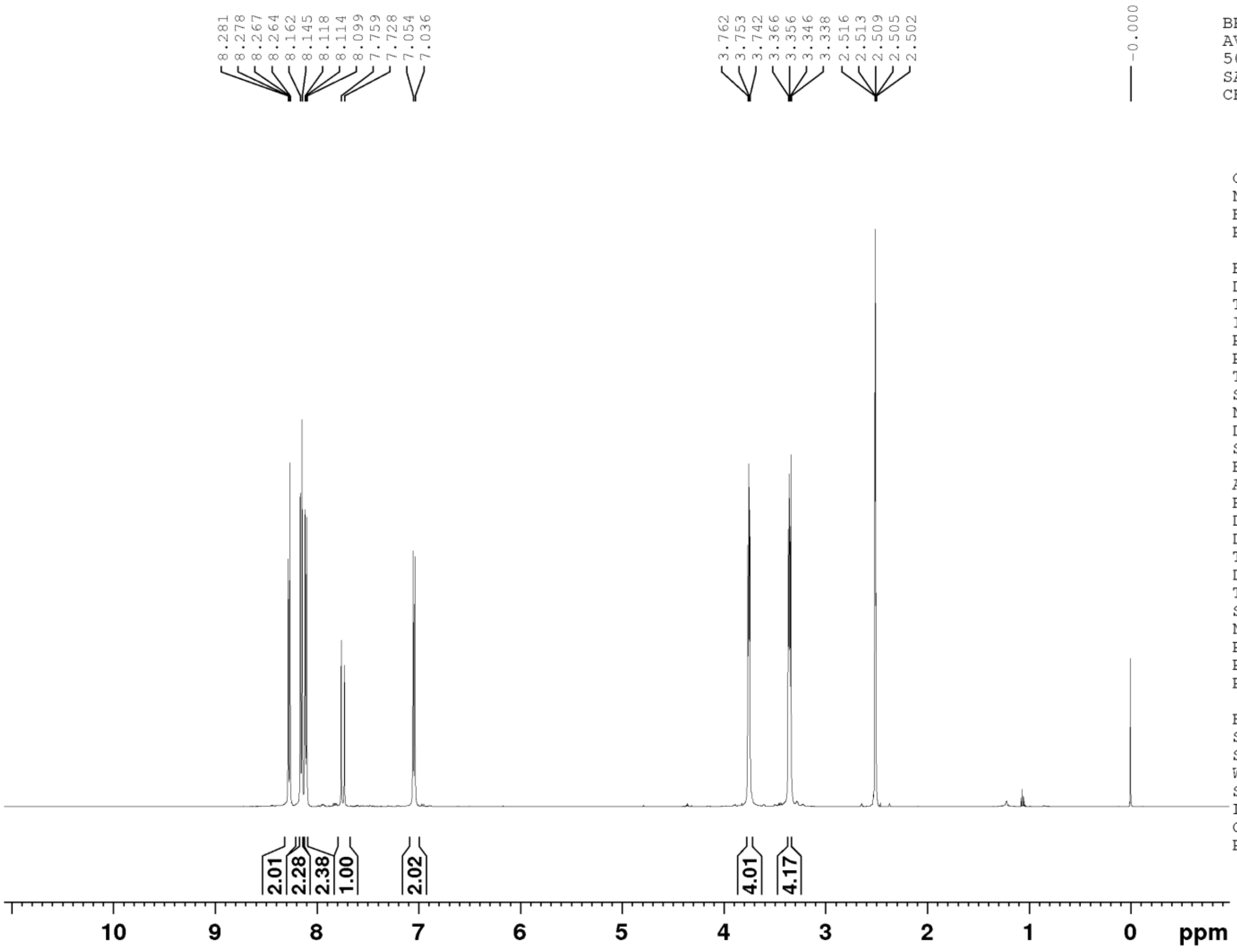

BRUKER
AVANCE NEO
500 MHz NMR SPE
SAIF, PANJAB UN
CHANDIGARH

Current Data Pa
NAME D
EXPNO
PROCNO

F2 - Acquisiti
Date_
Time
INSTRUM Avanc
PROBHD Z1194
PULPROG
TD
SOLVENT
NS
DS
SWH
FIDRES
AQ
RG
DW
DE
TE
D1 1
TD0
SFO1 50
NUC1
P0
P1
PLW1 22

F2 - Processin
SI
SF 50
WDW
SSB 0
LB
GB 0
PC

MO-7
1H_8scan DMSO {D:\Spectra} nmr 11

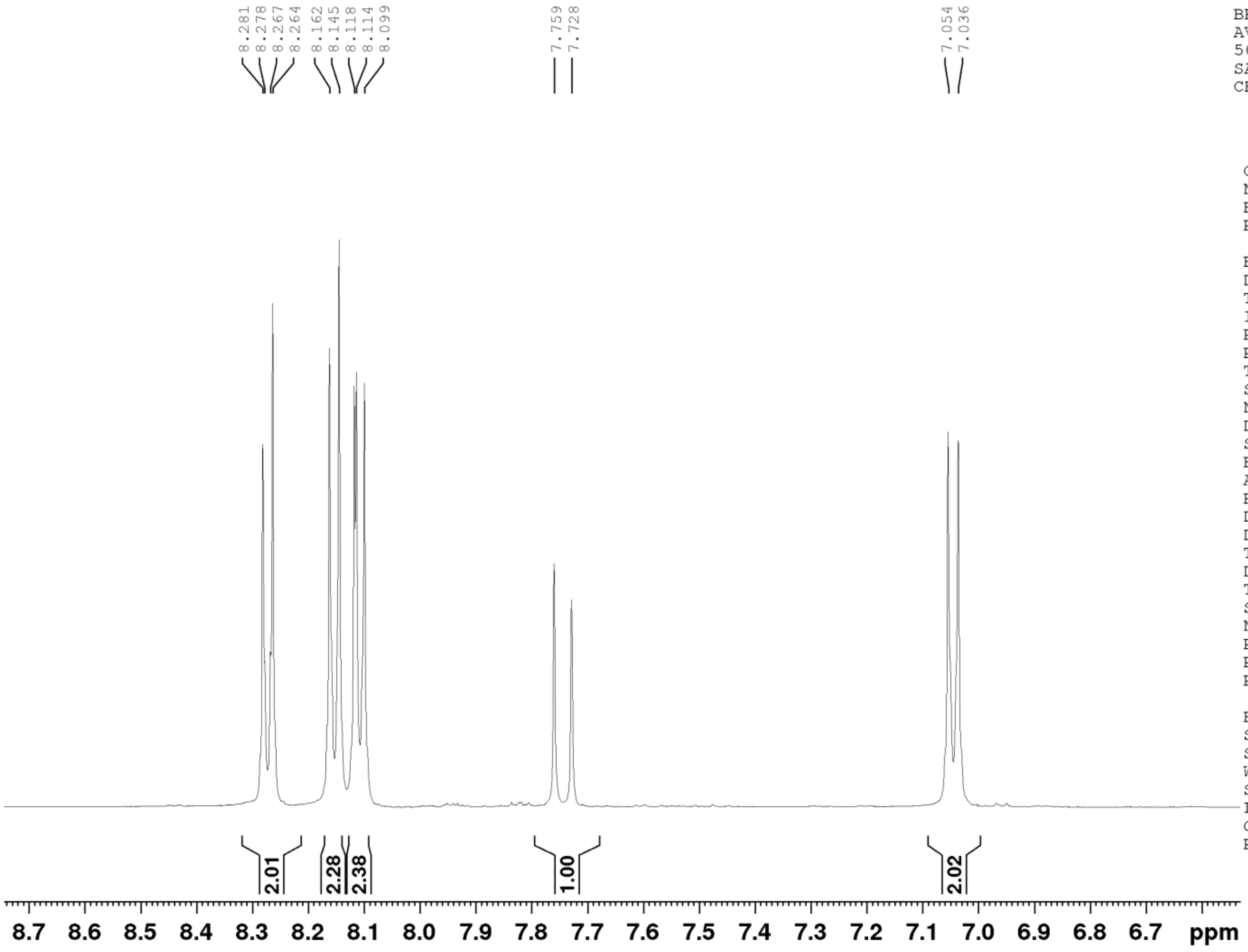

BRUKER
AVANCE NEO
500 MHz NMR SPE
SAIF, PANJAB UN
CHANDIGARH

Current Data P
NAME D
EXPNO
PROCNO

F2 - Acquisiti
Date_
Time
INSTRUM Avanc
PROBHD Z1194
PULPROG
TD
SOLVENT
NS
DS
SWH
FIDRES
AQ
RG
DW
DE
TE
D1 1
TD0
SFO1 50
NUC1
P0
P1
PLW1 22

F2 - Processin
SI
SF 50
WDW
SSB 0
LB
GB 0
PC

MO-7
1H_8scan DMSO {D:\Spectra} nmr 11

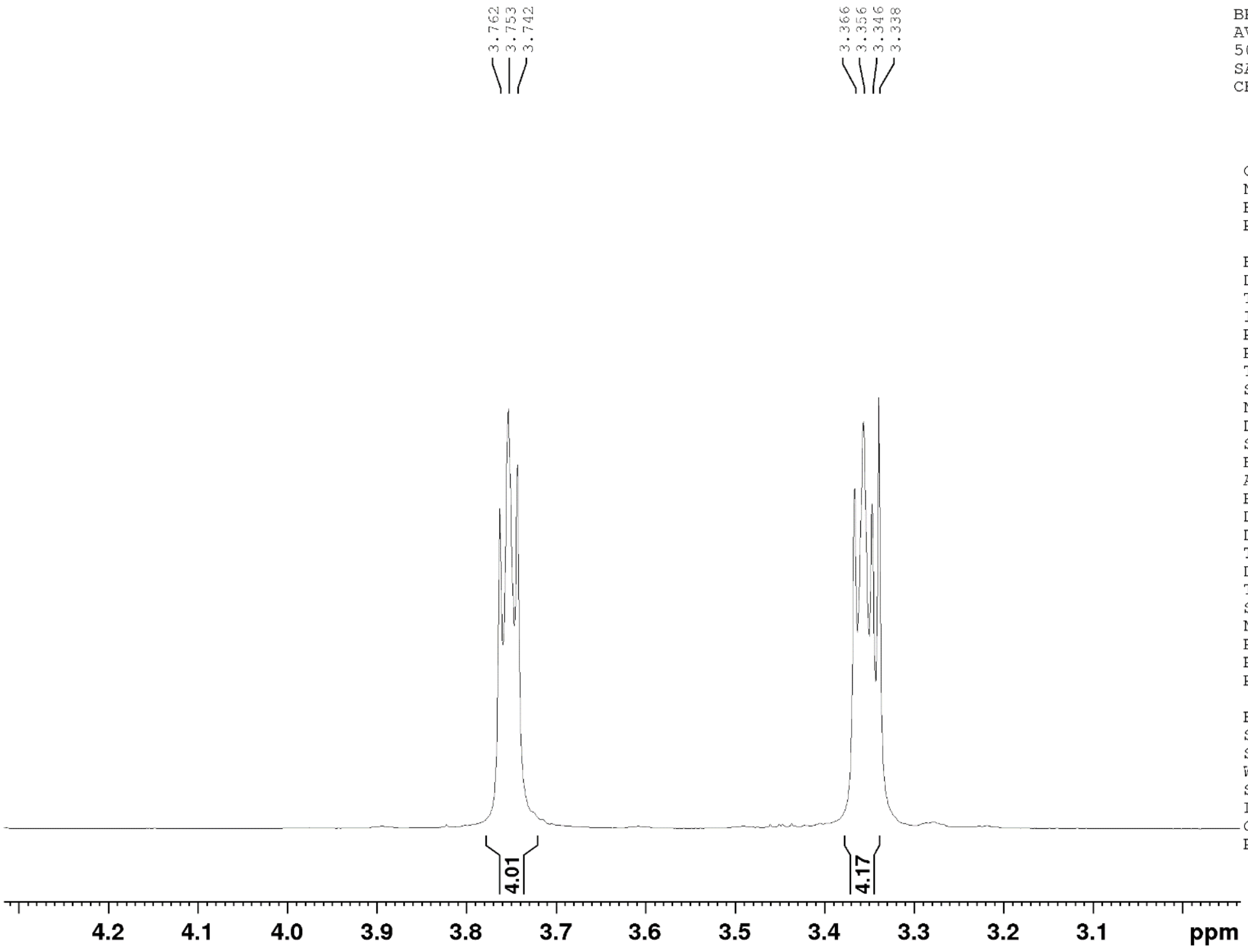

BRUKER
AVANCE NEO
500 MHz NMR SPE
SAIF, PANJAB UN
CHANDIGARH

Current Data P
NAME D
EXPNO
PROCNO

F2 - Acquisiti
Date_
Time
INSTRUM Avanc
PROBHD Z1194
PULPROG
TD
SOLVENT
NS
DS
SWH
FIDRES
AQ
RG
DW
DE
TE
D1 1
TD0
SFO1 50
NUC1
P0
P1
PLW1 22

F2 - Processin
SI
SF 50
WDW
SSB 0
LB
GB 0
PC

MO-7
C13CPD DMSO {D:\Spectra} nmr 11

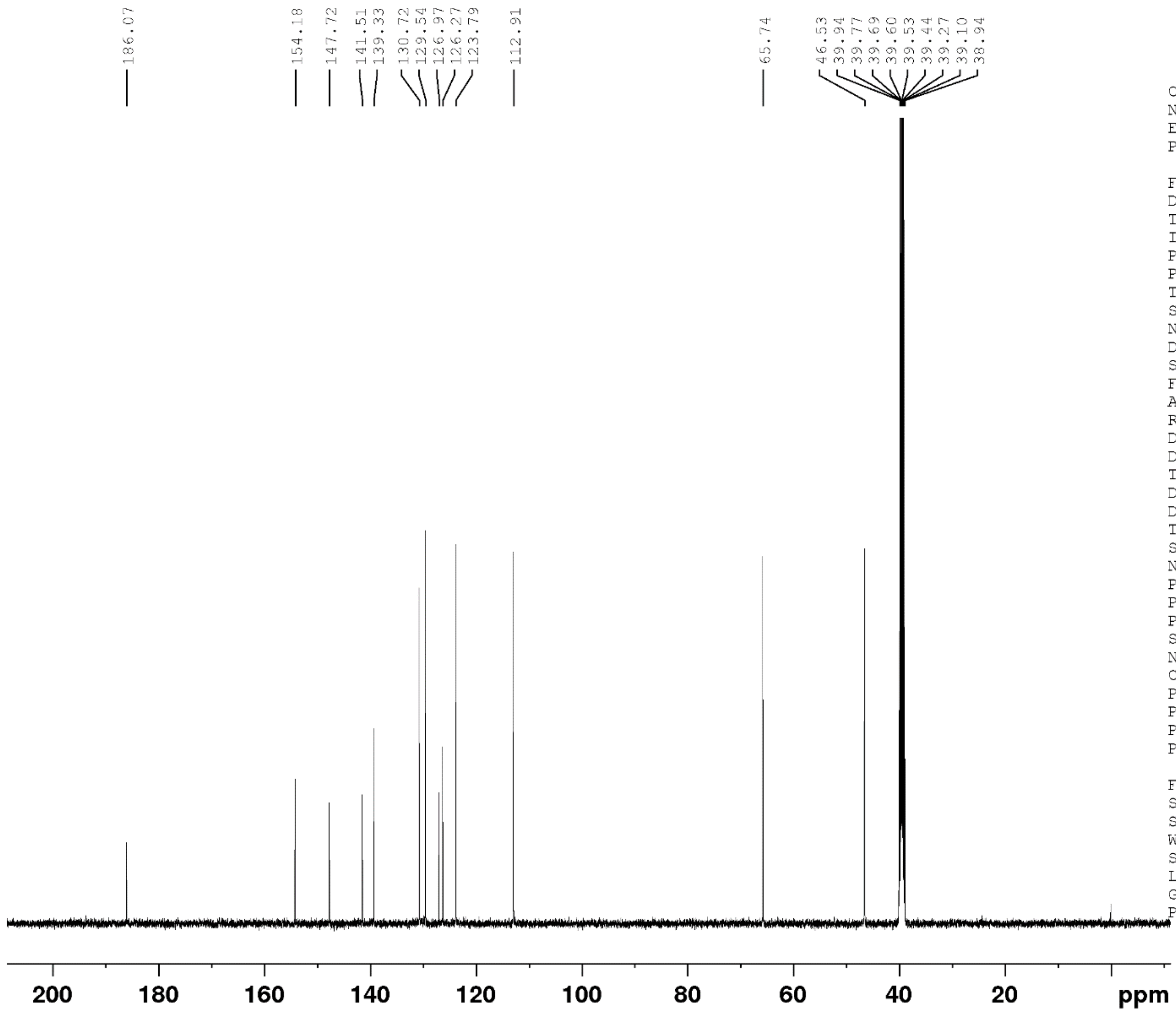

BRUKER
AVANCE NEO
500 MHz NMR SPECT
SAIF, PANJAB UNIV
CHANDIGARH

Current Data Parameters

NAME	Dec24-2019
EXPNO	111
PROCNO	1

F2 - Acquisition Parameter

Date_	20191224
Time	17.03 h
INSTRUM	Avance Neo 500
PROBHD	Z119470_0333 (
PULPROG	zgpg30
TD	65536
SOLVENT	DMSO
NS	512
DS	4
SWH	37037.035 Hz
FIDRES	1.130281 Hz
AQ	0.8847360 se
RG	101
DW	13.500 us
DE	6.50 us
TE	298.4 K
D1	2.00000000 se
D11	0.03000000 se
TD0	1
SFO1	125.7804233 MH
NUC1	13C
P0	3.33 us
P1	10.00 us
PLW1	79.56099701 W
SFO2	500.1720007 MH
NUC2	1H
CPDPRG[2	waltz65
PCPD2	80.00 us
PLW2	22.02300072 W
PLW12	0.34411001 W
PLW13	0.17308000 W

F2 - Processing parameters

SI	32768
SF	125.7679179 MH
WDW	EM
SSB	0
LB	1.00 Hz
GB	0
PC	1.40

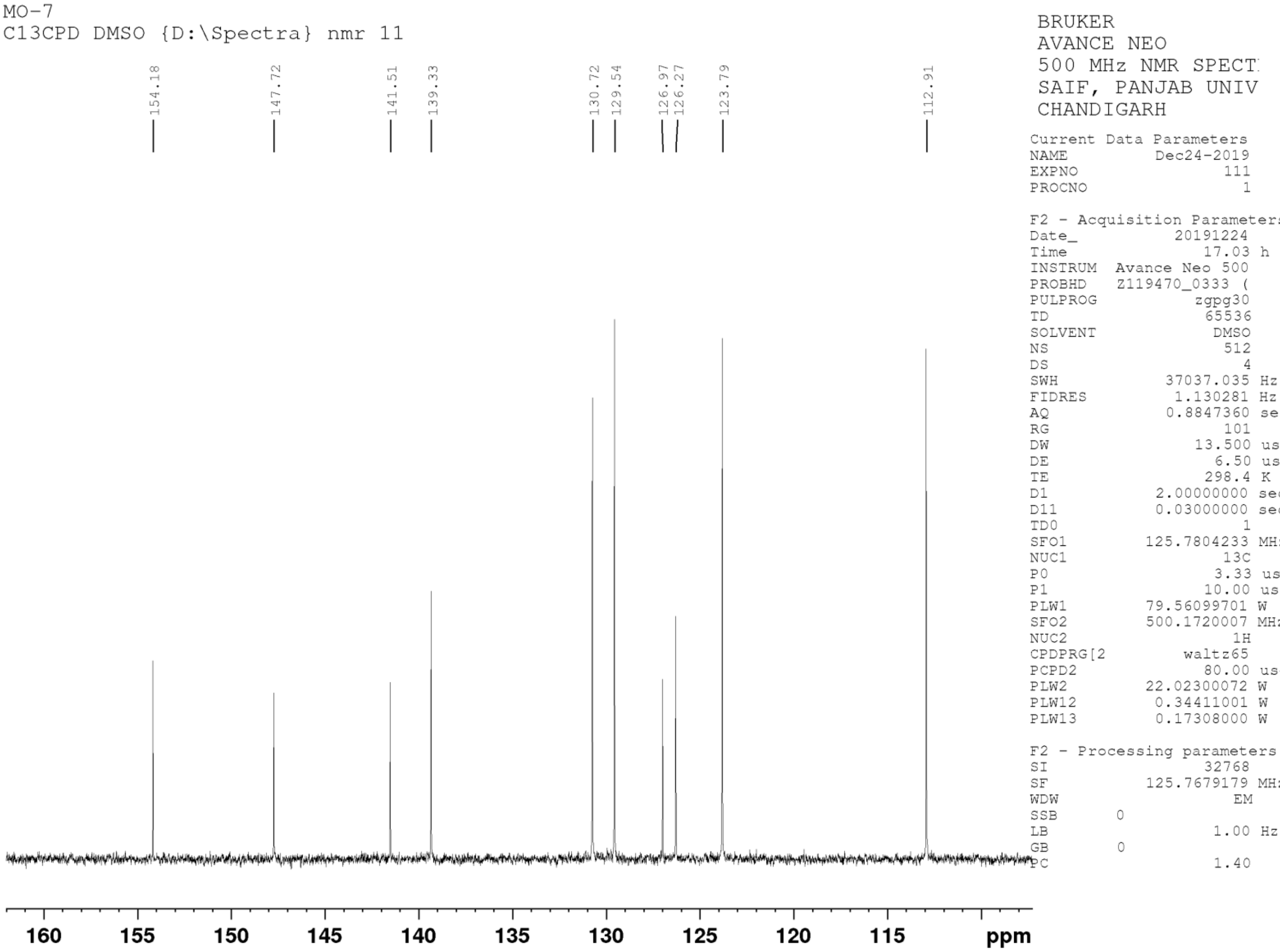
MO-7
C13CPD DMSO {D:\Spectra} nmr 11
154.18
147.72
141.51
139.33
130.72
129.54
126.97
126.27
123.79
112.91
160
155
150
145
140
135
130
125
120
115
ppm
BRUKER
AVANCE NEO
500 MHz NMR SPECT
SAIF, PANJAB UNIV
CHANDIGARH
Current Data Parameters
NAME Dec24-2019
EXPNO 111
PROCNO 1
F2 - Acquisition Parameters
Date_ 20191224
Time 17.03 h
INSTRUM Avance Neo 500
PROBHD Z119470_0333 (
PULPROG zgpg30
TD 65536
SOLVENT DMSO
NS 512
DS 4
SWH 37037.035 Hz
FIDRES 1.130281 Hz
AQ 0.8847360 se
RG 101
DW 13.500 us
DE 6.50 us
TE 298.4 K
D1 2.00000000 se
D11 0.03000000 se
TD0 1
SFO1 125.7804233 MH
NUC1 13C
P0 3.33 us
P1 10.00 us
PLW1 79.56099701 W
SFO2 500.1720007 MH
NUC2 1H
CPDPRG[2 waltz65
PCPD2 80.00 us
PLW2 22.02300072 W
PLW12 0.34411001 W
PLW13 0.17308000 W
F2 - Processing parameters
SI 32768
SF 125.7679179 MH
WDW EM
SSB 0
LB 1.00 Hz
GB 0
PC 1.40

MO-7
C13CPD DMSO {D:\Spectra} nmr 11

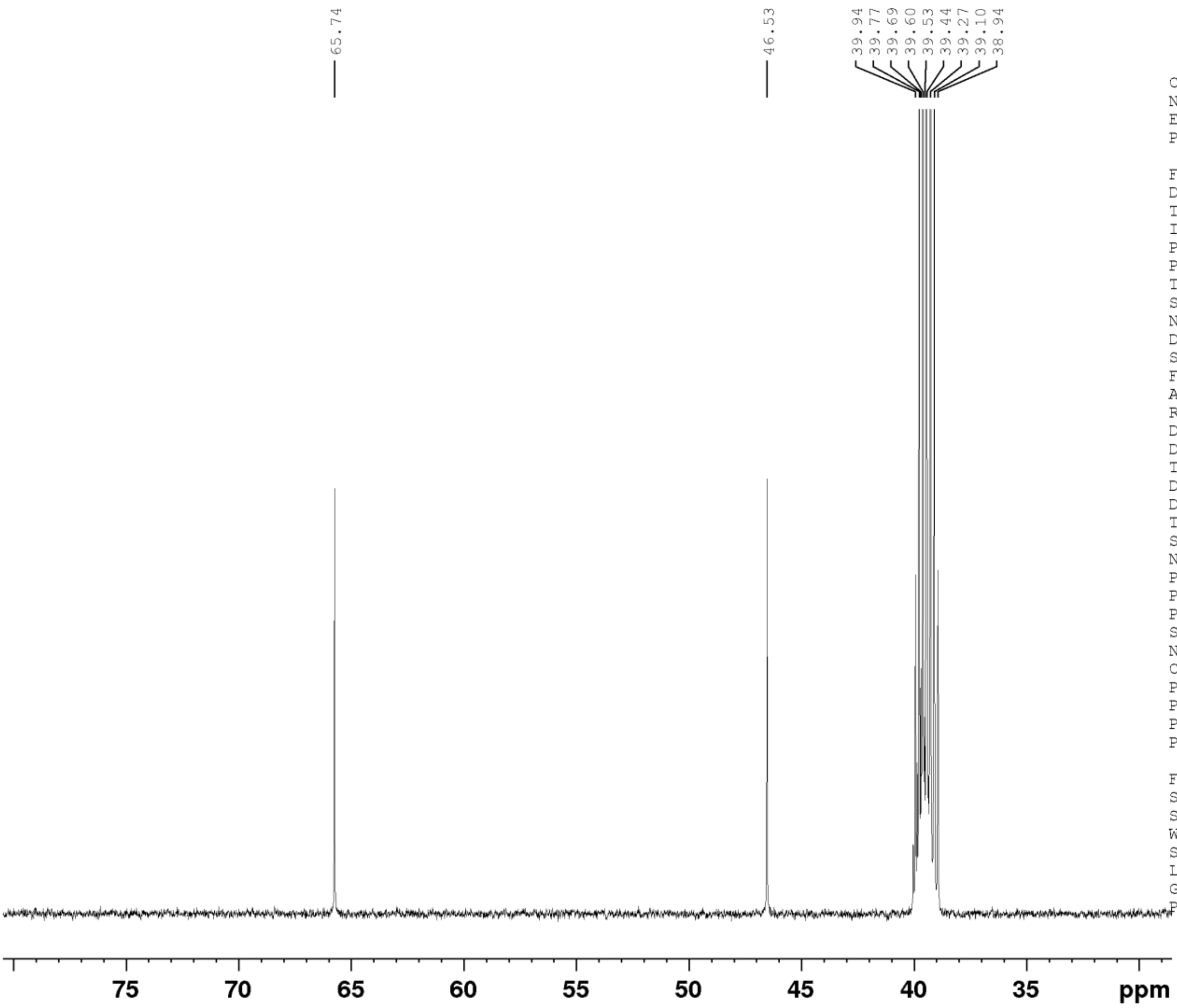

BRUKER
AVANCE NEO
500 MHz NMR SPECT
SAIF, PANJAB UNIV
CHANDIGARH

Current Data Parameters
NAME Dec24-2019
EXPNO 111
PROCNO 1

F2 - Acquisition Parameters
Date_ 20191224
Time 17.03 h
INSTRUM Avance Neo 500
PROBHD Z119470_0333 (
PULPROG zgpg30
TD 65536
SOLVENT DMSO
NS 512
DS 4
SWH 37037.035 Hz
FIDRES 1.130281 Hz
AQ 0.8847360 se
RG 101
DW 13.500 us
DE 6.50 us
TE 298.4 K
D1 2.00000000 se
D11 0.03000000 se
TD0 1
SFO1 125.7804233 MH
NUC1 13C
P0 3.33 us
P1 10.00 us
PLW1 79.56099701 W
SFO2 500.1720007 MH
NUC2 1H
CPDPRG[2 waltz65
PCPD2 80.00 us
PLW2 22.02300072 W
PLW12 0.34411001 W
PLW13 0.17308000 W

F2 - Processing parameters
SI 32768
SF 125.7679179 MH
WDW EM
SSB 0
LB 1.00 Hz
GB 0
PC 1.40

MO-8
1H_8scan DMSO {D:\Spectra} nmr 12

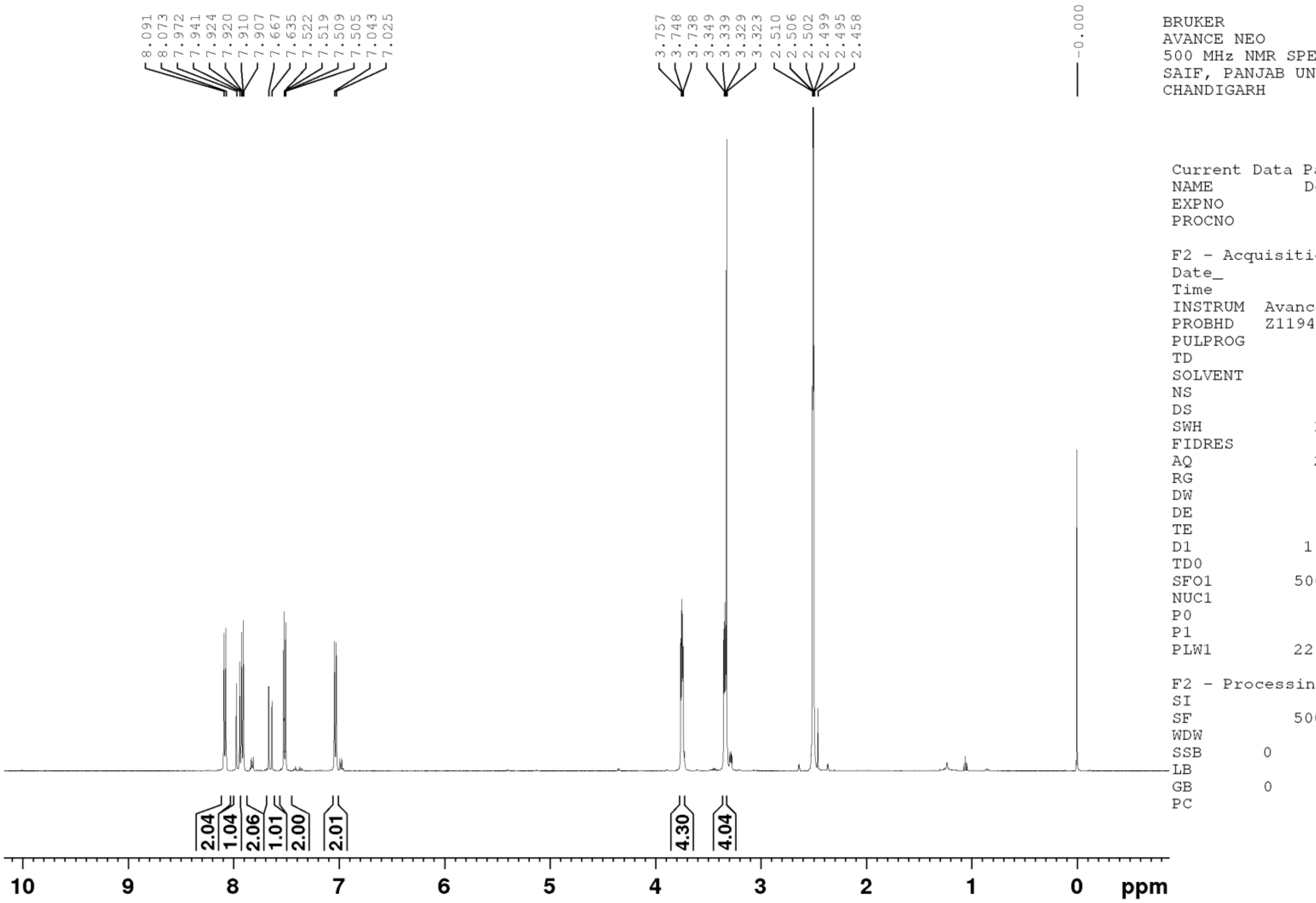

MO-8
1H_8scan DMSO {D:\Spectra} nmr 12

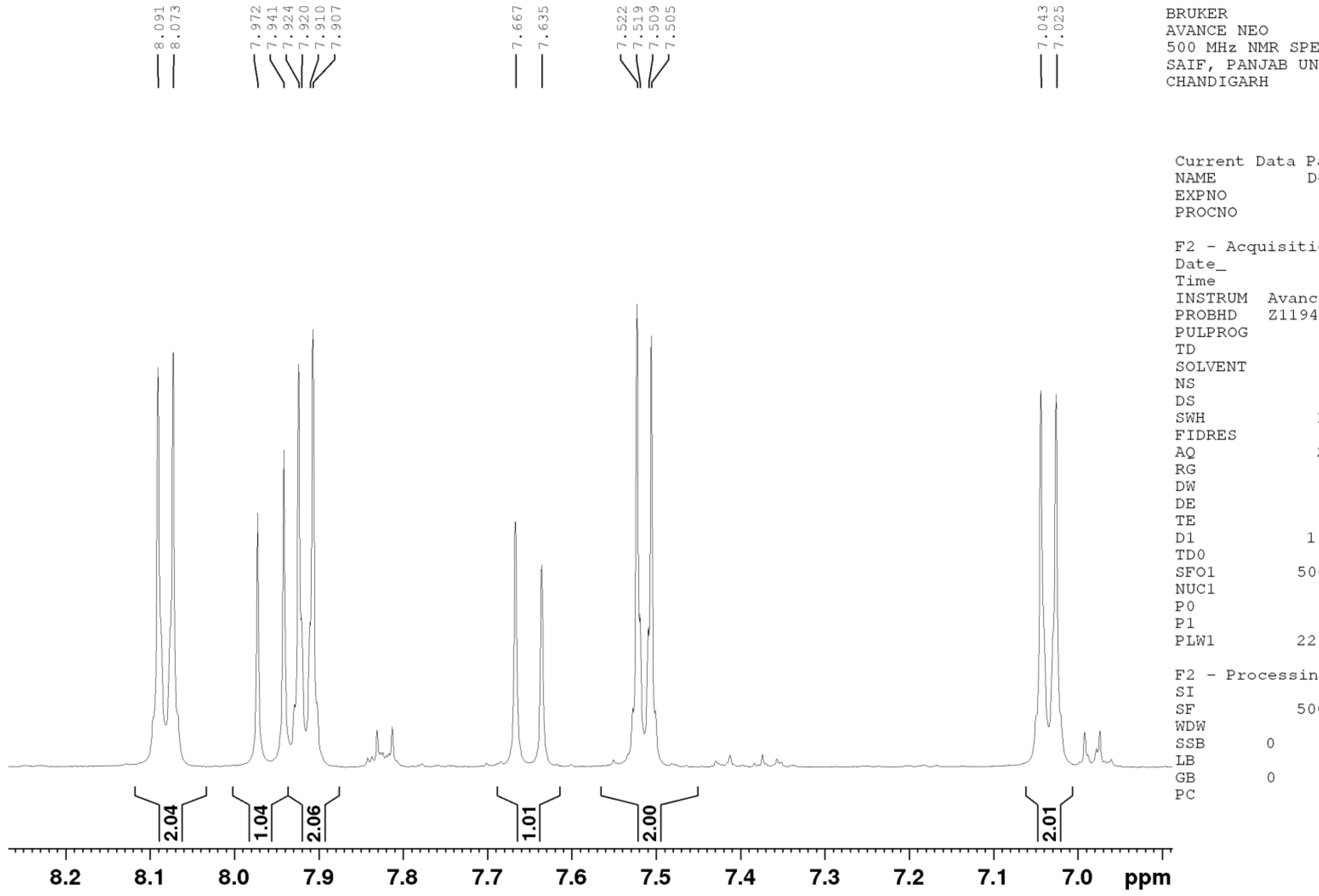

MO-8
1H_8scan DMSO {D:\Spectra} nmr 12

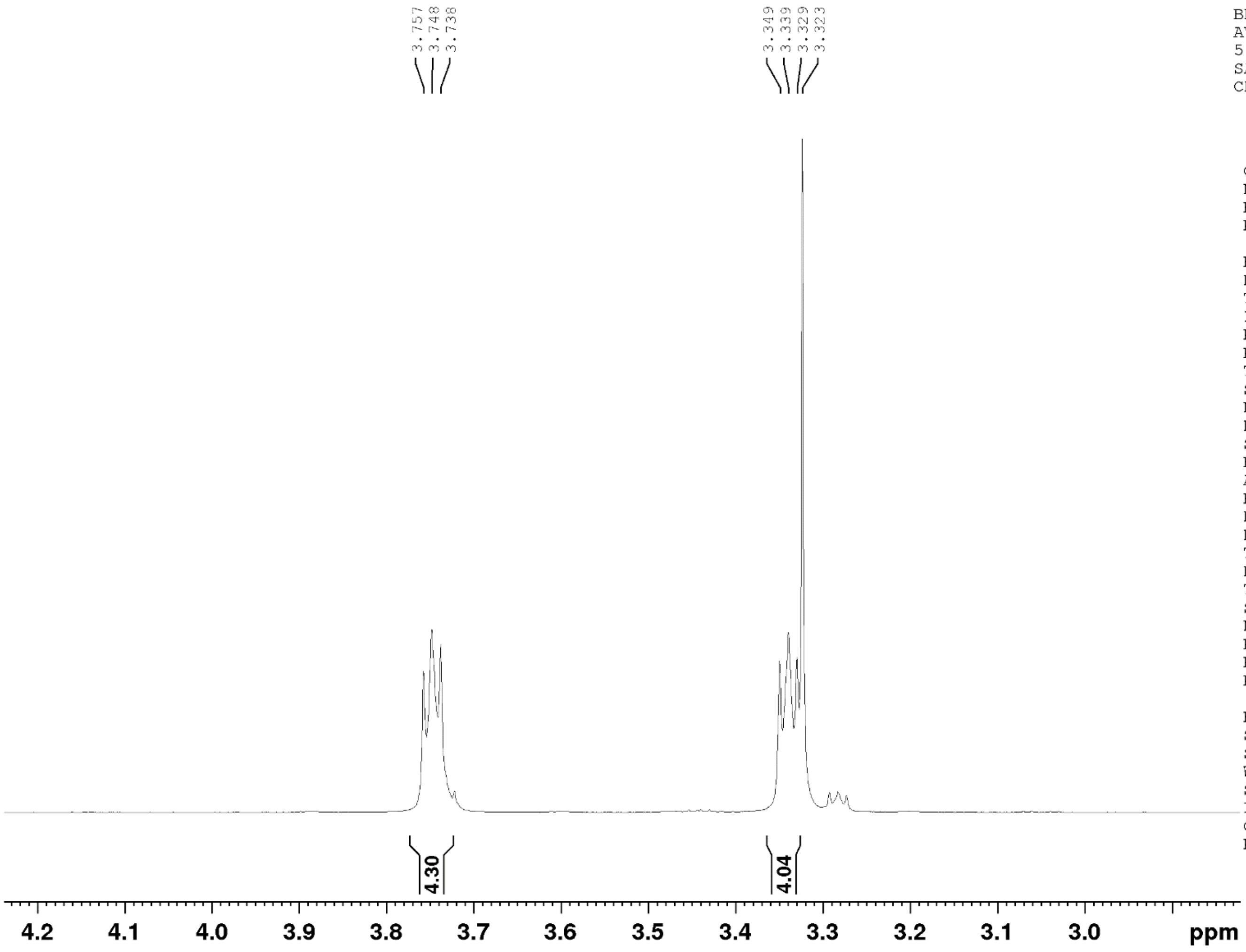

BRUKER
AVANCE NEO
500 MHz NMR SPE
SAIF, PANJAB UN
CHANDIGARH

Current Data P
NAME D
EXPNO
PROCNO

F2 - Acquisiti
Date_
Time
INSTRUM Avanc
PROBHD Z1194
PULPROG
TD
SOLVENT
NS
DS
SWH
FIDRES
AQ
RG
DW
DE
TE
D1 1
TD0
SFO1 50
NUC1
P0
P1
PLW1 22

F2 - Processin
SI
SF 50
WDW
SSB 0
LB
GB 0
PC

MO-8
C13CPD DMSO {D:\Spectra} nmr 12

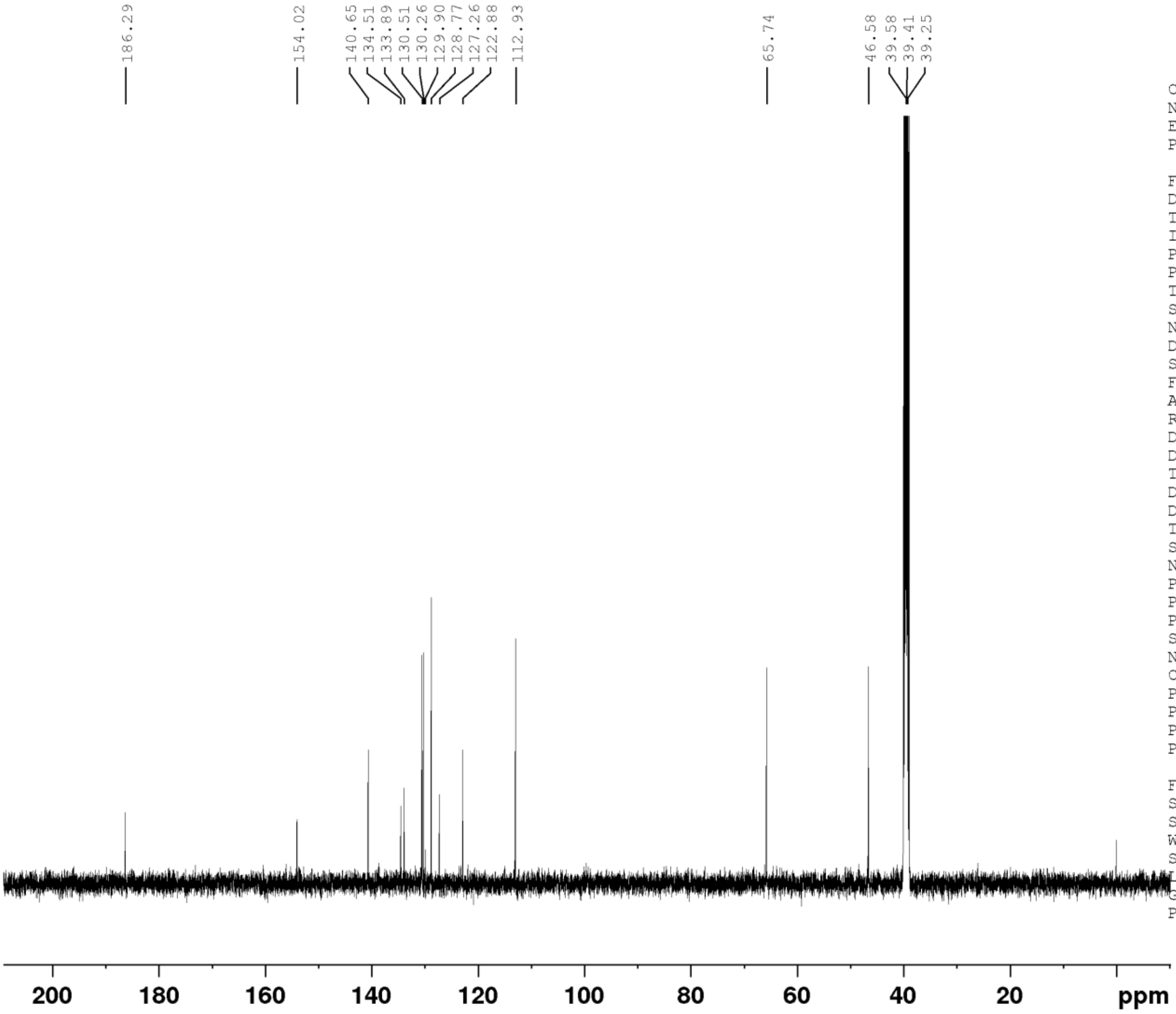

BRUKER
AVANCE NEO
500 MHz NMR SPECT
SAIF, PANJAB UNIV
CHANDIGARH

Current Data Parameters
NAME Dec24-2019
EXPNO 121
PROCNO 1

F2 - Acquisition Parameters
Date_ 20191224
Time 17.32 h
INSTRUM Avance Neo 500
PROBHD Z119470_0333 (
PULPROG zgpg30
TD 65536
SOLVENT DMSO
NS 512
DS 4
SWH 37037.035 Hz
FIDRES 1.130281 Hz
AQ 0.8847360 se
RG 101
DW 13.500 us
DE 6.50 us
TE 298.5 K
D1 2.00000000 se
D11 0.03000000 se
TD0 1
SFO1 125.7804233 MH
NUC1 13C
P0 3.33 us
P1 10.00 us
PLW1 79.56099701 W
SFO2 500.1720007 MH
NUC2 1H
CPDPRG[2 waltz65
PCPD2 80.00 us
PLW2 22.02300072 W
PLW12 0.34411001 W
PLW13 0.17308000 W

F2 - Processing parameters
SI 32768
SF 125.7679208 MH
WDW EM
SSB 0
LB 1.00 Hz
GB 0
PC 1.40

MO-8
C13CPD DMSO {D:\Spectra} nmr 12

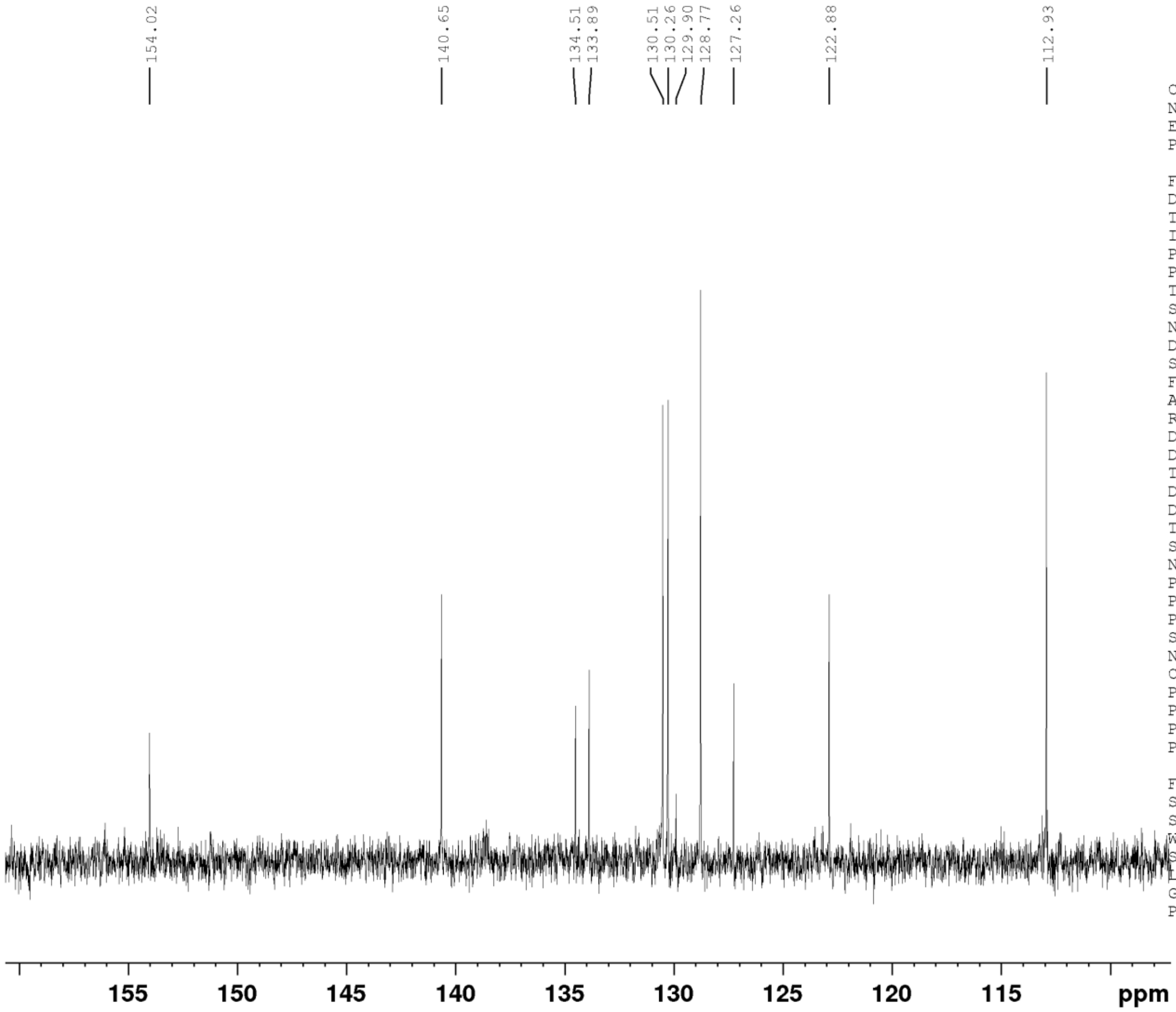

BRUKER
AVANCE NEO
500 MHz NMR SPECT
SAIF, PANJAB UNIV
CHANDIGARH

Current Data Parameters

NAME	Dec24-2019	
EXPNO	121	
PROCNO	1	

F2 - Acquisition Parameters

Date_	20191224	
Time	17.32	h
INSTRUM	Avance Neo 500	
PROBHD	Z119470_0333 (	
PULPROG	zgpg30	
TD	65536	
SOLVENT	DMSO	
NS	512	
DS	4	
SWH	37037.035	Hz
FIDRES	1.130281	Hz
AQ	0.8847360	se
RG	101	
DW	13.500	us
DE	6.50	us
TE	298.5	K
D1	2.00000000	sec
D11	0.03000000	sec
TD0	1	
SFO1	125.7804233	MH:
NUC1	13C	
P0	3.33	us
P1	10.00	us
PLW1	79.56099701	W
SFO2	500.1720007	MH:
NUC2	1H	
CPDPRG[2	waltz65	
PCPD2	80.00	us
PLW2	22.02300072	W
PLW12	0.34411001	W
PLW13	0.17308000	W

F2 - Processing parameters

SI	32768	
SF	125.7679208	MH:
WDW	EM	
SSB	0	
LB	1.00	Hz
GB	0	
PC	1.40	

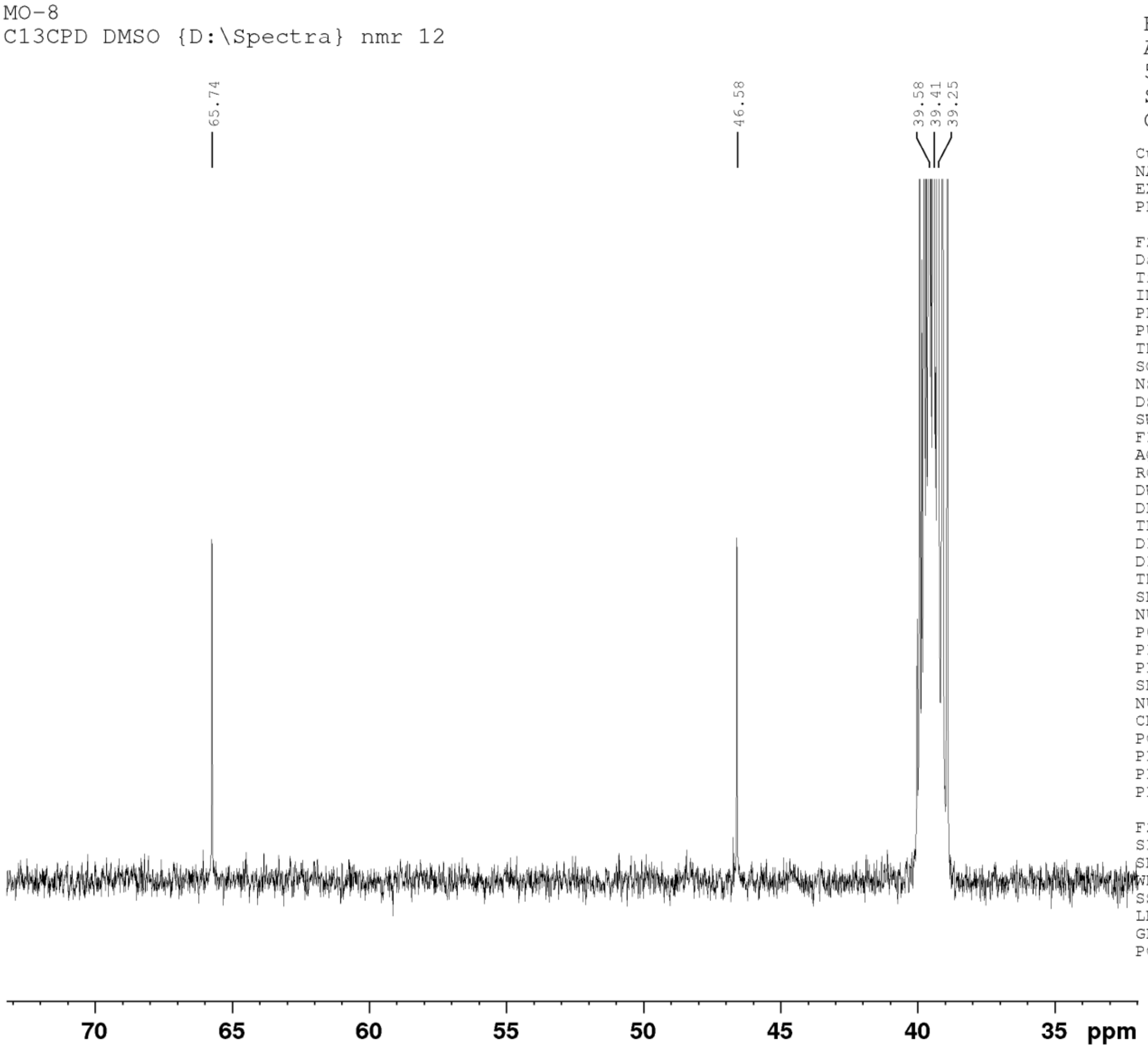

BRUKER
AVANCE NEO
500 MHz NMR SPECT
SAIF, PANJAB UNIV
CHANDIGARH

Current Data Parameters
NAME Dec24-2019
EXPNO 121
PROCNO 1

F2 - Acquisition Parameters
Date_ 20191224
Time 17.32 h
INSTRUM Avance Neo 500
PROBHD Z119470_0333 (
PULPROG zgpg30
TD 65536
SOLVENT DMSO
NS 512
DS 4
SWH 37037.035 Hz
FIDRES 1.130281 Hz
AQ 0.8847360 se
RG 101
DW 13.500 us
DE 6.50 us
TE 298.5 K
D1 2.00000000 se
D11 0.03000000 se
TD0 1
SFO1 125.7804233 MH
NUC1 13C
P0 3.33 us
P1 10.00 us
PLW1 79.56099701 W
SFO2 500.1720007 MH
NUC2 1H
CPDPRG[2 waltz65
PCPD2 80.00 us
PLW2 22.02300072 W
PLW12 0.34411001 W
PLW13 0.17308000 W

F2 - Processing parameters
SI 32768
SF 125.7679208 MH
WDW EM
SSB 0
LB 1.00 Hz
GB 0
PC 1.40

MO-9
1H_8scan DMSO {D:\Spectra} nmr 13

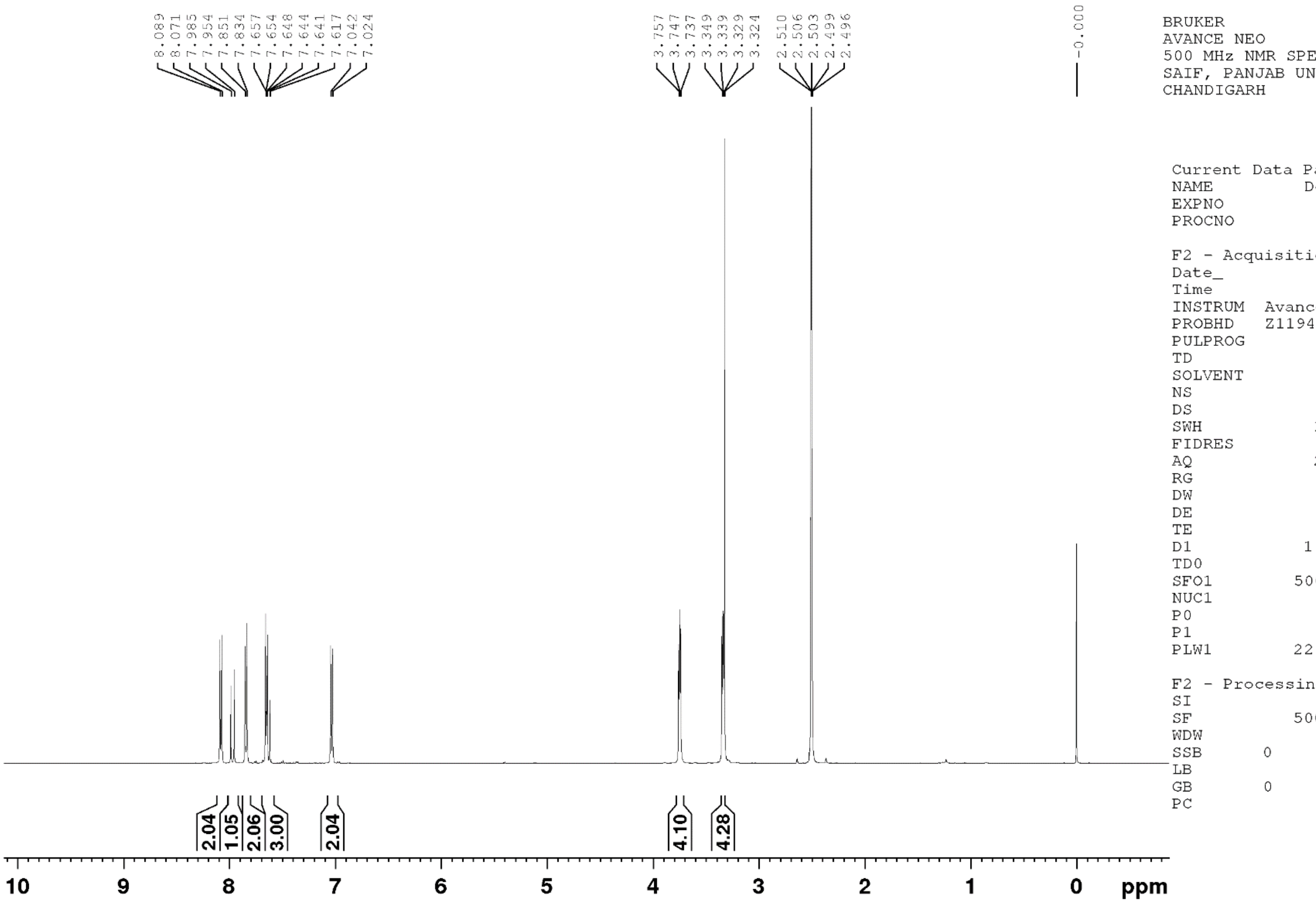

MO-9
1H_8scan DMSO {D:\Spectra} nmr 13

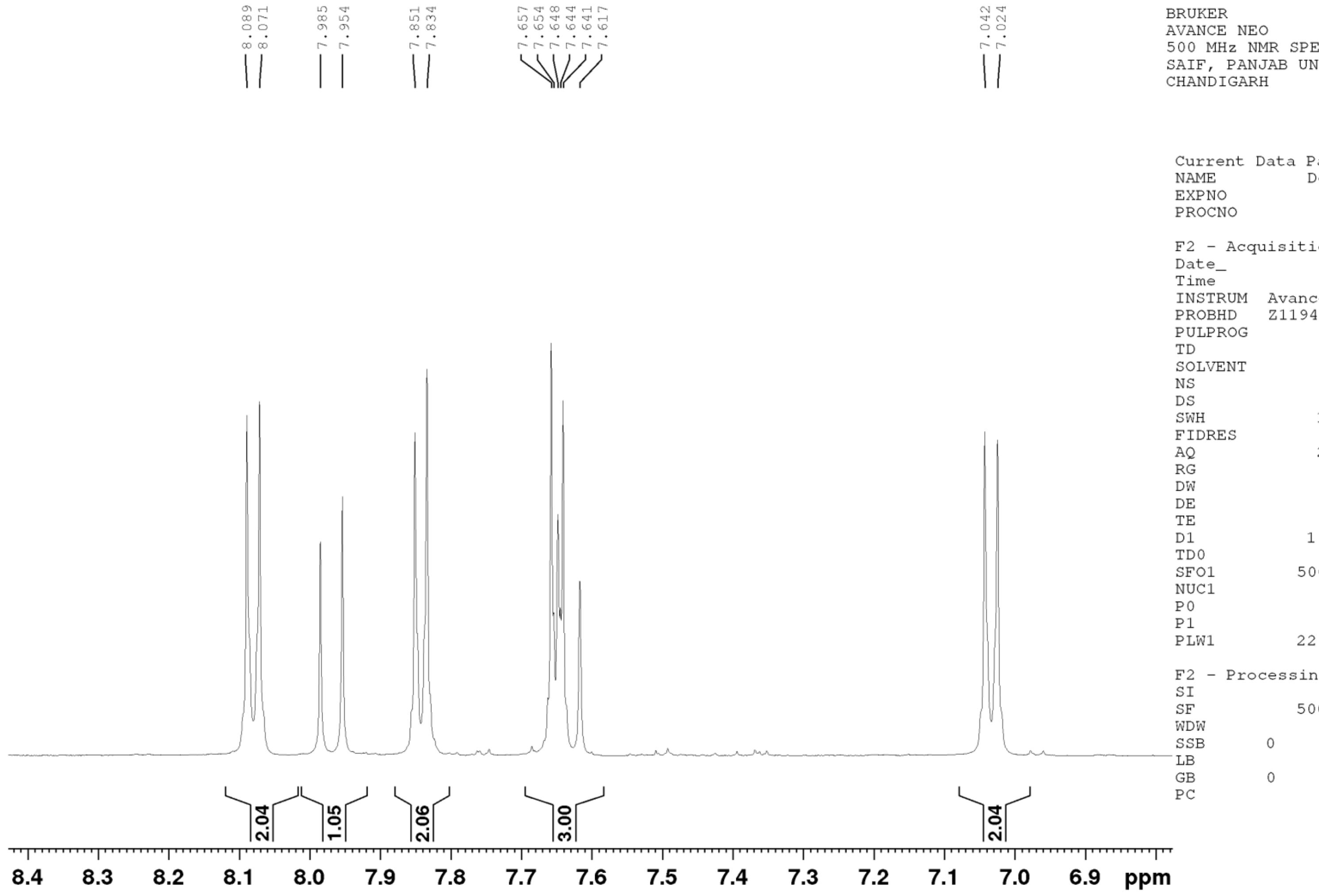

MO-9
1H_8scan DMSO {D:\Spectra} nmr 13

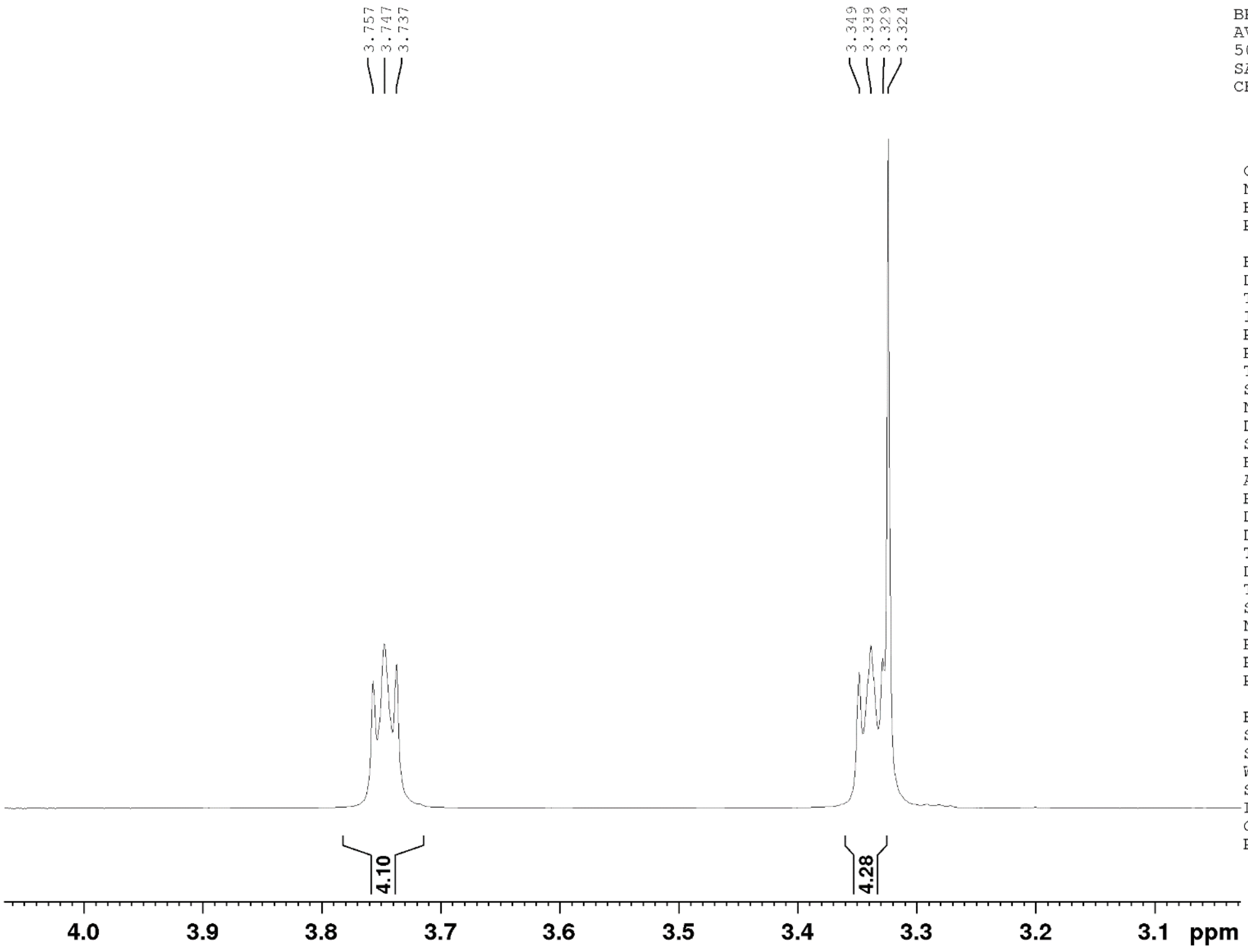

BRUKER
AVANCE NEO
500 MHz NMR SPE
SAIF, PANJAB UN
CHANDIGARH

Current Data P
NAME D
EXPNO
PROCNO

F2 - Acquisiti
Date_
Time
INSTRUM Avanc
PROBHD Z1194
PULPROG
TD
SOLVENT
NS
DS
SWH
FIDRES
AQ
RG
DW
DE
TE
D1 1
TD0
SFO1 50
NUC1
P0
P1
PLW1 22

F2 - Processin
SI
SF 50
WDW
SSB 0
LB
GB 0
PC

MO-9
C13CPD DMSO {D:\Spectra} nmr 13

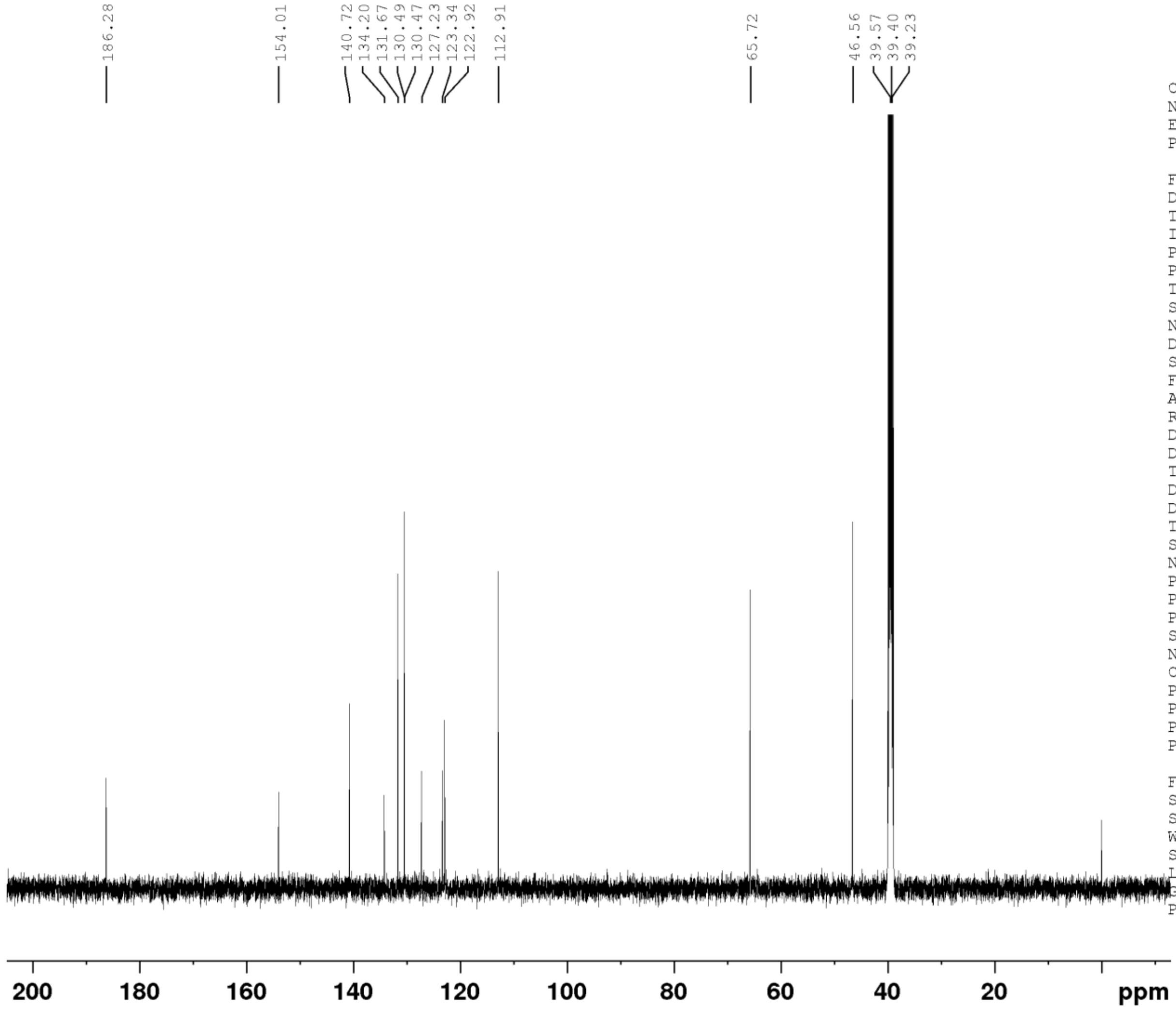

BRUKER
AVANCE NEO
500 MHz NMR SPECT
SAIF, PANJAB UNIV
CHANDIGARH

Current Data Parameters
NAME Dec24-2019
EXPNO 131
PROCNO 1

F2 - Acquisition Parameters
Date_ 20191224
Time 18.00 h
INSTRUM Avance Neo 500
PROBHD Z119470_0333 (
PULPROG zgpg30
TD 65536
SOLVENT DMSO
NS 512
DS 4
SWH 37037.035 Hz
FIDRES 1.130281 Hz
AQ 0.8847360 se
RG 101
DW 13.500 us
DE 6.50 us
TE 298.3 K
D1 2.00000000 se
D11 0.03000000 se
TD0 1
SFO1 125.7804233 MH
NUC1 13C
P0 3.33 us
P1 10.00 us
PLW1 79.56099701 W
SFO2 500.1720007 MH
NUC2 1H
CPDPRG[2 waltz65
PCPD2 80.00 us
PLW2 22.02300072 W
PLW12 0.34411001 W
PLW13 0.17308000 W

F2 - Processing parameters
SI 32768
SF 125.7679227 MH
WDW EM
SSB 0
LB 1.00 Hz
GB 0
PC 1.40

MO-9
C13CPD DMSO {D:\Spectra} nmr 13

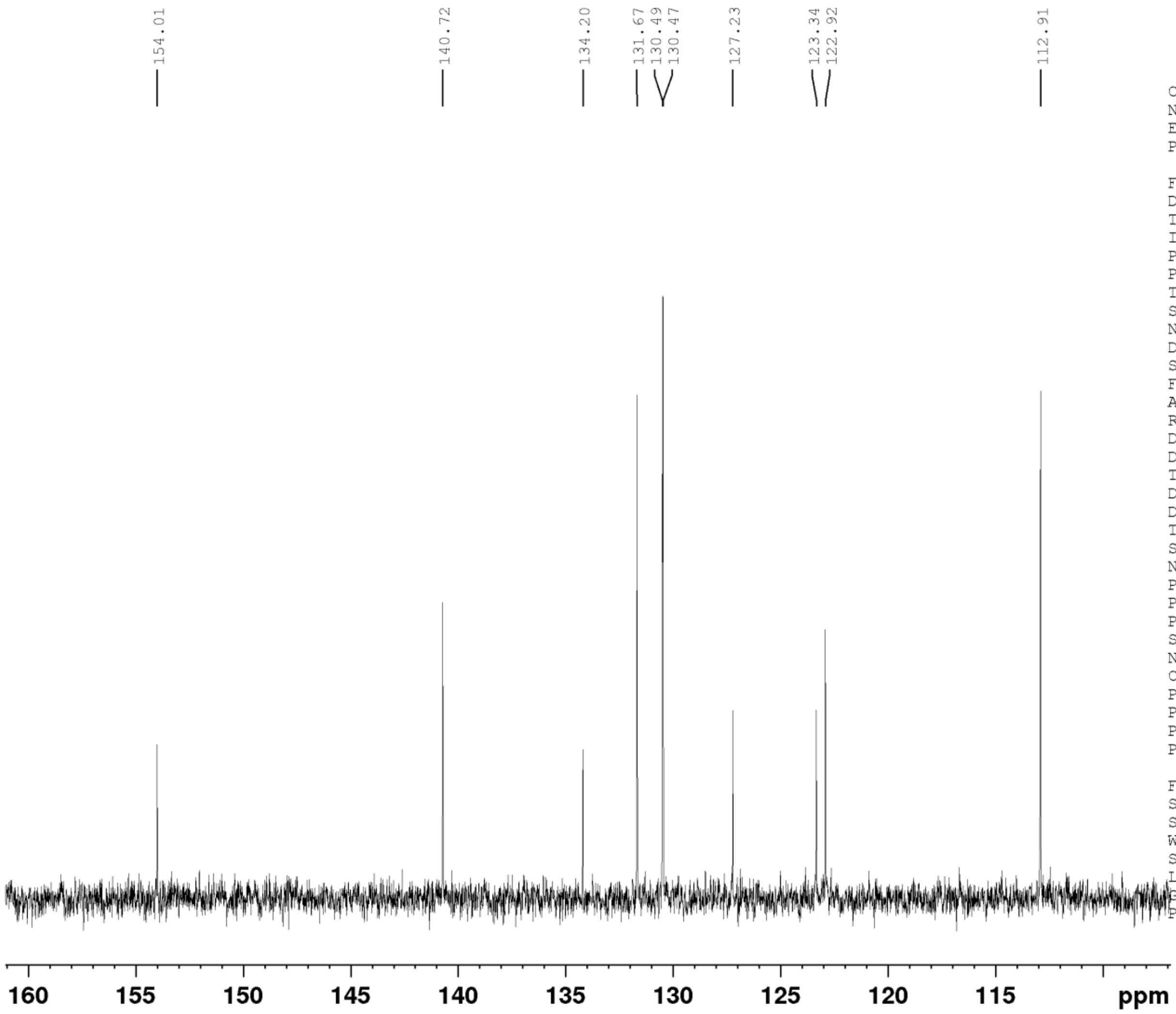

BRUKER
AVANCE NEO
500 MHz NMR SPECT
SAIF, PANJAB UNIV
CHANDIGARH

Current Data Parameters
NAME Dec24-2019
EXPNO 131
PROCNO 1

F2 - Acquisition Parameters
Date_ 20191224
Time 18.00 h
INSTRUM Avance Neo 500
PROBHD Z119470_0333 (
PULPROG zgpg30
TD 65536
SOLVENT DMSO
NS 512
DS 4
SWH 37037.035 Hz
FIDRES 1.130281 Hz
AQ 0.8847360 se
RG 101
DW 13.500 us
DE 6.50 us
TE 298.3 K
D1 2.00000000 sec
D11 0.03000000 sec
TD0 1
SFO1 125.7804233 MHz
NUC1 13C
P0 3.33 us
P1 10.00 us
PLW1 79.56099701 W
SFO2 500.1720007 MHz
NUC2 1H
CPDPRG[2 waltz65
PCPD2 80.00 us
PLW2 22.02300072 W
PLW12 0.34411001 W
PLW13 0.17308000 W

F2 - Processing parameters
SI 32768
SF 125.7679227 MHz
WDW EM
SSB 0
LB 1.00 Hz
GB 0
PC 1.40

MO-9
C13CPD DMSO {D:\Spectra} nmr 13

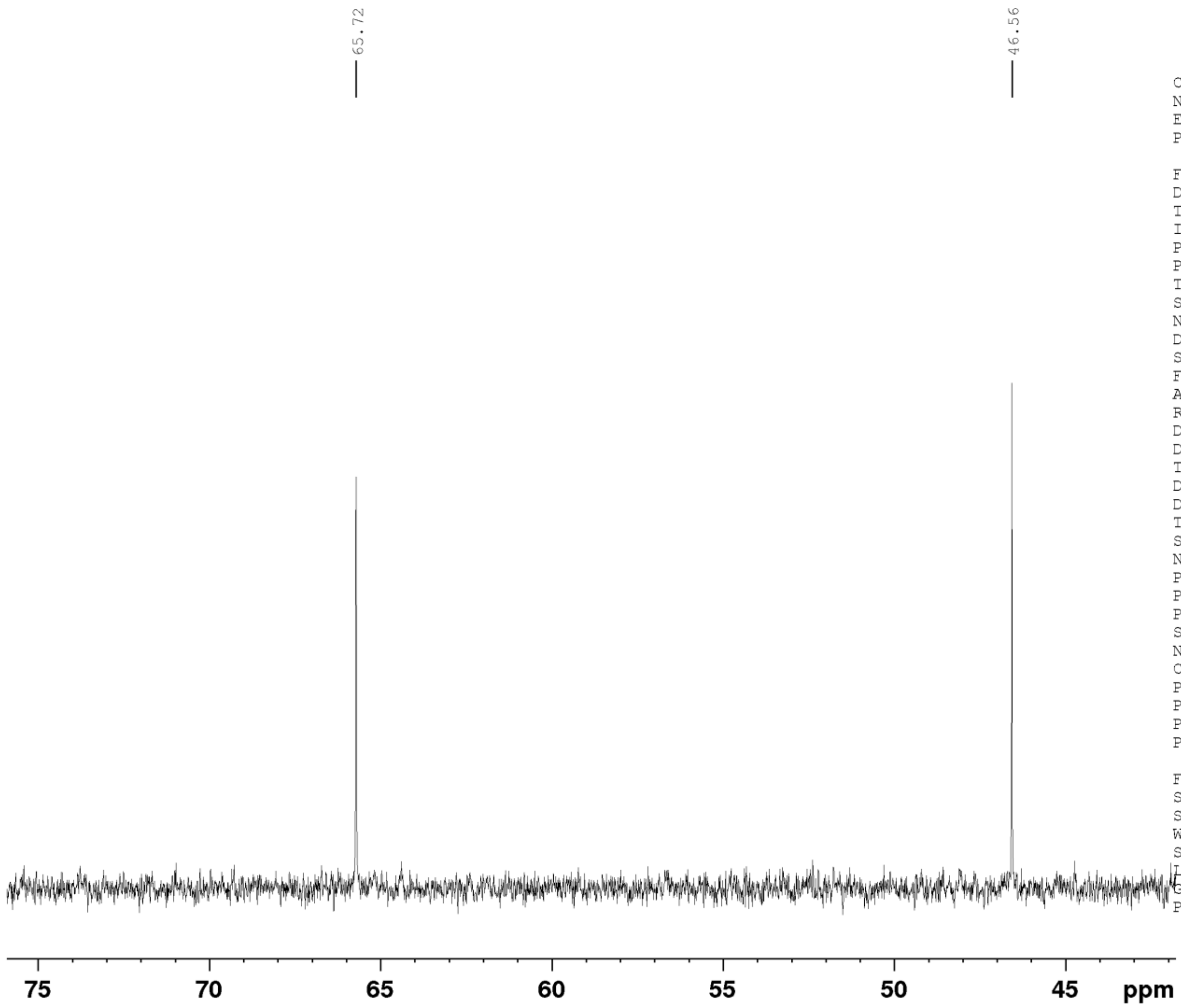

BRUKER
AVANCE NEO
500 MHz NMR SPECT
SAIF, PANJAB UNIV
CHANDIGARH

Current Data Parameters

NAME	Dec24-2019
EXPNO	131
PROCNO	1

F2 - Acquisition Parameters

Date_	20191224
Time	18.00 h
INSTRUM	Avance Neo 500
PROBHD	Z119470_0333 (
PULPROG	zgpg30
TD	65536
SOLVENT	DMSO
NS	512
DS	4
SWH	37037.035 Hz
FIDRES	1.130281 Hz
AQ	0.8847360 se
RG	101
DW	13.500 us
DE	6.50 us
TE	298.3 K
D1	2.00000000 sec
D11	0.03000000 sec
TD0	1
SFO1	125.7804233 MH
NUC1	13C
P0	3.33 us
P1	10.00 us
PLW1	79.56099701 W
SFO2	500.1720007 MH
NUC2	1H
CPDPRG[2	waltz65
PCPD2	80.00 us
PLW2	22.02300072 W
PLW12	0.34411001 W
PLW13	0.17308000 W

F2 - Processing parameters

SI	32768
SF	125.7679227 MH
WDW	EM
SSB	0
LB	1.00 Hz
GB	0
PC	1.40

MO1

Scan: 775 TIC=1917663 Base=7.8%FS #ions=479 RT=19.45

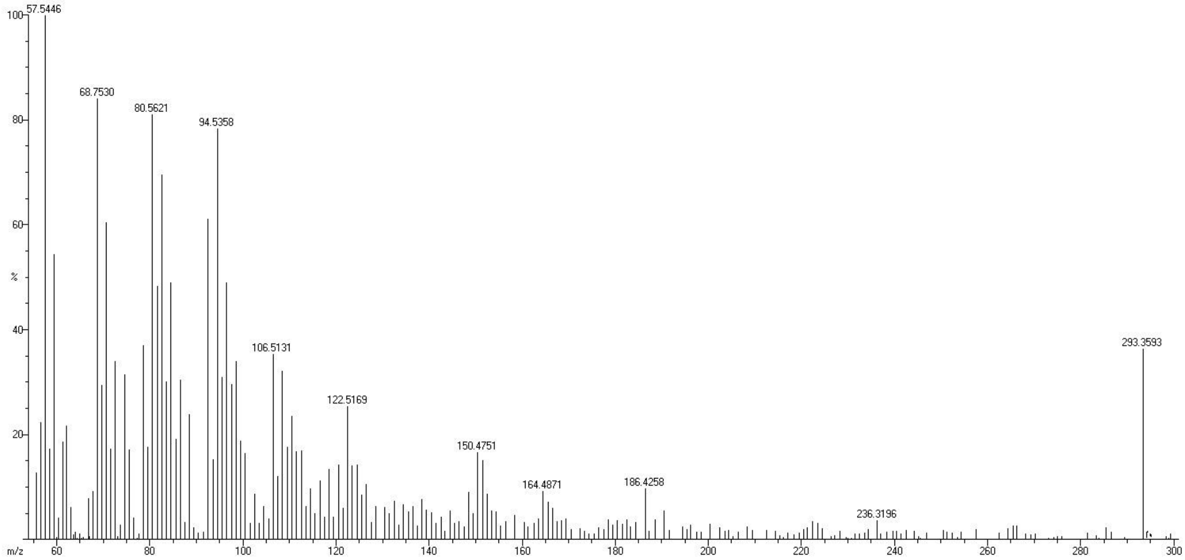

MO3

Scan: 826 TIC=1459208 Base=6.9%FS #ions=456 RT=20.72

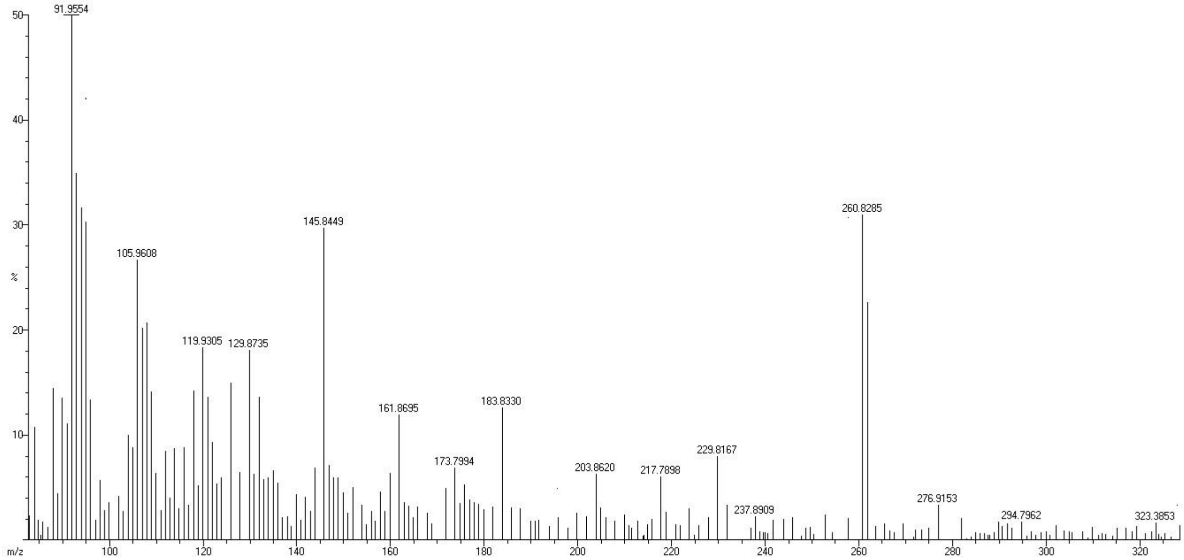

MO4

Scan: 863 TIC=1276126 Base=8.9%FS #ions=462 RT=21.65

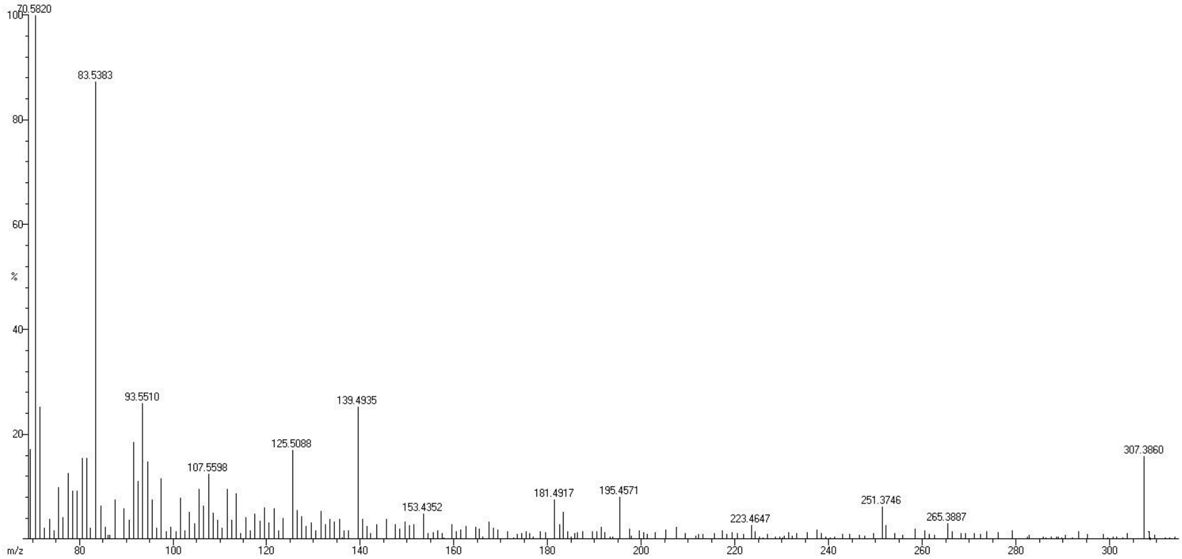

MO5

Scan: 656 TIC=776054 Base=5.6%FS #ions=486 RT=16.45

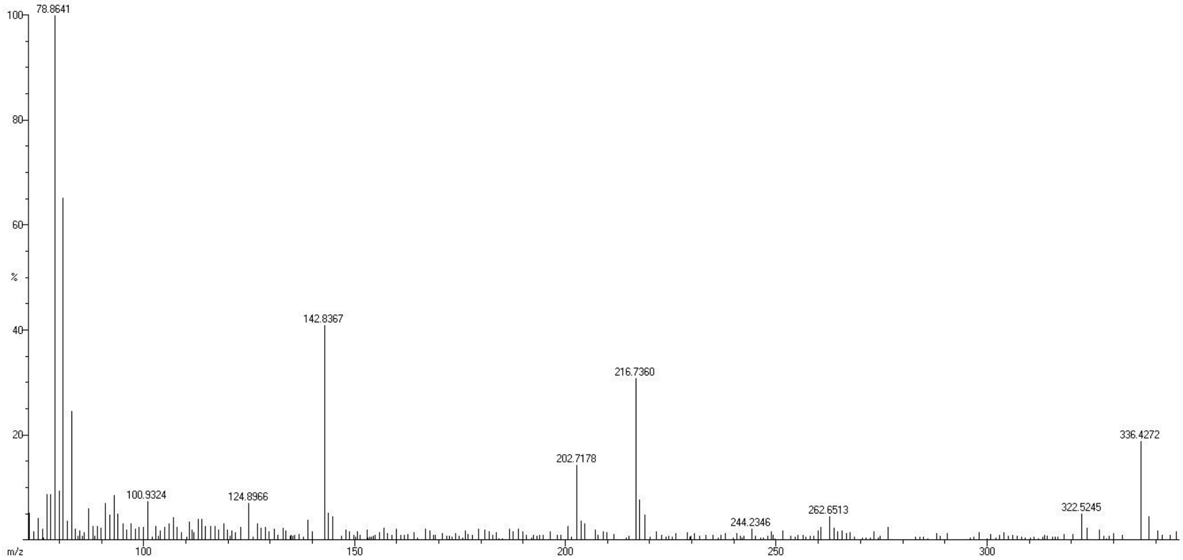

MO6

Scan: 672 TIC=906837 Base=4.8%FS #ions=501 RT=16.85

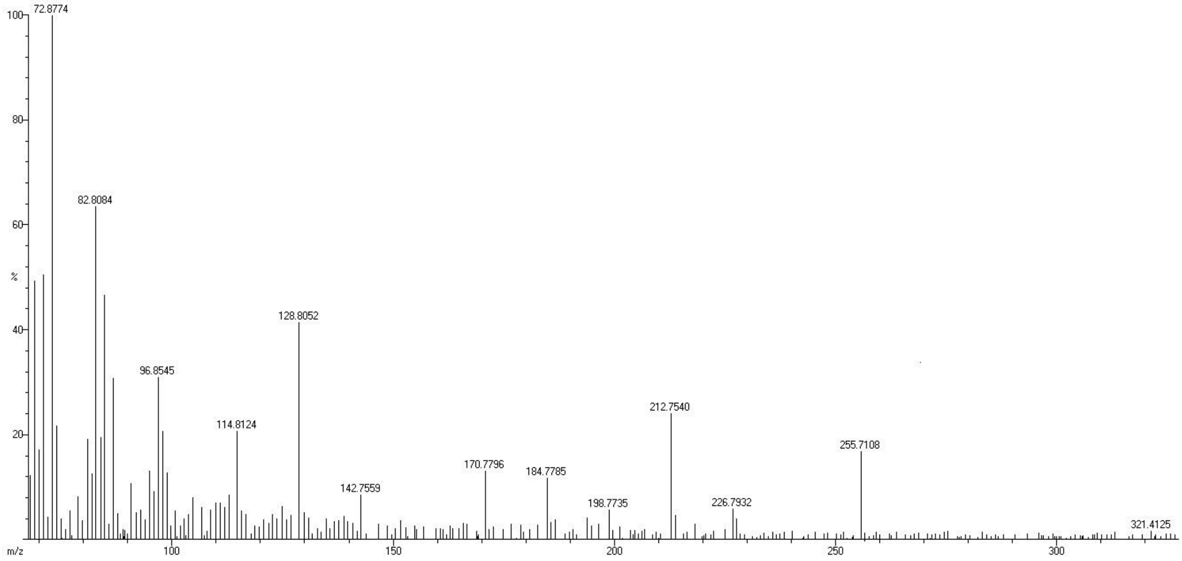

MO7

Scan: 713 TIC=1380688 Base=5.9%FS #ions=432 RT=17.88

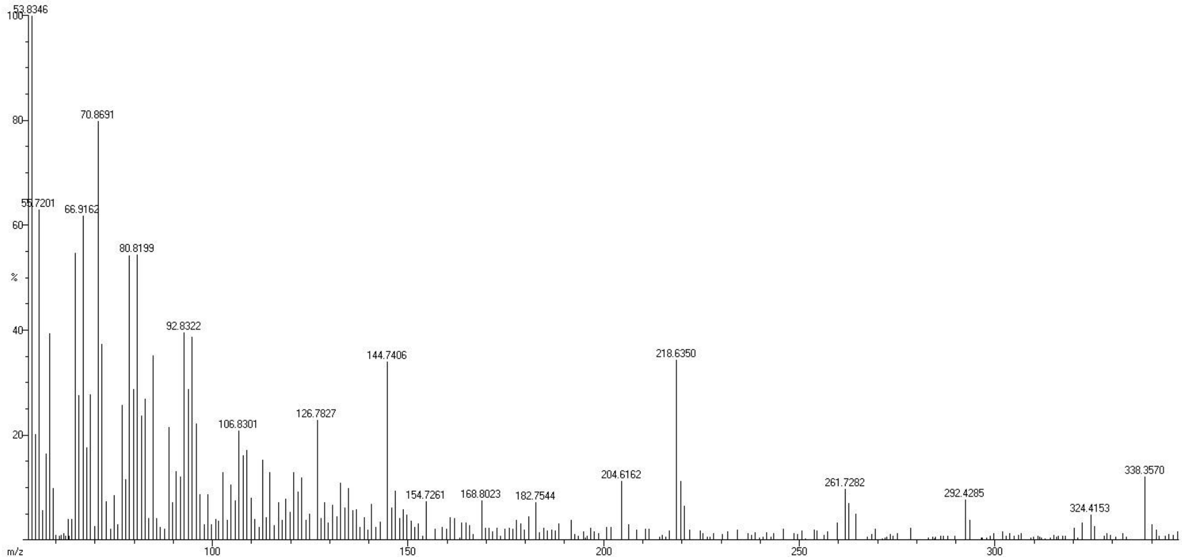

MO8

Scan: 813 TIC=1942309 Base=9%FS #ions=437 RT=20.4

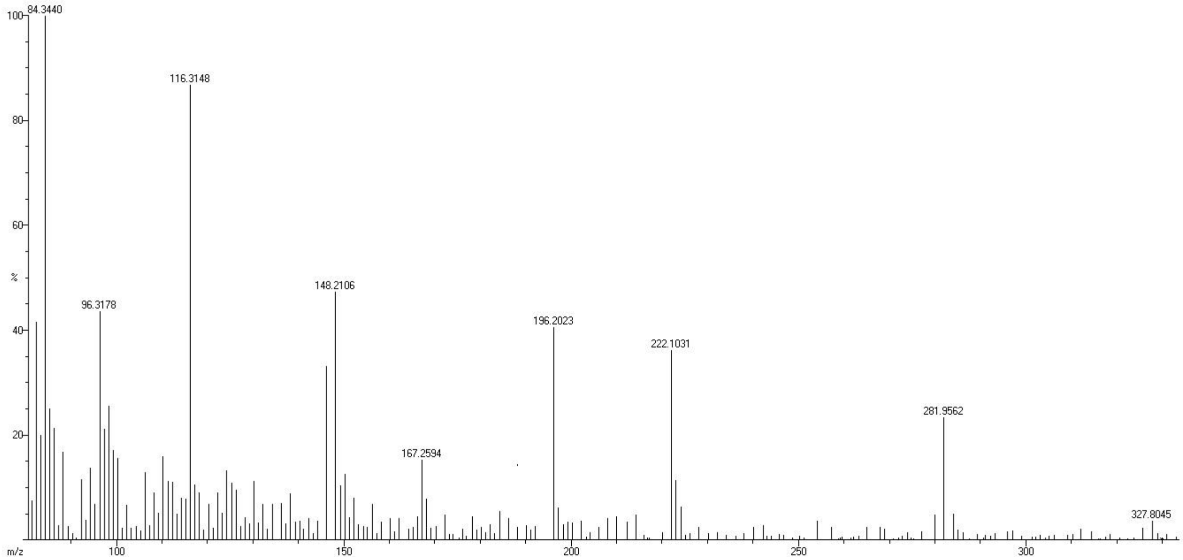

MO9

Scan: 764 TIC=2008430 Base=7.8%FS #ions=414 RT=19.17

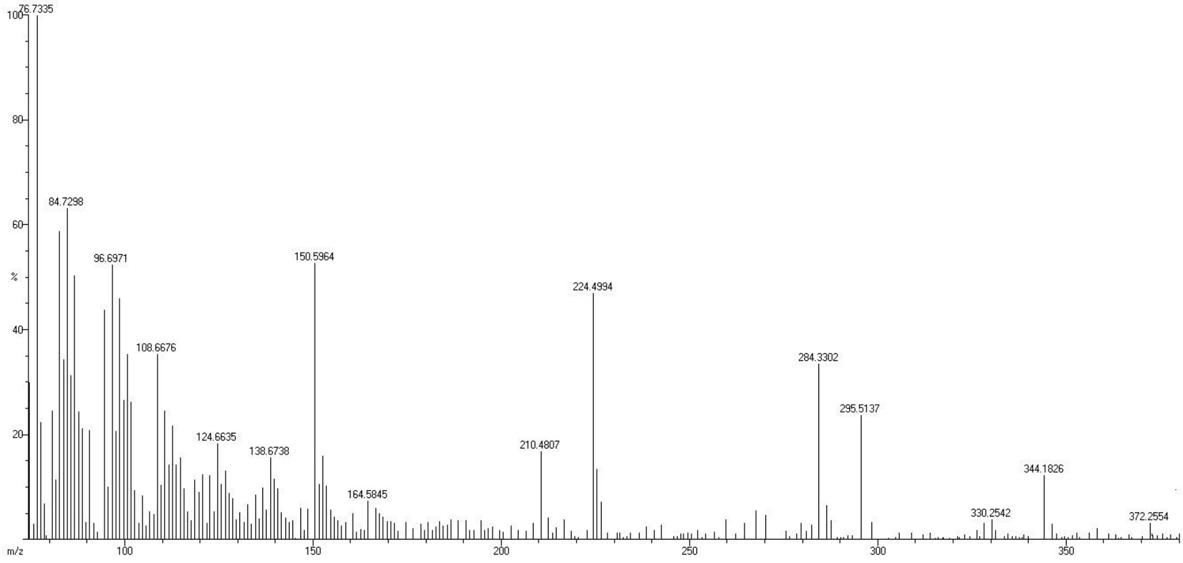

MuSSel prediction of the lead molecules……………………………………………………………………S3

MuSSel prediction of the lead MO1

ligando

Prediction for ligando: O=C(/C=C/c1ccccc1)c3ccc(N2CCOCC2)cc3

Position	Target	Score	Reliability	Similars
1	Calpain 2:Sus scrofa	91.683 % (11.919)	YES	1
2	Caspase-3:Homo sapiens	75.868 % (9.863)	YES	5
3	Sentrin-specific protease 7:Homo sapiens	75.868 % (9.863)	YES	5
4	Sentrin-specific protease 8:Homo sapiens	75.868 % (9.863)	YES	5
5	Sentrin-specific protease 6:Homo sapiens	75.868 % (9.863)	YES	5
6	Glycogen synthase kinase-3 beta:Homo sapiens	60.719 % (7.894)	YES	3
7	Arachidonate 5-lipoxygenase:Rattus norvegicus	57.694 % (7.500)	YES	5
8	Cathepsin L:Homo sapiens	50.677 % (6.588)	YES	5
9	Cathepsin B:Homo sapiens	50.677 % (6.588)	YES	5
10	DNA-dependent protein kinase:Homo sapiens	45.533 % (5.919)	NO	4
11	Protein skinhead-1:Caenorhabditis elegans	44.933 % (5.841)	NO	7
12	Protein-tyrosine phosphatase 1B:Homo sapiens	40.958 % (5.325)	NO	5
13	Monoamine oxidase B:Homo sapiens	40.916 % (5.319)	NO	7
14	Acetylcholinesterase:Homo sapiens	39.023 % (5.073)	NO	7
15	Integrase:Human immunodeficiency virus 1	37.149 % (4.829)	NO	4
16	Heat shock protein Hsp-16.2:Caenorhabditis elegans	34.073 % (4.430)	NO	1
17	Beta Lactamase:Pseudomonas aeruginosa	34.060 % (4.428)	NO	7
18	PI3-kinase p110-delta subunit:Homo sapiens	34.007 % (4.421)	NO	2
19	PI3-kinase p110-gamma subunit:Homo sapiens	34.007 % (4.421)	NO	2
20	PI3-kinase p110-beta subunit:Homo sapiens	34.007 % (4.421)	NO	2

MuSSel prediction of the lead MO5

ligando

Prediction for ligando: CN(C)c3ccc(/C=C/C(=O)c2ccc(N1CCOCC1)cc2)cc3

Position	Target	Score	Reliability	Similars
1	Calpain 2:Sus scrofa	86.747 % (11.277)	YES	1
2	Sentrin-specific protease 6:Homo sapiens	65.432 % (8.506)	YES	5
3	Sentrin-specific protease 8:Homo sapiens	65.432 % (8.506)	YES	5
4	Caspase-3:Homo sapiens	65.432 % (8.506)	YES	5
5	Sentrin-specific protease 7:Homo sapiens	65.432 % (8.506)	YES	5
6	Arachidonate 5-lipoxygenase:Rattus norvegicus	53.401 % (6.942)	YES	4
7	Monoamine oxidase B:Homo sapiens	47.277 % (6.146)	YES	9
8	Protein-tyrosine phosphatase 1B:Homo sapiens	46.935 % (6.102)	YES	3
9	Cathepsin L:Homo sapiens	44.673 % (5.807)	NO	6
10	Cathepsin B:Homo sapiens	44.673 % (5.807)	NO	7
11	Beta Lactamase:Pseudomonas aeruginosa	43.832 % (5.698)	NO	7
12	Acetylcholinesterase:Electrophorus electricus	43.187 % (5.614)	NO	9
13	Monoamine oxidase A:Homo sapiens	42.763 % (5.559)	NO	5
14	Protein skinhead-1:Caenorhabditis elegans	41.656 % (5.415)	NO	6
15	Acetylcholinesterase:Homo sapiens	39.220 % (5.099)	NO	8
16	Cyclooxygenase-2:Homo sapiens	38.388 % (4.990)	NO	5
17	Toll-like receptor 9:Homo sapiens	37.371 % (4.858)	NO	6
18	Quinolone resistance protein norA:Staphylococcus aureus	35.691 % (4.640)	NO	3
19	Glycogen synthase kinase-3 beta:Homo sapiens	34.780 % (4.521)	NO	3
20	Cholinesterase:Equus caballus	34.515 % (4.487)	NO	8
